# Development of Praziquantel sulphonamide derivatives as antischistosomal drugs

**DOI:** 10.1080/14756366.2022.2078970

**Published:** 2022-05-29

**Authors:** Andrea Angeli, Marta Ferraroni, Fabrizio Carta, Cécile Häberli, Jennifer Keiser, Gabriele Costantino, Claudiu T. Supuran

**Affiliations:** a NEUROFARBA Department, Sezione di Scienze Farmaceutiche, University of Florence, Florence, Italy; b Department of Food and Drug, University of Parma, Parco Area delle Scienze, Parma, Italy; c Dipartimento di Chimica “Ugo Schiff”, University of Florence, Florence, Italy; d Department of Medical Parasitology and Infection Biology, Swiss Tropical and Public Health Institute, Allschwil, Switzerland; e University of Basel, Basel, Switzerland

**Keywords:** Carbonic anhydrase, metalloenzymes, schistosomiasis, sulphonamide, praziquantel, *Schistosoma mansoni*

## Abstract

The almost empty armamentarium to treat schistosomiasis, a neglected parasitic disorder caused by trematode flatworms of the genus *Schistosoma*, except Praziquantel (PZQ), urged to find new alternatives to fight this infection. Carbonic Anhydrase from *Schistosoma mansoni* (SmCA) is a possible new target against this nematode. Here, we propose new PZQ derivatives bearing a primary sulphonamide group in order to obtain hybrid drugs. All compounds were evaluated for their inhibition profiles on both humans and Schistosoma CAs, X-ray crystal data of SmCA and hCA II in adduct with some inhibitors were obtained allowing the understanding of the main structural factors responsible of activity. The compounds showed *in vitro* inhibition of immature and adult *S. mansoni*, but further optimisation is required for improved activity.

## 1. Introduction

Schistosomiasis is a water-based disease and the second most important neglected parasitic disorder caused by trematode flatworms of the genus *Schistosoma* affecting more than 200 million people worldwide, mainly in developing countries of tropical and subtropical areas^1^. In the last decades, despite great efforts devoted to the development of vaccines for schistosomiasis, the current treatment and control strategy relies on a single drug, the pyrazinoisoquinoline derivative praziquantel (PZQ)^2^^,^^3^. For this reason, recently, in its roadmap to “eliminate schistosomiasis as a public health problem by 2030”, the World Health Organisation (WHO) calls for the development of new interventions, including alternatives to PZQ^4^. The anthelmintic drug PZQ was approved in 1980 and, after 40 years, no other therapeutic option for treating schistosomiasis has achieved this goal. Although PZQ is reasonably safe and effective^5^^,^^6^, the drug has notable suboptimal features such variable cure rates following single dose treatment^7^^,^^8^ or limited efficacy against immature parasites making repeated administration necessary^9^^,^^10^. The empty armamentarium except PZQ to treat schistosomiasis might trigger a risk of resistance development to PZQ by the different *Schistosoma spp.*^11–13^. The development of alternative treatments is clearly desirable and, in the last years, a potentially attractive alternative schistosome drug target namely the inhibition of parasitic Carbonic Anhydrase (CA, E.C. 4.2.1.1) was reported. CAs were shown to be essential for the worms to establish a robust infection in experimental animals and findings strongly suggests that chemical inhibition of CA from *S. mansoni* (SmCA) debilitate the worms and curtail the infection^14^. CAs are a superfamily of ubiquitous zinc metalloenzymes, present in all kingdoms of life, and encoded by at least eight distinct, evolutionarily unrelated gene families (designated *α*, *β*, *γ*, *δ*, *ζ*, *η*, *θ* and *ι*) converging in the same crucial reaction, the reverse hydration of CO_2_ to H^+^ and HCO_3_^−^^15–18^. Recently our group reported the key molecular features of CA inhibitors (CAIs) binding to SmCA in comparison with human hCA II in order to understand the structural determinants directing affinity and selectivity^19^^,^^20^. Here, we report on the development of SmCA inhibitors conjugated to the PZQ scaffold as a possible novel therapeutic target for schistosomiasis.

## 2. Results and discussion

### 2.1. Compounds design and synthesis

**Figure 1.**
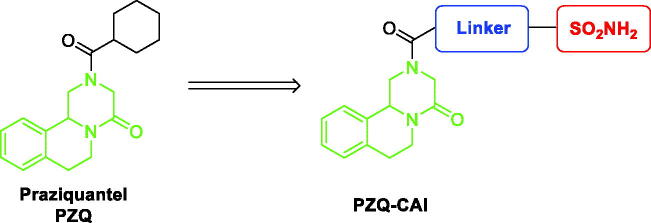
General structure of novel hybrid PZQ-CAI.

In the context of a multitarget approach, we have focussed on the still unknown target of PZQ, even if recent efforts indicate as possible target the disruption of calcium ion homeostasis in the worm antagonising the voltage-gated calcium channels^21^^,^^22^ and the inhibition of SmCA. To achieve this, we have hybridised the pyrazino isoquinolinone moiety of PZQ with a primary sulphonamide moiety employing different linkers to obtain novel PZQ-CAI derivatives (Figure 1).

**Scheme 1.**
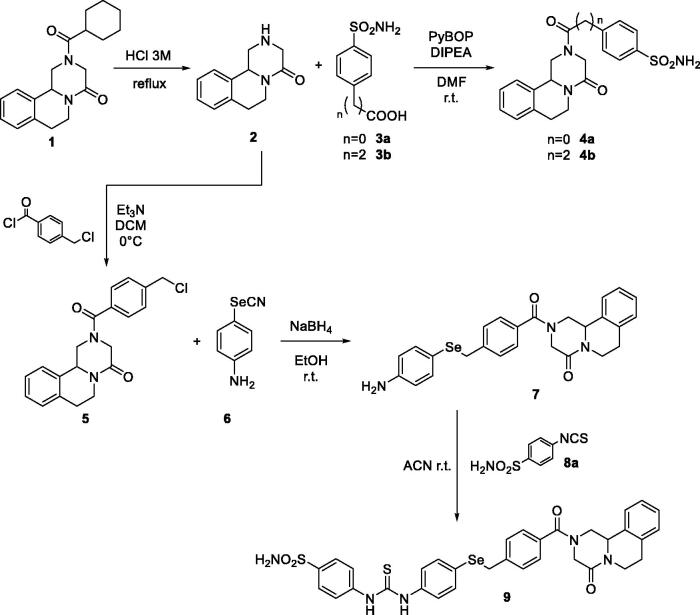
General synthesis of derivatives **4–9.**

In the first step, we explored the possibility to connect the benzenesulfonamide moiety directly employing PZQ (**1**) as starting material and, after hydrolysis, two different carboxylic acids with sulphonamide group (**3a–b**) are used in a classical coupling reaction to obtain the first hybrid PZQ-CAI **4a** and **4b** in good yield as described in Scheme 1. Subsequently, we started to synthetise different linkers using derivative **2** as starting point. Initially, a nucleophilic substitution with 4-(chloromethyl)benzoyl chloride (**5**) and subsequently reacting with compound **6** using NaBH_4_ achieved amino derivative **7**. It was given the possibility to react with isothiocyanate sulphonamide **8a** to obtain the thioureido derivative with selenium linker **9 (**Scheme 1).

**Scheme 2.**
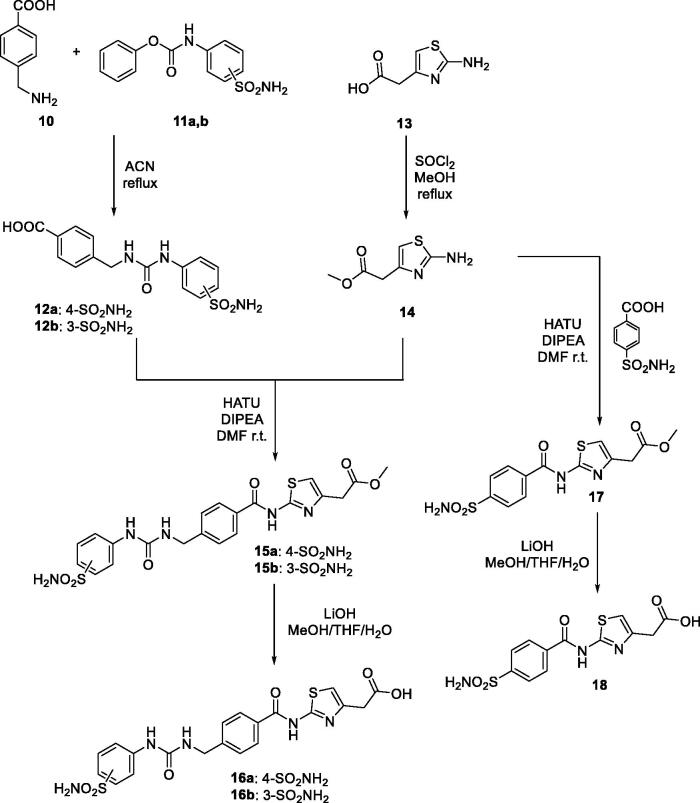
General synthesis of derivatives **12–18**.

In order to obtain different linkers coupled with sulphonamide moiety, we synthetised two ureido derivatives **12a,b** by direct aminolysis of phenylcarbamates **11a,b** with 4-(aminomethyl)benzoic acid **10** as reported in Scheme 2. Subsequently, derivatives **12a,b** reacted with compound **14** previously protected the carboxylic acid function to obtain derivative **15a,b.** At the same time, compound **14** is coupled with 4-sulfamoylbenzoic acid to achieve derivative **17**. Finally, compounds **15a,b** and **17** are deprotected by hydrolysis with LiOH obtaining the desiderate derivatives **16a,b** and **18** as depicted in Scheme 2.

**Scheme 3.**
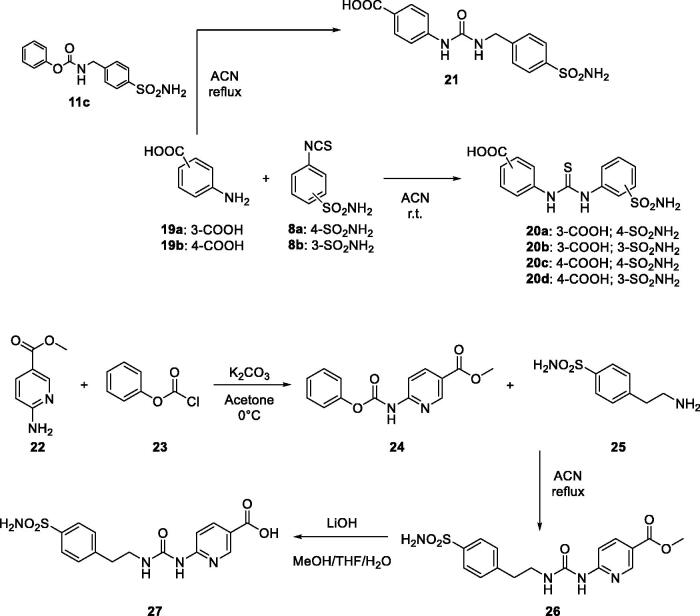
General synthesis of derivatives **20–27.**

In similar manner, thioureido compounds **20a–d** are obtained by coupled aromatic amines **19a,b** with isothiocyanate **8a** and **8b**. In addition, derivative **21** is accomplished by direct aminolysis of carbamates **11c** with compound **19a** as outlined in Scheme 3.

**Scheme 4.**
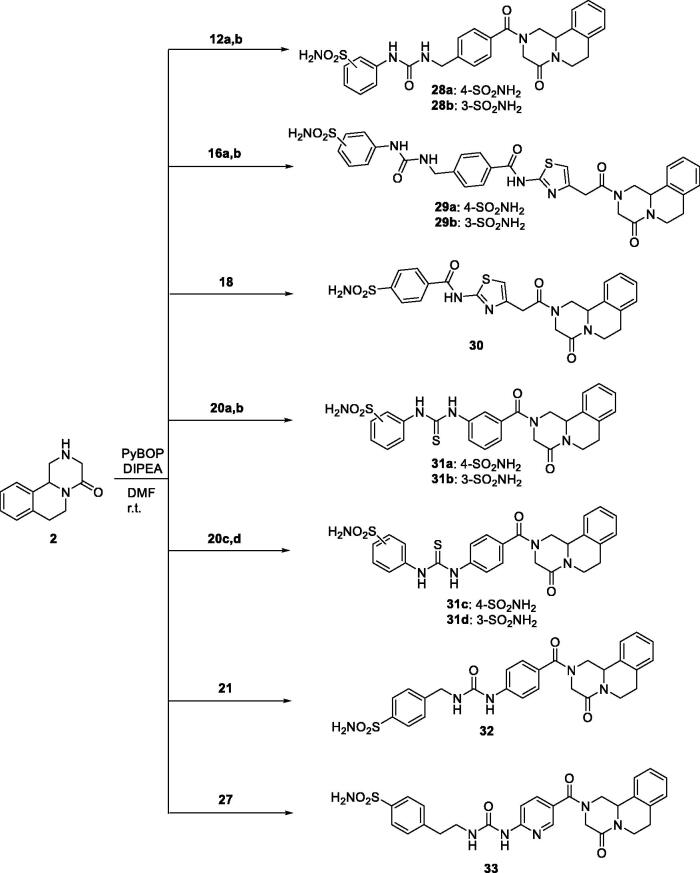
General synthesis of derivatives **28–33**.

We also introduced the pyridine scaffold as part of the linker to obtain compound **27** in good yield. The synthesis is started by reacting the pyridine compound **22** with phenyl carbonochloridate **23** and, followed direct aminolysis of carbamate **24** with aminosulfonamide **25** to achieve the ureido derivative **26**. Finally, the methyl ester has been hydrolysed employing LiOH as mentioned above for the previously derivative (Scheme 3) to obtain compound **27** in excellent yield. These sulphonamide building blocks were then coupled with compound **2** using standard coupling reagents such as PyBOP to obtain the corresponding amides derivatives **28–33** in good yields (Scheme 4).

**Scheme 5.**
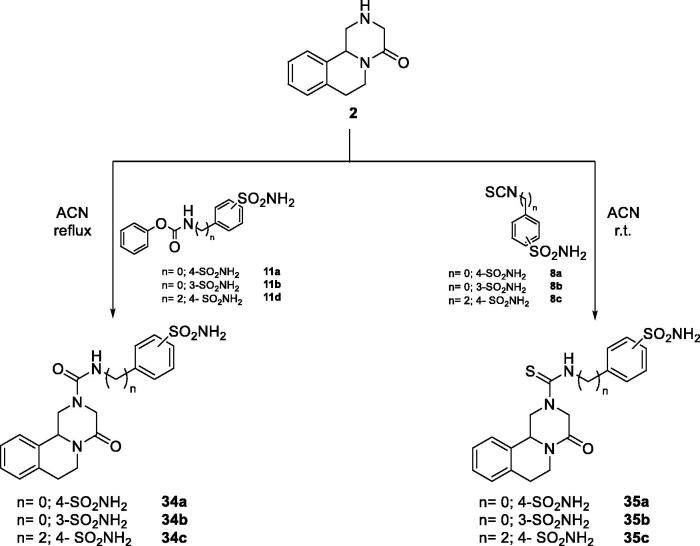
General synthesis of derivatives **34–35**.

So far, we have obtained amide derivatives as PZQ-CAI and, for this reason, we have also explored the possibility of obtaining ureido and thioureido compounds directly coupled with the pyrazino isoquinolinone moiety of PZQ (**2)**. To afford ureido derivatives **34a–c** we employed the mentioned reaction of direct aminolysis of sulphonamide carbamates (**11a,b,d**). On the other hand, thioureido compounds **35a–c** are obtained coupling scaffold **2** with isothiocyanate sulphonamides **8a–c** in excellent yields and reported in Scheme 5.

### 2.2. Carbonic anhydrase inhibition

After synthesis, each compound (**4a–b**, **9**, **28–35)** was evaluated *in vitro* against the physiologically most-relevant hCA isoforms such as hCAs I, II, IV, IX and parasitic isoforms from *S. mansoni* (SmCA), *Trypanosoma cruzi* (TcCA) and *Leishmania donovani* (LdCA) by applying the stopped-flow technique and are compared in Table 1 with the standard sulphonamide inhibitor acetazolamide (**AAZ**).

**Table 1.** Inhibition data of human CA isoforms I, II, IV, IX SmCA, LdCA and TcCA with compounds **4a–b**, **9**, **28–35** and **AAZ** by a stopped flow CO_2_ hydrase assay^23^.

*K_i_* (nM)*							
Cmp	hCA I	hCAII	hCA IV	hCA IX	SmCA	TcCA	LdCA
**4a**	93.8 ± 8.8	44.6 ± 3.4	8863 ± 617	30.3 ± 3.0	1894 ± 195	373.7 ± 33.2	371.4 ± 26.9
**4b**	67.7 ± 5.1	17.5 ± 1.0	784.5 ± 58.3	138.7 ± 8.0	93.6 ± 7.0	476.4 ± 50.1	416.9 ± 33.9
**9**	485.8 ± 24.9	130.6 ± 14.0	966.2 ± 82.8	173.6 ± 17.2	771.0 ± 40.1	93.2 ± 5.8	276.1 ± 22.4
**28a**	74.4 ± 3.9	8.2 ± 0.7	548.1 ± 29.6	31.8 ± 1.8	613.8 ± 50.6	92.3 ± 5.3	339.8 ± 33.9
**28b**	96.5 ± 6.5	14.9 ± 1.5	851.4 ± 56.2	29.9 ± 1.6	824.5 ± 66.8	95.2 ± 9.8	300.3 ± 30.5
**29a**	314.2 ± 30.5	49.6 ± 3.2	540.6 ± 30.3	31.9 ± 1.9	1643 ± 97	598.5 ± 54.7	85.5 ± 6.77
**29b**	506.5 ± 27.9	7.1 ± 0.6	788.7 ± 66.2	199.2 ± 17.6	900.9 ± 77.6	303.1 ± 30.2	217.9 ± 17.9
**30**	58.6 ± 6.2	8.5 ± 0.5	422.4 ± 26.6	119.3 ± 12.4	7.8 ± 0.6	834.2 ± 77.1	81.3 ± 6.4
**31a**	36.6 ± 2.7	6.7 ± 0.6	179.9 ± 19.5	280.2 ± 15.4	8.8 ± 0.8	1647 ± 129.1	248.5 ± 15.9
**31b**	95.2 ± 10.3	41.8 ± 4.4	451.4 ± 24.3	31.2 ± 1.6	2488 ± 144	309.4 ± 33.4	322.8 ± 16.7
**31c**	81.5 ± 5.5	13.7 ± 1.2	8702 ± 559	31.1 ± 2.9	62.8 ± 4.0	588.6 ± 37.0	401.0 ± 20.7
**31d**	464.6 ± 30.1	122.4 ± 12.1	320.2 ± 32.7	137.6 ± 7.8	764.1 ± 68.3	392.7 ± 24.7	82.8 ± 5.5
**32**	80.7 ± 4.8	26.5 ± 2.4	664.7 ± 47.3	111.7 ± 11.3	6353 ± 472	420.3 ± 33.2	400.8 ± 34.8
**33**	128.1 ± 11.9	49.2 ± 3.5	6809 ± 379	90.9 ± 9.6	581.3 ± 32.5	186.5 ± 15.9	87.5 ± 4.9
**34a**	70.4 ± 5.7	8.4 ± 0.8	4412 ± 371	24.7 ± 1.44	59.6 ± 4.3	566.5 ± 44.2	253.0 ± 19.4
**34b**	409.0 ± 23.3	45.6 ± 3.0	516.6 ± 52.4	91.9 ± 8.8	7199 ± 661	765.6 ± 45.8	347.1 ± 34.3
**34c**	20.5 ± 2.0	5.8 ± 0.3	4180 ± 442	101.9 ± 5.9	5.6 ± 0.5	746.9 ± 69.7	362.1 ± 27.3
**35a**	302.8 ± 24.1	53.9 ± 4.4	7113 ± 404	82.2 ± 8.6	79.4 ± 4.2	735.1 ± 57.6	322.9 ± 22.2
**35b**	86.5 ± 9.0	8.0 ± 0.8	47.9 ± 4.1	83.3 ± 7.3	6152 ± 612	706.0 ± 51.5	237.3 ± 12.5
**35c**	64.0 ± 5.4	7.6 ± 0.4	8267 ± 719	97.1 ± 7.9	5.0 ± 0.5	463.1 ± 28.4	484.6 ± 46.0
**PZQ**	>10000	>10000	>10000	>10000	>10000	>10000	>10000
**AAZ**	250.0 ± 22.1	12.1 ± 0.8	74.0 ± 5.6	25.8 ± 2.3	42.5 ± 4.3	61.6 ± 5.8	91.7 ± 5.8

*Mean from three different assays, by a stopped flow technique (errors were in the range of ± 5–10% of the reported values).

As shown in Table 1, we have investigated different PZQ derivatives bearing a sulphonamide moiety in order to understand the key features to obtain potent SmCA inhibitors. The following structure activity relationship (SAR) may be noted regarding the inhibition data.

The linker between the pyrazino isoquinolinone moiety and benzenesulfonamide played a pivotal role for the modulation of potency and selectivity against the different isoforms taken into account. Indeed, an ethyl linker among the two scaffolds such as for compound **4b** showed to be essential to improve the potency than **4a** without it in both hCAs (with the exception of hCA IX) and SmCA. On the other hand, in TcCA and beta LdCA isoforms we did not observe this feature proving non-essential for the potency of inhibition against these two isoforms. Compound **9**, with selenium linker, not improved the potency showing K_i_ in high nanomolar range for all hCAs and SmCA. On the contrary, for the other two parasitic isoforms (TcCA and LdCA) this molecule proved to be more active than compounds **4a–b**, in particular, for TcCA. It is interesting to note how the different position of sulphonamide group changed the potency of inhibition for compounds **29a–b.** When it was placed in *meta* position (**29 b**) showed to be more potent than *para* position (**29a**) against both hCA II and SmCA isoforms. Removing the ureido moiety from the linker of compounds **29a–b** we observed for derivative **30** a substantial increment in inhibitory activity especially for SmCA, which showed a K_i_ in the low nanomolar range (K_i_ 7.8 nM). With regard to compounds **31a–c** the importance of different substituents especially for SmCA was interesting to note. Indeed, **31a**, with a sulphonamide in position 4 and carboxylic linker in position 3, proved to be a potent inhibitor of this isoform with a K_i_ of 8.8 nM. Shifting the sulphonamide group in position 3 (**31b**), on the other hand, resulted in a drastically decrement of the activity against SmCA showing a constant value in the micromolar range (K_i_ 2488 nM). The same features can be observed for compounds **31c** and **31d** with carboxylic linker in position 4 although less marked. The ureido and thioureido derivatives **34a–c** and **35a–c** showed similar characteristics where the spacer between benezenesulfonamide and urea moieties played a pivotal role in the modulation of the activity against SmCA. Indeed, methyl linker (**34b** and **35b**) proved to be the less potent conversely to the other ureido compounds showing the length of this spacer essential for the potency.

From a general point of view, the modulation of SmCA proved to be more sensitive to the different linkers and substitutions moving the activity from nanomolar to micromolar range with minor modifications with respect to hCA II. On the other hand, SmCA belonging to the α-CAs as the human isoforms not showed high selective inhibition by the compounds reported here. In this context, structural studies could be useful to understand these characteristics.

### 2.3. X-ray crystallography

**Figure 2.**
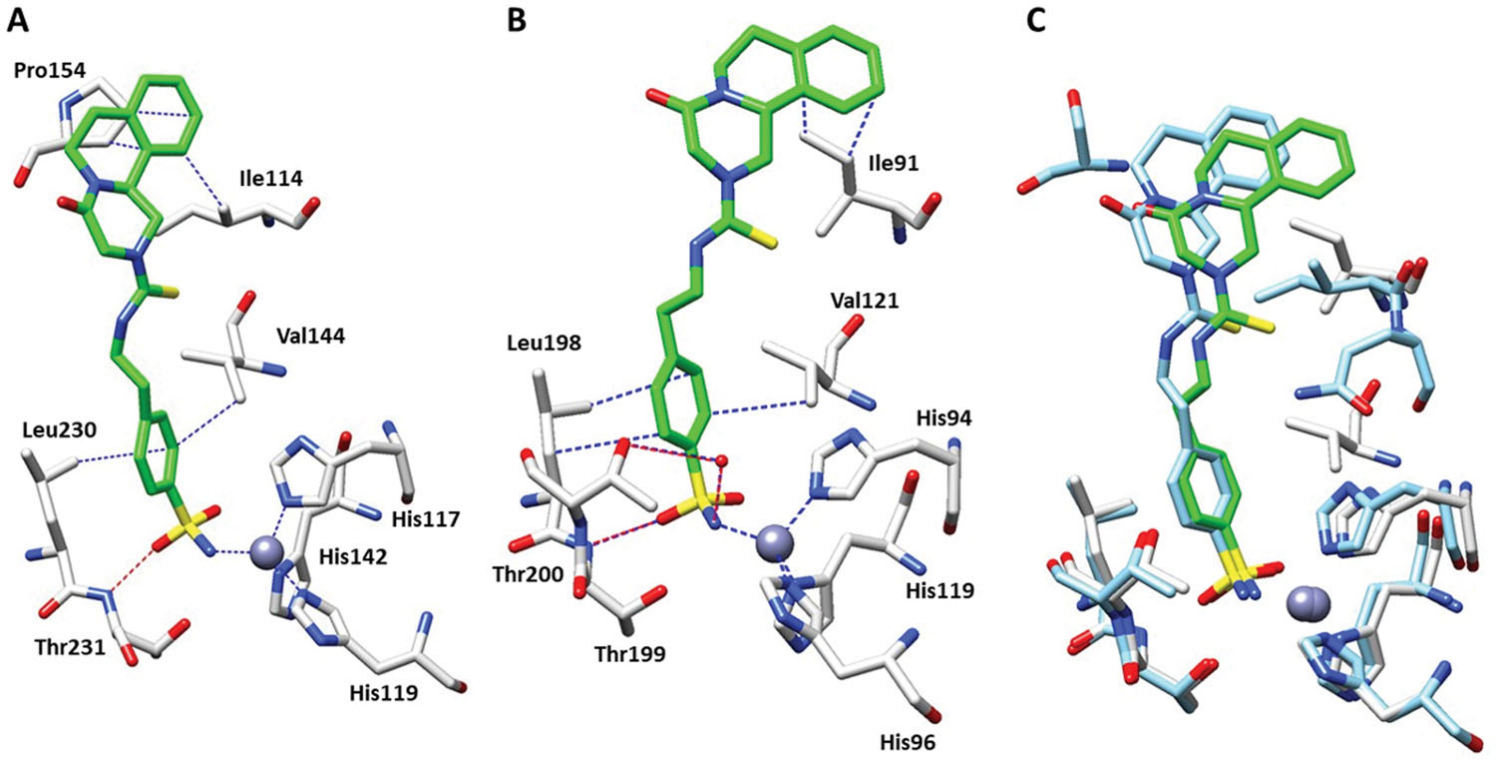
**(A)** X-ray crystal structures of SmCA bound with compound **35c** (PDB: 7YZH). **(B)** X-ray crystal structures of hCA II bound with compound **35c** (PDB: 7R1X). Panel **C** shows their superimposition in the active site (green inhibitor/hCA II, blue inhibitor/SmCA). Residues involved in the binding of inhibitors are also shown; the grey sphere represents the zinc atom in the active site of the proteins.

For structural studies, we sought to complement ligand **35c** with SmCA and hCA II in order to observe possible differences in the active site interactions. Initially, we solved the crystal structure of SmCA in the presence of **35c** at a resolution of 1.79 Å (Figure 2(A)) observing a strong density inside the active site fully compatible with the inhibitor (supplemental Figure S1). The binding mode of **35c** was the classical of this moiety anchoring the sulphonamide group through the catalytic zinc and displacing the water molecule. In addition, compound **35c** formed the characteristic hydrogen bond with residue Thr231, stabilising the interaction. Several hydrophobic interactions are observed for both aromatic ring with side chains Val144 and Leu230 and the terminal portion of inhibitor with Ile114 and Pro154. At the same time, the structure of compound **35c** complexed with hCAII has been solved at a resolution of 1.35 Å (Figure 2(B)). The benzenesulfonamide scaffold showed the same binding mode as mentioned above, with the hydrogen bond between the oxygen of the sulphonamide group and Thr199 stabilising the binding of the inhibitor as in the case of complex with SmCA as typically found for this class of inhibitors^24^. Several hydrophobic interactions were observed also in this complex for both aromatic ring with Val121 and Leu198 and the terminal portion of the tail with Ile91. Unlike the previous complex with SmCA, this time a water bridge is observed among Thr200 and the nitrogen atom of the sulphonamide moiety further stabilising the complex.

**Figure 3.**
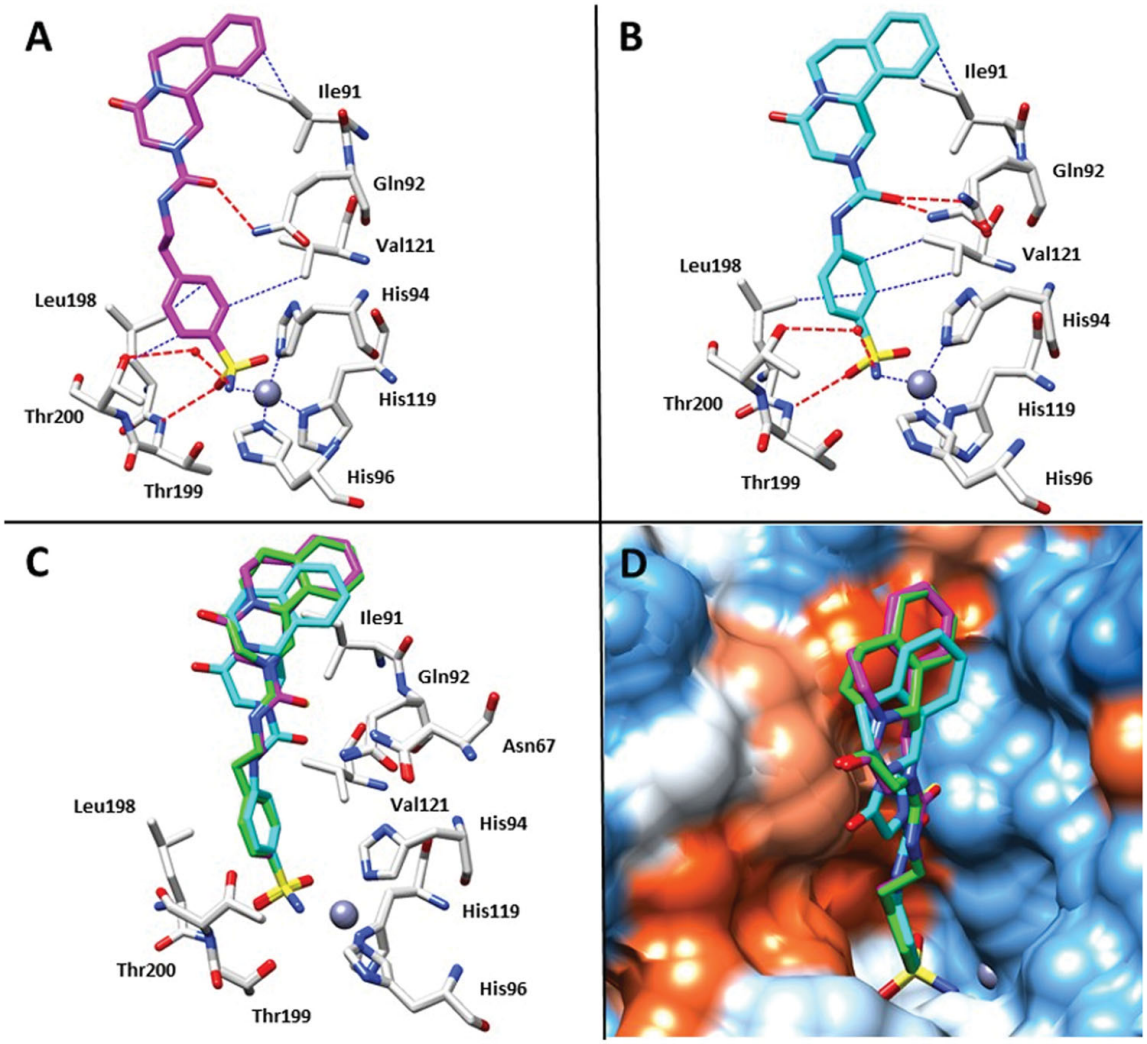
**(A)** X-ray crystal structures of hCA II bound with compound **34c** (PDB: 7QZX). **(B)** X-ray crystal structures of hCA II bound with compound **34a** (PDB: 7YWT). **(C)** Superimposition in the active site of compounds **34a**, **34c** and **35c**; residues involved in the binding of inhibitors are also shown; the grey sphere represents the zinc atom in the active site of the proteins. **(D)** Compounds **34a**, **34c** and **35c** inside the active site of hCA II; Hydrophobic (red) and hydrophilic (blue) residues are labelled.

From a superposition of the two complexes, we found no substantial differences occurring between the two complexes of SmCA and hCA II (Figure 2(C)) explaining the similar potency of action against the two different CAs. To provide additional structural basis of the inhibitory profile of PZQ-CAI, we undertook an X-ray crystallographic study using the cytosolic hCA II. Initially, we consider the bioisosteric substitution of thioureido moiety of **35c** with ureido one of compound **34c**. This complex was solved at a resolution of 1.24 Å (Figure 3(A)) and the maps showed, also this time, a clear electron density inside the active site fully compatible with the inhibitor (supplemental Figure S1). As reported for compound **35c**, the ureido analog **34c** showed the same interaction of the benzenesulfonamide moiety with the amino acids side chains in the active site, such as the hydrogen bond with Thr199 and the hydrophobic interactions with Leu198, Val121 and Ile91. On the other hand, the ureido moiety displayed an additional hydrogen bond with Gln92 which further stabilises the molecule and explains the increased potency against hCA II. The last derivative taken into account was compound **34a** since we aimed to evaluate how the ethylene linker in the ureido moiety could affects the location inside the active site of hCA II (Figure 3(B)). The sulphonamide group showed all the classical connections mentioned above as the hydrogen bond with Thr199 and the hydrophobic interactions with residues of Leu198 and Val121. In addition, as for complex **35c**/hCA II, we observed a water bridge among Thr200 and the nitrogen atom of sulphonamide group. On the other hand, for the inhibitor tail we observed interactions similar to the 34c/hCAII complex, such as the hydrogen bond with Gln92 (in both the alternative conformations introduced for the side chain) and a hydrophobic connection with Ile91.

A superimposition of compounds **34a**, **34c** and **35c** in the hCA II active site showed a similar location in a hydrophobic part of the hCAII active site (Figure 2(C, D)), stabilised mainly by the hydrophobic interaction with Ile91. Removing the ethylene linker for compound **34a** also did not change its arrangement in the catalytic pocket, proving that the key part of these derivatives is the pyrazino isoquinolinone scaffold that stabilise the complexes in a hydrophobic task, explaining, thus, their similar potency against the hCA II isoform.

### 2.4. In vitro *antischistosomal activity against newly transformed schistosomula and adult* S. mansoni

**Figure 4.**
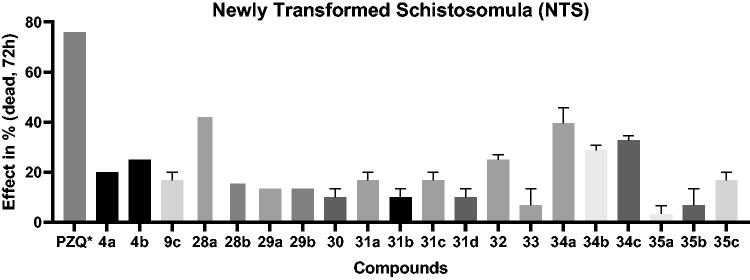
*In vitro* activity of compounds **4a–b**, **9**, **28–35** against NTS at 10 µM for 72 h. Viability of worms is evaluated via microscopy. * PZQ concentration 3.3 µM.

With the aim to test the novel PZQ-CAIs as anti-schistosomiasis agents, all compounds were first evaluated against newly transformed schistosomula (NTS) at an initial concentration of 10 μM to evaluate the activity against these immature worms (Figure 4). Most of the compounds reported here lacked activity (<30%) after 72 h of exposure. Only compounds **28a**, **34a** and **34c** had an activity over 30%, however these compounds do not reach the minimum threshold of activity (>60%).

**Figure 5.**
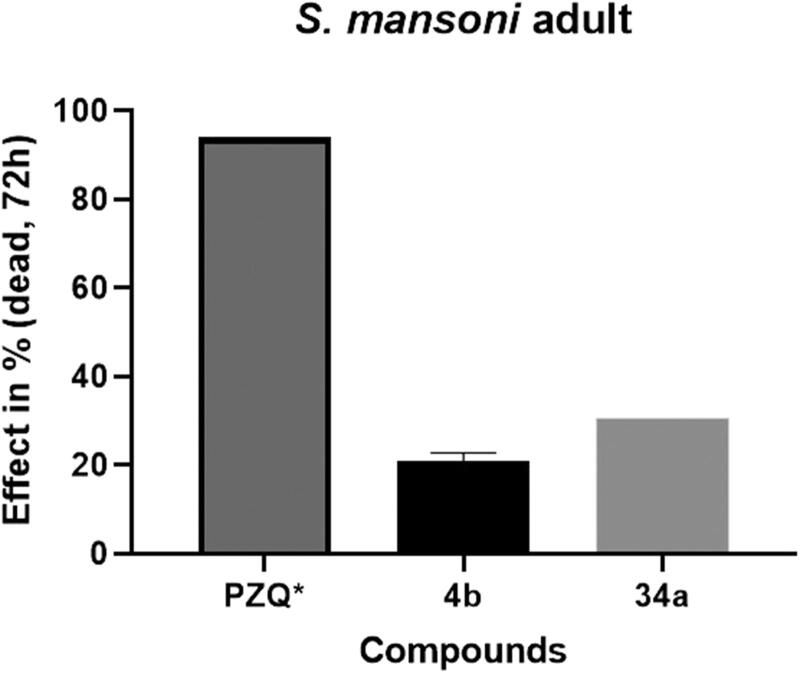
*In vitro* activity of compounds **4b** and **34a** against *S. mansoni* adult worms at 10 µM for 72 h and viability of worms is evaluated via microscopy. * PZQ concentration at 3.3 µM.

Given the broad antiparasitic efficacy of PZQ against different developmental stages of *S. mansoni*^9^^,^^10^^,^^25^, we evaluated whether compounds could be more active against the adult stage selecting one of the most potent compounds against NTS such as **34a** and one ineffective drugs as **4b**. These compounds were subsequently tested on adult *S. mansoni* worms at the same concentration (10 μM) as depicted in Figure 5.

For compound **4b** an activity of 20.9% and for **34a** an activity of 30.6% was observed concluding that these compounds are relatively inactive in this assay.

## 3. Conclusion

In summary, we report on the first PZQ derivatives bearing a primary sulphonamide moiety as novel potential agents against schistosomiasis hence combining, with a multitarget approach, two mechanisms of action targeting ion channels through PZQ and the inhibition of CA from *S. mansoni* (SmCA) offering a potentially better efficacy. In order to found key molecular features required for the affinity towards the SmCA, different linkers were synthetised, discovering potent inhibitors of SmCA such as compounds **30**, **31a**, **34c** and **35c** with a K_i_ in the low nanomolar range (K_i_ 5.0–8.8 nM). In addition, crystallographic analysis of the schistosome tegumental enzyme SmCA and human CA II complexed with a selection of these inhibitors showed the hydrophobic tails of the inhibitors is located in the both isoforms in the same hydrophobic task, explaining the lack of selectivity. Finally, all compounds synthetised in our study were evaluated *in vitro* against the immature *Schistosoma* worms and two of them (**4b** and **34a**) also against to the adult form in order to evaluate the efficacy at this worm stage. Although the compounds tested here for their antischistosomal activity showed low activity the information as obtained in this work should, in the longer term, help in the identification of novel, lead molecules that inhibit SmCA and could become new, clinically useful, anti-schistosome therapeutics. Further optimisation of this novel class is required to synthetise compounds, which exhibit strong activity against *Schistosoma spp*. and therefore enhance the potential of this strategy for treating schistosomiasis.

## 4. Experimental section

### 4.1. General

Anhydrous solvents and all reagents were purchased from Sigma-Aldrich, VWR and TCI. All reactions involving air- or moisture-sensitive compounds were performed under a nitrogen atmosphere. Nuclear magnetic resonance (^1^H NMR, ^13^C NMR and ^77^Se NMR) spectra were recorded using a Bruker Advance III 400 MHz spectrometer in DMSO-*d_6_* or CDCl_3_. Chemical shifts are reported in parts per million (ppm) and the coupling constants (*J*) are expressed in Hertz (Hz). Splitting patterns are designated as follows: s, singlet; d, doublet; t, triplet; m, multiplet; brs, broad singlet; dd, double of doubles. The assignment of exchangeable protons (N*H*) was confirmed by the addition of D_2_O. Analytical thin-layer chromatography (TLC) was carried out on Merck silica gel F-254 plates. Flash chromatography purifications were performed on Merck silica gel 60 (230–400 mesh ASTM) as the stationary phase, and ethyl acetate, *n*-hexane, acetonitrile and methanol were used as eluents. The solvents used in MS measurements were acetone, acetonitrile (Chromasolv grade), purchased from Sigma-Aldrich (Milan, Italy), and mQ water 18 MΩ, obtained from Millipore’s Simplicity system (Milan, Italy). The mass spectra were obtained using a Varian 1200 L triple quadrupole system (Palo Alto, CA, USA) equipped with electrospray source (ESI) operating in both positive and negative ions. Stock solutions of analytes were prepared in acetone at 1.0 mg mL^−1^ and stored at 4 °C. Working solutions of each analyte were freshly prepared by diluting stock solutions in a mixture of mQ H_2_O/ACN 1/1 (*v/v*) up to a concentration of 1.0 μg mL^−1^ The mass spectra of each analyte were acquired by introducing, via syringe pump at 10/L min^−1^, the working solution. Raw data were collected and processed by Varian Workstation, version 6.8, software. All compounds reported here are >95% of purity by NMR spectroscopy.

### 4.2. Chemistry

#### 4.2.1. Synthesis of 1,2,3,6,7,11b-hexahydro-4H-pyrazino[2,1-a]isoquinolin-4-one (2)

Praziquantel (**1,** 10 gr, 32.01 mmol) was dissolved in 3 M HCl (50 mL) and the mixture was stirred at reflux overnight. The solution is cooled at room temperature, quenched with saturated KHCO_3_ solution and extracted with DCM. The crude reaction was purified by flash chromatography (MeOH/DCM 10:90) to afford a yellow solid. Yield 60%. **^1^H NMR** (400 MHz, CDCl_3_) δ(ppm): 7.32–7.17 (4H, m), 4.91 (1H, ddd, *J* = 12.52, 4.92, 2.52 Hz), 4.85 (1H, dd, *J* = 9.90, 4.40 Hz), 3.78 (1H, dd, *J* = 13.09, 4.14 Hz), 3.71 (1H, d, *J* = 17.46 Hz), 3.57 (1H, d, *J* = 17.33 Hz), 2.97–2.77 (4H, m), 2.28 (1H, bs); **^13^C NMR** (100 MHz, CDCl_3_) δ(ppm): 167.5, 135.2, 134.5, 129.7, 127.3, 126.9, 124.9, 57.1, 50.2, 50.0, 39.1, 29.1; MS (ESI positive) *m/z*: 203.1 [M + H]^+^

#### 4.2.2. General synthesis of compounds 4a–b

To a solution of 1,2,3,6,7,11 b-hexahydro-4H-pyrazino[2,1-a]isoquinolin-4-one (**2**, 100 mg, 0.494 mmol, 1.eq), HATU (244 mg, 0.643 mmol, 1.3 eq), and appropriate carboxylic acid (**3a,b**, 1eq.) in dry DMF (3 mL) was added under ice cooling and an inert atmosphere DIPEA(128 mg, 0.988 mmol, 2 eq). The yellow solution was stirred overnight at room temperature. The reaction mixture was quenched with ice-cooled, saturated NH_4_Cl solution and stirred for 15 min to give a precipitate, which was collected by vacuum filtration and washed with water. The obtained solid was triturated with Et_2_O to yield the derivatives **4a,b**.

#### 4.2.3. 4–(4-Oxo-1,3,4,6,7,11b-hexahydro-2H-pyrazino[2,1-a]isoquinoline-2-carbonyl) benzenesulfonamide (4a)

Following the general procedure, carboxylic acid **3a** (99 mg, 0.494 mmol) gave the product as a white solid **4a**, yield 88%. **^1^H NMR** (400 MHz, DMSO-*d6*) δ(ppm): 7.97 (2H, d, *J* = 8.06 Hz), 7.77–7.75 (2H, m), 7.53 (2H, bs), 7.43–7.27 (4H, m), 5.14 (1H, m), 5.03 (1H, m), 4.61 (2H,m), 4.04 (2H, m), 2.93–2.84 (3H, m); **^13 ^C NMR** (100 MHz, DMSO-*d6*) δ(ppm): 168.7, 164.4, 146.3, 138.9, 136.0, 133.7, 130.0, 128.8, 128.0, 127.6, 126.8, 126.2, 48.6, 46.8, 39.2, 29.0, 26.8; MS (ESI positive) *m/z*: 386.1 [M + H]^+^

#### 4.2.4. 4–(3-Oxo-3–(4-oxo-1,3,4,6,7,11b-hexahydro-2H-pyrazino[2,1-a]isoquinolin-2-yl)propyl) benzenesulfonamide (4b)

Following the general procedure, carboxylic acid **3b** (113 mg, 0.494 mmol) gave the product as a white solid **4b**, yield 38%. **^1^H NMR** (400 MHz, DMSO-*d6*) δ(ppm): 7.77 (2H, d, *J* = 7.89 Hz), 7.50 (3H, t, *J* = 7.59 Hz), 7.31–7.26 (6H, m), 4.97 (1H, d, *J* = 7.07 Hz), 4.84 (1H, m), 4.58–4.38 (2H, m), 4.08 (1H, d, *J* = 17.28 Hz), 3.80 (1H, d, *J* = 17.69 Hz), 2.98–2.83 (7H, m); **^13 ^C NMR** (100 MHz, DMSO-*d6*) δ(ppm): 170.9, 165.5, 146.6, 142.7, 135.9, 134.1, 129.9, 127.9, 127.4, 126.9, 126.5 126.3, 55.3, 49.0, 46.6, 45.3, 39.1, 33.9, 31.0, 29.1; MS (ESI positive) *m/z*: 414.2 [M + H]^+^

#### 4.2.5. Synthesis of 2–(4-(chloromethyl)benzoyl)-1,2,3,6,7,11b-hexahydro-4H-pyrazino[2,1-a]isoquinolin-4-one (5)

To a solution of 1,2,3,6,7,11 b-hexahydro-4H-pyrazino[2,1-a]isoquinolin-4-one (**2**, 500 mg, 2.472 mmol, 1.eq), 4-(chloromethyl)benzoyl chloride (467 mg, 2.472 mmol, 1 eq), in dry DCM (10 ml) was added under ice cooling and an inert atmosphere Et_3_N (300 mg, 2.966 mmol, 1.2 eq.). The reaction mixture was quenched with ice-cooled, saturated NH_4_Cl solution and extracted with DCM. The combined organic phase was dried over Na_2_SO_4_, filtrated, and evaporated. The crude material was purified by flash chromatography (1:1 hexane/ethyl acetate) to yield **5** as white solid (yield 76%). **^1^H NMR** (400 MHz, DMSO-*d6*) δ(ppm): 7.60 (4H, aps), 7.26 (4H, m), 5.07 (2H, m), 4.88 (2H, s), 4.60 (2H, m), 4.15–4.04 (2H, m), 2.94–2.83 (3H, m); **^13 ^C NMR** (100 MHz, DMSO-*d6*) δ(ppm):169.5, 164.8, 140.6, 135.9, 135.6, 133.9, 129.9, 129.8, 128.5, 127.9, 127.5, 126.1, 54.9, 46.4. 39.2, 29,0; MS (ESI positive) *m/z*: 355.1 [M + H]^+^

#### 4.2.6. Synthesis of 2–(4-(((4-aminophenyl)selanyl)methyl)benzoyl)-1,2,3,6,7,11b-hexahydro-4H-pyrazino[2,1-a]isoquinolin-4-one (7)

A suspension of 4-selenocyanatoaniline (**6**, 444 mg, 2.254 mmol, 1 eq) in EtOH (10 mL) was treated with NaBH_4_ (341 mg, 9.016 mmol, 4 eq) and maintained under stirring for 30 min. The resulting solution was treated with 2–(4-(chloromethyl)benzoyl)-1,2,3,6,7,11b-hexahydro-4H-pyrazino[2,1-a]isoquinolin-4-one (**5**, 800 mg, 2.254 mmol, 1 eq) and the mixture was stirred at room temperature for additional 3 h. Afterwards, the reaction was quenched with saturated NH_4_Cl and then EtOAc was added. The organic phase was collected and the aqueous phase was extracted with EtOAc. The combined organic phases were dried over Na_2_SO_4_ and concentrated under reduced pressure. The crude material was purified by flash column chromatography (MeOH/DCM: 5:95), to yield **7** as a white solid (yield 97%). **^1^H NMR** (400 MHz, DMSO-*d6*) δ(ppm): 7.40 (3H, m), 7.27–7.23 (5H, m), 7.11 (2H, d, *J* = 7.30 Hz), 6.49 (2H, d, *J* = 8.41 Hz), 5.29 (2H, bs), 5.05 (2H, m), 4.59 (2H, m), 4.24–4.12 (2H, m), 4.05 (2H, s), 2.92–2.84 (3H, m); **^13 ^C NMR** (100 MHz, DMSO-*d6*) δ(ppm): 169.8, 164.3, 149.7, 143.0, 137.1, 135.9, 133.6, 130.0, 129.6, 128.1, 128.0, 127.7, 127.5, 115.4, 113.3, 54.2, 46.7, 43.4, 39.2, 32.8, 29.0; **^77^Se NMR** (76 MHz, DMSO-*d6*) δ(ppm): 370.9; MS (ESI positive) *m/z*: 492.1 [M + H]^+^

#### 4.2.7. Synthesis of 4–(3-(4-((4–(4-oxo-1,3,4,6,7,11b-hexahydro-2H-pyrazino[2,1-a]isoquinoline-2-carbonyl)benzyl)selanyl)phenyl)thioureido)benzenesulfonamide (9)

A mixture of isothiocyanate **8a** (44 mg, 0.204 mmol, 1 eq) and 2–(4-(((4-aminophenyl)selanyl)methyl)benzoyl)-1,2,3,6,7,11b-hexahydro-4H-pyrazino[2,1-a]isoquinolin-4-one (**7,** 100 mg, 0.204 mmol, 1 eq) in acetonitrile (4 mL) was stirred at room temperature overnight. Then water was added, and the precipitate was filtered off and dried under vacuum to afford compound **12** as with solid (yield 50%).**^1^H NMR** (400 MHz, DMSO-*d6*) δ(ppm): 10.14 (1H, bs), 10.09 (1H, bs), 7.79 (2H, d, *J* = 8.70 Hz), 7.71 (2H, d, *J* = 8.71 Hz), 7.48–7.40 (8H, m), 7.32 (2H, bs), 7.26 (3H, m), 5.05 (2H, m), 4.59 (2H, m), 4.31 (2H, s), 4.06 (2H, m), 2.91–2.83 (3H, m); **^13 ^C NMR** (100 MHz, DMSO-*d6*) δ(ppm): 180.3, 169.7, 163.5, 143.5, 142.2, 140.0, 139.4, 136.0, 134.0, 133.7, 130.0, 129.8, 129.6, 128.3, 128.0, 127.5, 127.1, 126.0, 125.8, 125.0, 123.5, 54.9, 46.2, 43.4, 39.2, 31.4, 29.0; **^77^Se NMR** (76 MHz, DMSO-*d6*) δ(ppm): 386.3; MS (ESI positive) *m/z*: 706.1 [M + H]^+^

#### 4.2.8. General synthesis of compounds 12a–b

A mixture of appropriate carbamate (**11a–b**) (1 eq) and 4-(aminomethyl)benzoic acid (**10**) (1 eq) in acetonitrile (10 mL) was stirred at reflux overnight. Then water was added, and the precipitate was filtered off and dried under vacuum.

#### 4.2.9. 4-((3–(4-Sulfamoylphenyl)ureido)methyl)benzoic acid (12a)

Following the general procedure, carbamate **11a** (500 mg, 1.710 mmol) gave the product as a white solid **12a**, yield 80%. **^1^H NMR** (400 MHz, DMSO-*d6*) δ(ppm): 9.14 (1H, bs), 7.95 (2H, d, *J* = 8.15 Hz), 7.71 (2H, d, *J* = 8.78 Hz), 7.59 (2H, d, *J* = 8.82 Hz), 7.45 (2H, d, *J* = 8.15 Hz), 7.20 (2H, bs), 6.94 (1H, t, *J* = 5.91 Hz), 4.42 (2H, d, *J* = 5.68 Hz); **^13 ^C NMR** (100 MHz, DMSO-*d6*) δ(ppm): 168.3, 155.9, 146.9, 144.6, 137.2, 130.5, 130.3, 128.1, 127.8, 118.0, 43.6; MS (ESI positive) *m/z*: 350.1 [M + H]^+^

#### 4.2.10. 4-((3–(3-Sulfamoylphenyl)ureido)methyl)benzoic acid (12 b)

Following the general procedure, carbamate **11 b** (500 mg, 1.710 mmol) gave the product as a white solid **12 b**, yield 75%. **^1^H NMR** (400 MHz, DMSO-*d6*) δ(ppm): 12.87 (1H, bs), 9.18 (1H, bs), 8.06 (1H, s), 7.95 (2H, d, *J* = 8.02 Hz), 7.57 (1H, d, *J* = 7.81 Hz), 7.45 (2H, d, *J* = 8.12 Hz), 7.42–7.32 (2H, m), 7.32(2H, bs), 6.95 (1H, t, *J* = 5.61 Hz), 4.42 (2H, d, *J* = 5.31 Hz); **^13 ^C NMR** (100 MHz, DMSO-*d6*) δ(ppm): 168.2, 156.1, 146.5, 145.5, 141.9, 130.4, 130.2, 128.0, 121.4, 119.1, 115.5; MS (ESI positive) *m/z*: 350.1 [M + H]^+^

#### 4.2.11. Synthesis of methyl 2–(2-aminothiazol-4-yl)acetate (14)

Methanol (10 mL) was cooled to 0 °C and thionyl chloride (2 eq) was added dropwise. Then 2–(2-aminothia-zol-4-yl)acetic acid (**13**, 500 mg, 3.16 mmol, 1 eq) was added and reaction mixture stirred under reflux for 1.5 h. The solvent was evaporated and the oily residue triturated with diethyl ether. White precipitate was filtered off and dried.

#### 4.2.12. General synthesis of compounds 15a–b

To a solution of methyl 2–(2-aminothiazol-4-yl)acetate (**14**, 200 mg, 1.161 mmol, 1.eq), HATU (574 mg, 1.509 mmol, 1.3 eq), and appropriate carboxylic acid (**12a,b**, 1eq.) in dry DMF (4 mL) was added under ice cooling and an inert atmosphere DIPEA(2.322 mmol, 2 eq.). The yellow solution was stirred overnight at room temperature. The reaction mixture was quenched with ice-cooled, saturated NH_4_Cl solution and stirred for 15 min to give a precipitate, which was collected by vacuum filtration and washed with water. The obtained solid was triturated with Et_2_O to yield the derivatives **15a,b**.

#### 4.2.13. Methyl 2–(2-(4-((3–(4-sulfamoylphenyl)ureido)methyl)benzamido)thiazol-4-yl)acetate (15a)

Following the general procedure, carboxylic acid **12a** (405 mg, 1.161 mmol) gave the product as a white solid **15a**, yield 80%. **^1^H NMR** (400 MHz, DMSO-*d6*) δ(ppm): 12.67 (1H, bs), 9.14 (1H, bs), 8.10 (2H, d, *J* = 8.29 Hz), 7.72 (2H, d, *J* = 8.81 Hz), 7.60 (2H, d, *J* = 8.86 Hz), 7.48 (2H, d, *J* = 8.26 Hz), 7.19 (2H, bs), 7.10 (1H, s), 6.96 (1H, t, *J* = 5.81 Hz), 4.43 (2H, d, *J* = 5.54 Hz), 3.79 (2H, s), 3.66 (3H, s); **^13 ^C NMR** (100 MHz, DMSO-*d6*) δ(ppm):170.5, 165.8, 155.9, 152.1, 144.5, 137.2, 130.4, 129.8, 129.2, 128.0, 127.7, 121.7 117.9, 111.9, 52.7, 43.5, 37.4; MS (ESI positive) *m/z*: 504.1 [M + H]^+^

#### 4.2.14. Methyl 2–(2-(4-((3–(3-sulfamoylphenyl)ureido)methyl)benzamido)thiazol-4-yl)acetate (15 b)

Following the general procedure, carboxylic acid **12b** (405 mg, 1.161 mmol) gave the product as a white solid **15b**, yield 47%. **^1^H NMR** (400 MHz, DMSO-*d6*) δ(ppm): 12.69 (1H, bs), 9.07 (1H, bs), 8.10 (2H, d, *J* = 8.12 Hz), 8.06 (1H, s), 7.58 (2H, d, *J* = 8.13 Hz), 7.48 (2H, d, *J* = 8.22 Hz), 7.44 (1H, d, *J* = 7.89 Hz), 7.34 (2H, bs), 7.10 (1H, s), 6.87 (1H, t, *J* = 5.88 Hz), 4.43 (2H, d, *J* = 5.63 Hz), 3.80 (2H, s), 3.66 (3H, s); **^13^C NMR** (100 MHz, DMSO-*d6*) δ(ppm): 171.5, 165.8, 159.3, 156.1, 146.3, 145.5, 141.8, 131.4, 130.2, 129.2, 128.0, 121.5, 119.2, 115.6, 111.9, 52.7, 43.5, 37.4; MS (ESI positive) *m/z*: 504.1 [M + H]^+^

#### 4.2.15. General synthesis of compounds 16a–b

To a solution of appropriate ester (**15a,b** 1.eq), was added to a solution of THF/H_2_O/MeOH (1 ml:0.5 ml:1 ml) and at room temperature was added LiOH (4 eq). The mixture was stirred overnight and then quenched with 3 N HCl. After 15 min to give a precipitate, which was collected by vacuum filtration and washed with water. The obtained compounds were triturated with Et_2_O to yield the derivatives **16a,b**.

#### 4.2.16. 2–(2-(4-((3–(4-Sulfamoylphenyl)ureido)methyl)benzamido)thiazol-4-yl)acetic acid (16a)

Following the general procedure, starting from the ester **15a** (300 mg, 0.595 mmol), the product was a white solid **16a**, yield 95%. **^1^H NMR** (400 MHz, DMSO-*d6*) δ(ppm): 12.62 (2H, bs), 9.11 (1H, bs), 8.11 (2H, d, *J* = 8.11 Hz), 7.72 (2H, d, *J* = 8.73 Hz), 7.60 (2H, d, *J* = 8.74 Hz), 7.49 (2H, d, *J* = 8.20 Hz), 7.19 (2H, bs), 7.06 (1H, s), 6.94 (1H, bs), 4.44 (2H, d, *J* = 5.40 Hz), 3.68 (2H, s); **^13 ^C NMR** (100 MHz, DMSO-*d6*) δ(ppm): 172.5, 165.7, 159.1, 155.8, 146.0, 144.4, 137.1, 131.4, 130.3, 129.1, 128.0, 127.6, 117.9, 111.5, 43.4, 37.8; MS (ESI positive) *m/z*: 490.1 [M + H]^+^

#### 4.2.17. 2–(2-(4-((3–(3-Sulfamoylphenyl)ureido)methyl)benzamido)thiazol-4-yl)acetic acid (16 b)

Following the general procedure, starting from the ester **15b** (200 mg, 0.397 mmol), the product was a white solid **16b**, yield 98%. **^1^H NMR** (400 MHz, DMSO-*d6*) δ(ppm): 12.66 (1H, bs), 9.16 (1H, bs), 8.11 (2H, d, *J* = 7.92 Hz), 8.07 (1H, s), 7.58 (1H, d, *J* = 7.56 Hz), 7.49 (2H, d, *J* = 8.00 Hz), 7.45–7.38 (2H, m), 7.34 (2H, bs), 7.06 (1H, s), 6.95 (1H, bs), 4.43 (2H, s), 3.69 (2H, s); **^13 ^C NMR** (100 MHz, DMSO-*d6*) δ(ppm): 172.5, 165.7, 159.1, 156.1, 146.2, 145.5, 145.2, 141.8, 131.4, 130.1, 129.1, 127.9, 121.5, 119.1, 115.6, 111.5, 43.5, 37.8; MS (ESI positive) *m/z*: 490.1 [M + H]^+^

#### 4.2.18. Synthesis of methyl 2–(2-(4-sulfamoylbenzamido)thiazol-4-yl)acetate (17)

To a solution of methyl 2–(2-aminothiazol-4-yl)acetate (**14**, 500 mg, 2.903 mmol, 1.eq), HATU (3.773 mmol, 1.3 eq), and 4-sulfamoylbenzoic acid (2.903 mmol, 1eq.) in dry DMF (4 ml) was added under ice cooling and an inert atmosphere DIPEA (5.806 mmol, 2 eq.). The yellow solution was stirred overnight at room temperature. The reaction mixture was quenched with ice-cooled, saturated NH_4_Cl solution and stirred for 15 min to give a precipitate, which was collected by vacuum filtration and washed with water. The obtained solid was triturated with Et_2_O to yield the compound **17** (80%). **^1^H NMR** (400 MHz, DMSO-*d6*) δ(ppm): 12.92 (1H, bs), 8.26 (2H, d, *J* = 8.32 Hz), 7.98 (2H, d, *J* = 8.31 Hz), 7.57 (2H, bs), 7.14 (1H, s), 3.81 (2H, s), 3.66 (3H, s); **^13^C NMR** (100 MHz, DMSO-*d6*) δ(ppm): 171.4, 165.1, 159.1, 148.2, 144.7, 135.9, 129.8, 126.7, 112.2, 52.7, 37.4; MS (ESI positive) *m/z*: 354.0 [M + H]^+^

#### 4.2.19. Synthesis of 2–(2-(4-sulfamoylbenzamido)thiazol-4-yl)acetic acid (18)

Methyl 2–(2-(4-sulfamoylbenzamido)thiazol-4-yl)acetate (**17**, 350 mg, 0.975 mmol, 1 eq.) was added to a solution of THF/H_2_O/MeOH (1 ml:0.5 ml:1 mL), at room temperature was added LiOH (3.9 mmol, 4 eq). The mixture was stirred overnight and then quenched with 3 N HCl. After 15 min to give a precipitate, which was collected by vacuum filtration and washed with water. The obtained solid was triturated with Et_2_O to yield the compound **17** (90%). **^1^H NMR** (400 MHz, DMSO-*d6*) δ(ppm): 12.90 (1H, bs), 8.26 (2H, d, *J* = 7.98 Hz), 7.98 (2H, d, *J* = 8.01 Hz), 7.59 (2H, bs), 7.10 (1H, s), 3.70 (2H, s); **^13 ^C NMR** (100 MHz, DMSO-*d6*) δ(ppm): 172.51, 165.2, 159.1, 148.1, 145.2, 136.1, 129.8, 126.7, 111.9, 37.7; MS (ESI positive) *m/z*: 342.0 [M + H]^+^

#### 4.2.20. General synthesis of compounds 20a–d

A mixture of appropriate isothiocyanate (**8a*–*b**) (0.934 mmol, 1 eq) and benzoic acid (**19a,b**) (0.934 mmol, 1 eq) in acetonitrile (10 mL) was stirred at room temperature overnight. Then water was added, and the precipitate was filtered off and dried under vacuum.

#### 4.2.21. 3–(3-(4-Sulfamoylphenyl)thioureido)benzoic acid (20a)

Following the general procedure, the product was a white solid **20a**, yield 85%. **^1^H NMR** (400 MHz, DMSO-*d6*) δ(ppm): 13.05 (1H, bs), 10.22 (2H, bs), 8.13 (1H, s), 7.82–7.22 (6H, m), 7.53–7.49 (1H, m), 7.34 (2H, bs); **^13^C NMR** (100 MHz, DMSO-*d6*) δ(ppm): 180.8, 167.9, 143.4, 140.5, 140.2, 132.0, 129.7, 128.9, 127.1, 126.4, 125.4, 123.7; MS (ESI positive) *m/z*: 352.0 [M + H]^+^

#### 4.2.22. 3–(3-(3-Sulfamoylphenyl)thioureido)benzoic acid (20b)

Following the general procedure, the product was a white solid **20b**, yield 81%. **^1^H NMR** (400 MHz, DMSO-*d6*) δ(ppm): 13.04 (1H, bs), 10.15 (2H, bs), 8.11 (1H, s), 8.01 (1H, s), 7.76 (3H, m), 7.58–7.51 (3H, m), 7.43 (2H, bs); **^13^C NMR** (100 MHz, DMSO-*d6*) δ(ppm): 181.0, 167.9, 145.2, 140.8, 140.5, 132.1, 129.9, 129.7, 128.9, 127.8, 126.3, 125.4, 122.4, 121.5; MS (ESI positive) *m/z*: 352.0 [M + H]^+^

#### 4.2.23. 4–(3-(4-Sulfamoylphenyl)thioureido)benzoic acid (20c)

Following the general procedure, the product was a white solid **20c**, yield 87%. **^1^H NMR** (400 MHz, DMSO-*d6*) δ(ppm): 12.82 (1H, bs), 10.37 (1H, bs), 10.34 (1H, bs), 7.95 (2H, d, *J* = 8.64 Hz), 7.82 (2H, d, *J* = 8.73 Hz), 7.75–7.70 (4H, m), 7.35 (2H, bs); **^13 ^C NMR** (100 MHz, DMSO-*d6*) δ(ppm): 180.3, 167.8, 144.4, 143.3, 140.3, 130.8, 127.1, 127.0, 123.7, 123.0; MS (ESI positive) *m/z*: 352.0 [M + H]^+^

#### 4.2.24. 4–(3-(3-Sulfamoylphenyl)thioureido)benzoic acid (20d)

Following the general procedure, the product was a white solid **20d**, yield 85%. **^1^H NMR** (400 MHz, DMSO-*d6*) δ(ppm): 12.81 (1H, bs), 10.30 (1H, bs), 10.27 (1H, bs), 8.03 (1H, s), 7.96 (2H, d, *J* = 8.31 Hz), 7.77 (1H, d, *J* = 7.65 Hz), 7.70 (2H, d, *J* = 8.31 Hz), 7.64 (1H, d, *J* = 7.62 Hz), 7.57 (1H, t, *J* = 7.73 Hz), 7.45 (2H, bs); **^13^C NMR** (100 MHz, DMSO-*d6*) δ(ppm):180.6, 167.9, 145.3, 144.5, 140.8, 130.9, 130.0, 127.8, 126.9, 123.0, 122.6, 121.5; MS (ESI positive) *m/z*: 352.0 [M + H]^+^

#### 4.2.25. Synthesis of 4–(3-(4-sulfamoylbenzyl)ureido)benzoic acid (21)

A mixture of carbamate **11c** (0.653 mmol, 1 eq) and 4-aminobenzoic acid (**19b**) (0.653 mmol, 1 eq) in acetonitrile (6 ml) was stirred at reflux overnight. Then water was added, and the precipitate was filtered off and dried under vacuum to obtain a white solid with yield of 81% **^1^H NMR** (400 MHz, DMSO-*d6*) δ(ppm): 12.56 (1H, bs), 9.11 (1H, bs), 7.85 (2H, d, *J* = 8.77 Hz), 7.83 (2H, d, *J* = 8.40 Hz), 7.55 (2H, d, *J* = 8.51 Hz), 7.51 (2H, d, *J* = 8.06 Hz), 7.35 (2H, bs), 6.93 (1H, t, *J* = 5.30 Hz), 4.42 (2H, d, *J* = 5.56 Hz); **^13 ^C NMR** (100 MHz, DMSO-*d6*) δ(ppm): 168.3, 156.2, 145.9, 145.5, 143.6, 131.6, 128.5, 126.8, 123.9, 117.8, 43.4; MS (ESI positive) *m/z*: 350.1 [M + H]^+^

#### 4.2.26. Synthesis of methyl 6-((phenoxycarbonyl)amino)nicotinate (24)

A mixture of phenyl chloroformiate (**23**) (3.193 mmol, 1 eq), methyl 6-aminonicotinate (**22**) (3.193 mmol, 1 eq) and anhydrous potassium carbonate (4.151 mmol, 1.3 eq.) in acetone (10 ml) was stirred at 0 °C for 3 h. Then the solvent was removed under vacuum and water was added. The precipitate was filtered off and dried under vacuum to obtain a white solid with yield of 76% **^1^H NMR** (400 MHz, CDCl_3_) δ(ppm): 9.25 (1H, bs), 8.50 (1H, s), 7.72 (1H, s), 7.43 (3H, m), 7.26 (4H, m), 4.00 (3H, s); **^13 ^C NMR** (100 MHz, CDCl_3_) δ(ppm): 165.1, 154.3, 150.8, 150.6, 140.1, 129.8, 126.7, 125.7, 121.4, 121.1, 120.5, 52.8; MS (ESI positive) *m/z*: 273.1 [M + H]^+^

#### 4.2.27. Synthesis of methyl 6–(3-(4-sulfamoylphenethyl)ureido)nicotinate (26)

A mixture of methyl 6-((phenoxycarbonyl)amino)nicotinate (**24**) (1.468 mmol, 1 eq) and 4–(2-aminoethyl)benzenesulfonamide (**25**) (1.468 mmol, 1 eq) in acetonitrile (8 ml) was stirred at reflux overnight. Then water was added, and the precipitate was filtered off and dried under vacuum to obtain a white solid with yield of 50%**^1^H NMR** (400 MHz, DMSO-*d6*) δ(ppm): 9.71 (1H, bs), 8.72 (1H, s), 8.19 (1H, m), 8.17 (1H, s), 7.80 (2H, d, *J* = 8.12 Hz), 7.57 (1H, d, *J* = 8.73 Hz), 7.49 (2H, d *J* = 8.00 Hz), 7.34 (2H, bs), 3.87 (3H, s), 3.49 (2H, m), 2.92 (2H, t, *J* = 6.54 Hz); **^13^C NMR** (100 MHz, DMSO-*d6*) δ(ppm): 165.9, 157.3, 155.0, 149.9, 144.5, 143.1, 139.7, 130.1, 126.6, 119.4, 111.8, 52.9, 41.2, 36.1; MS (ESI positive) *m/z*: 379.1 [M + H]^+^

#### 4.2.28. Synthesis of 6–(3-(4-sulfamoylphenethyl)ureido)nicotinic acid (27)

Methyl 6–(3-(4-sulfamoylphenethyl)ureido)nicotinate (**26**, 0.793 mmol, 1 eq.) was added to a solution of THF/H_2_O/MeOH (1 ml:0.5 ml:1 ml), at room temperature and was added LiOH (3.171 mmol, 4 eq). The mixture was stirred overnight and then quenched with 3 N HCl. After 15 min to give a precipitate, which was collected by vacuum filtration and washed with water. The obtained solid was triturated with Et_2_O to yield the compound **27** (69%).**^1^H NMR** (400 MHz, DMSO-*d6*) δ(ppm): 13.04 (1H, bs), 9.68 (1H, s), 8.71 (1H, s), 8.17 (1H, m), 8.08 (1H, m), 7.80 (2H, d, *J* = 7.79 Hz), 7.54 (3H, m), 7.33 (2H, bs), 3.50 (3H, m), 2.92 (2H, m); **^13 ^C NMR** (100 MHz, DMSO-*d6*) δ(ppm): 167.0. 157.1, 155.1, 150.0, 144.6, 143.1, 140.0, 130.2, 126.7, 120.5, 111.8, 41.3, 36.2; MS (ESI positive) *m/z*: 365.1 [M + H]^+^

#### 4.2.29. General synthesis of compounds 28–33

To a solution of 1,2,3,6,7,11 b-hexahydro-4H-pyrazino[2,1-a]isoquinolin-4-one (**2**, 0.494 mmol, 1.eq), PyBOP (0.642 mmol, 1.3 eq), and appropriate carboxylic acid (**12a–b**, **16a–b**, **18**, **20a–d**, **21** or **27**, 0.494 mmol, 1eq.) in dry DMF (3 ml) was added under ice cooling and an inert atmosphere DIPEA(2 eq.). The solution was stirred overnight at room temperature. The reaction mixture was quenched with ice-cooled, saturated NH_4_Cl solution and stirred for 15 min to give a precipitate, which was collected by vacuum filtration and washed with water. The obtained solid was triturated with Et_2_O to yield the derivatives **28–33**.

#### 4.2.30. 4–(3-(4–(4-Oxo-1,3,4,6,7,11b-hexahydro-2H-pyrazino[2,1-a]isoquinoline-2-carbonyl) benzyl)ureido)benzenesulfonamide (28a)

Following the general procedure, the product was a white solid **28a**, yield 83%. **^1^H NMR** (400 MHz, DMSO-*d6*) δ(ppm): 9.08 (1H, bs), 7.72 (2H, d, *J* = 8.78 Hz), 7.61 (2H, d, *J* = 8.79 Hz), 7.54 (2H, m), 7.47 (2H, d, *J* = 7.81 Hz), 7.26 (4H, m), 7.19 (2H, bs), 6.90 (1H, t, *J* = 5.72 Hz), 5.08 (1H, m), 4.58 (1H, m), 4.43 (2H, d, *J* = 5.35 Hz), 3.05 (2H, m), 2.95–2.77 (3H, m); **^13^C NMR** (100 MHz, DMSO-*d6*) δ(ppm):175.5, 169.9, 166.8, 155.9, 144.5, 143.5, 137.1, 136.1, 134.2, 130.1, 128.4, 127.7, 127.6, 126.2, 122.2, 117.9, 54.5, 46.8, 43.5, 39.2, 29.1, 26.9; MS (ESI positive) *m/z*: 534.2 [M + H]^+^

#### 4.2.31. 3–(3-(4–(4-Oxo-1,3,4,6,7,11b-hexahydro-2H-pyrazino[2,1-a]isoquinoline-2-carbonyl) benzyl)ureido)benzenesulfonamide (28 b)

Following the general procedure, the product was a white solid **28b**, yield 79%. **^1^H NMR** (400 MHz, DMSO-*d6*) δ(ppm): 9.05 (1H, bs), 8.08 (1H, s), 7.60–7.55 (3H, m), 7.48–7.45 (3H, m), 7.43–7.40 (2H, m), 7.34 (2H, bs), 7.26 (3H, m), 6.84 (1H, t, *J* = 5.58 Hz), 5.08 (1H, m), 4.59 (1H, m), 4.44 (2H, d, *J* = 5.22 Hz), 4.13 (2H, m), 3.05 (2H, m), 2.92–2.77 (3H, m); **^13 ^C NMR** (100 MHz, DMSO-*d6*) δ(ppm): 169.9, 164.8, 156.0, 145.5, 143.6, 141.8, 136.0, 134.2, 130.1, 130.0, 128.3, 128.0, 127.6, 126.1, 125.4, 121.5, 119.1, 115.6, 54.4, 46.7, 43.5, 39.2, 29.1, 26.8; MS (ESI positive) *m/z*: 534.2 [M + H]^+^

#### 4.2.32. N-(4–(2-oxo-2–(4-oxo-1,3,4,6,7,11b-hexahydro-2H-pyrazino[2,1-a]isoquinolin-2-yl)ethyl) thiazol-2-yl)-4-((3–(4-sulfamoylphenyl)ureido)methyl)benzamide (29a)

Following the general procedure, the product was a white solid **29a**, yield 81%. **^1^H NMR** (400 MHz, DMSO-*d6*) δ(ppm): 12.65 (1H, bs), 9.14 (1H, bs), 8.09 (2H, d, *J* = 6.91 Hz), 7.73 (2H, d, *J* = 8.26 Hz), 7.62 (2H, d, *J* = 8.21 Hz), 7.49 (2H, d, *J* = 6.67 Hz), 7.31–7.17 (7H, m), 6.96 (1H, bs), 4.87 (1H, m), 4.72–4.56 (3H, m), 4.44 (2H, d, *J* = 3.60 Hz), 4.15 (1H, m), 3.90 (2H, s), 3.79 (1H, d, *J* = 17.85 Hz), 2.84–2.81 (3H, m); **^13^C NMR** (100 MHz, DMSO-*d6*) δ(ppm): 168.6, 168.4, 165.4, 164.9, 155.4, 145.6, 144.0, 136.7, 135.5, 133.5, 129.6, 128.8, 127.6, 127.2, 127.0, 126.3, 117.5, 110.9, 55.2 46.3, 43.0, 38.5, 37.0, 28.7, 26.4; MS (ESI positive) *m/z*: 674.2 [M + H]^+^

#### 4.2.33. N-(4–(2-oxo-2–(4-oxo-1,3,4,6,7,11b-hexahydro-2H-pyrazino[2,1-a]isoquinolin-2-yl)ethyl) thiazol-2-yl)-4-((3–(3-sulfamoylphenyl)ureido)methyl)benzamide (29 b)

Following the general procedure, the product was a white solid **29b**, yield 81%. **^1^H NMR** (400 MHz, DMSO-*d6*) δ(ppm): 12.65 (1H, bs), 9.07 (1H, bs), 8.09–8.06 (3H, m), 7.57 (1H, d, *J* = 7.76 Hz), 7.49–7.24 (10H, m), 7.17 (1H, s), 6.87 (1H, bs), 4.68–4.54 (3H, m), 4.43 (2H, d, *J* = 4.44 Hz), 4.15 (1H, m), 3.89 (2H, s), 3.78 (1H, d, *J* = 18.04 Hz), 2.93–2.77 (3H, m); **^13^C NMR** (100 MHz, DMSO-*d6*) δ(ppm): 169.9, 169.0 164.9, 156.0, 146.2, 145.5, 143.6, 141.8, 136.0, 134.2, 134.0, 130.1, 130.0, 129.9, 129.1, 128.3, 128.0, 127.9, 127.6, 126.7, 126.1, 121.5, 119.1, 115.6, 55.6, 46.8, 46.7, 43.5, 39.2, 29.1, 26.8; MS (ESI positive) *m/z*: 674.2 [M + H]^+^

#### 4.2.34. N-(4–(2-oxo-2–(4-oxo-1,3,4,6,7,11b-hexahydro-2H-pyrazino[2,1-a]isoquinolin-2-yl)ethyl)thiazol-2-yl)-4-sulfamoylbenzamide (30)

Following the general procedure, the product was a white solid **30**, yield 75%. **^1^H NMR** (400 MHz, DMSO-*d6*) δ(ppm): 12.92 (1H, bs), 8.25 (2H, bs), 7.98 (2H, bs) 7.58 (2H, bs), 7.37–7.07 (6H, m), 4.87 (1H, m), 4.71–4.51 (3H, m), 4.15 (1H, d, *J* = 17.21 Hz), 3.90 (2H, s), 3.78 (1H, d, *J* = 17.18 Hz), 2.81 (3H, m); **^13^C NMR** (100 MHz, DMSO-*d6*) δ(ppm):168.7, 165.3, 164.8, 148.1, 135.9, 134.3, 133.9, 130.0, 129.8, 127.9, 127.6, 127.5, 126.6, 126.3, 111.6, 55.6, 49.7, 46.7, 38.9, 29.1, 26.8; MS (ESI positive) *m/z*: 526.1 [M + H]^+^

#### 4.2.35. 4–(3-(3–(4-Oxo-1,3,4,6,7,11b-hexahydro-2H-pyrazino[2,1-a]isoquinoline-2-carbonyl) phenyl)ureido)benzenesulfonamide (31a)

Following the general procedure, the product was a white solid **31a**, yield 71%. **^1^H NMR** (400 MHz, DMSO-*d6*) δ(ppm): 10.25 (1H, bs), 10.21 (1H, s), 7.81 (2H, d, *J* = 8.51 Hz), 7.73 (2H, d, *J* = 8.54 Hz), 7.60 (1H, d, *J* = 7.79 Hz), 7.53 (1H, m), 7.33 (4H, m), 7.25 (3H, m), 7.00 (1H, m), 5.06 (1H, m), 4.59 (2H, m), 4.20 (2H, m), 2.96–2.84 (3H, m); **^13^C NMR** (100 MHz, DMSO-*d6*) δ(ppm): 180.9, 169.5, 164.9, 143.4, 140.2, 140.1, 135.9, 135.8, 133.9, 129.9, 128.3, 128.0, 127.5, 127.1, 126.4, 126.2, 124.6, 123.6, 54.5, 39.2, 31.6, 29.0; MS (ESI positive) *m/z*: 520.2 [M + H]^+^

#### 4.2.36. 3–(3-(3–(4-Oxo-1,3,4,6,7,11b-hexahydro-2H-pyrazino[2,1-a]isoquinoline-2-carbonyl) phenyl)ureido)benzenesulfonamide (31b)

Following the general procedure, the product was a white solid **31b**, yield 63%. **^1^H NMR** (400 MHz, DMSO-*d6*) δ(ppm): 10.19 (1H, bs), 10.15 (1H, s), 8.00 (1H, s), 7.76 (2H, d, *J* = 7.61 Hz), 7.75–7.53 (4H, m), 7.42 (2H, bs), 7.35 (1H, m), 7.25 (3H, m), 6.98 (1H, m), 5.06 (1H, m), 4.60 (2H, m), 4.36–3.99 (2H, m), 2.96–2.80 (3H, m); **^13^C NMR** (100 MHz, DMSO-*d6*) δ(ppm): 180.9, 169.5, 164.9, 143.4, 140.2, 140.1, 135.9, 135.8, 133.9, 129.9, 128.3, 128.0, 127.5, 127.1, 126.4, 126.2, 124.6, 123.6, 54.5, 39.2, 31.6, 29.0; MS (ESI positive) *m/z*: 520.2 [M + H]^+^

#### 4.2.37. 4–(3-(4–(4-Oxo-1,3,4,6,7,11b-hexahydro-2H-pyrazino[2,1-a]isoquinoline-2-carbonyl) phenyl)ureido)benzenesulfonamide (31c)

Following the general procedure, the product was a white solid **31a**, yield 88%. **^1^H NMR** (400 MHz, DMSO-*d6*) δ(ppm): 10.30 (1H, bs), 10.28 (1H, s), 7.82 (2H, d., *J* = 8.48 Hz), 7.75 (2H, d, *J* = 8.61 Hz), 7.69 (2H, d, *J* = 8.11 Hz), 7.57 (2H, d, *J* = 7.26 Hz), 7.35 (2H, bs), 7.27 (4H, m), 5.09 (1H, m), 4.59 (1H, m), 4.16 (2H, m), 3.05 (2H, m), 2.93–2.84 (3H, m); **^13^C NMR** (100 MHz, DMSO-*d6*) δ(ppm): 180.3, 169.7, 164.9, 143.5, 142.2, 142.1, 140.2, 137.4, 136.1, 131.3, 130.1, 129.0, 128.1, 127.6, 127.2, 126.2, 123.5, 54.5, 39.3, 29.1, 19.0, 17.7; MS (ESI positive) *m/z*: 520.2 [M + H]^+^

#### 4.2.38. 3–(3-(4–(4-Oxo-1,3,4,6,7,11b-hexahydro-2H-pyrazino[2,1-a]isoquinoline-2-carbonyl) phenyl)ureido)benzenesulfonamide (31d)

Following the general procedure, the product was a white solid **31b**, yield 59%. **^1^H NMR** (400 MHz, DMSO-*d6*) δ(ppm): 10.24 (1H, bs), 10.23 (1H, s), 8.03 (1H, s), 7.78 (1H, d, *J* = 7.65 Hz), 7.68 (2H, d, *J* = 7.81 Hz), 7.64–7.57 (5H, m), 7.44 (2H, bs), 7.27 (3H, m), 5.09 (1H, m), 4.59 (1H, m), 4.14 (2H, m), 3.05 (2H, m), 2.93–2.84 (3H, m); **^13^C NMR** (100 MHz, DMSO-*d6*) δ(ppm): 180.7, 169.6, 164.9, 145.2, 141.9, 140.8, 135.9, 134.0, 131.3, 130.0, 128.9, 128.0, 127.8, 127.6, 126.2, 123.6, 122.4, 121.5, 54.5, 46.7, 39.2, 29.1, 26.8; MS (ESI positive) *m/z*: 520.2 [M + H]^+^

#### 4.2.39. 4-((3–(4-(4-Oxo-1,3,4,6,7,11b-hexahydro-2H-pyrazino[2,1-a]isoquinoline-2-carbonyl) phenyl)ureido)methyl)benzenesulfonamide (32)

Following the general procedure, the product was a white solid **32**, yield 55%. **^1^H NMR** (400 MHz, DMSO-*d6*) δ(ppm): 9.02 (1H, bs), 7.83 (2H, d, *J* = 8.10 Hz), 7.57 (2H, d, *J* = 8.36 Hz), 7.52 (2H, d, *J* = 8.06 Hz), 7.48 (2H, d, *J* = 8.14 Hz), 7.35 (2H, bs), 7.26 (4H, m), 6.90 (1H, t, *J* = 5.72 Hz), 5.06 (1H, m), 4.59 (1H, m), 4.43 (2H, d, *J* = 5.60 Hz), 4.08 (2H, m), 3.05 (2H, m), 2.93–2.77 (3H, m); **^13^C NMR** (100 MHz, DMSO-*d6*) δ(ppm): 170.0, 165.0, 156.0, 145.4, 143.5, 143.4, 136.0, 130.0, 129.6, 128.3 128.0, 127.6, 126.7, 117.9, 55.0, 46.8, 43.3, 39.2, 29.1, 26.9; MS (ESI positive) *m/z*: 534.2 [M + H]^+^

#### 4.2.40. 4–(2-(3–(5-(4-Oxo-1,3,4,6,7,11b-hexahydro-2H-pyrazino[2,1-a]isoquinoline-2-carbonyl) pyridin-2-yl)ureido)ethyl)benzenesulfonamide (33)

Following the general procedure, the product was a white solid **33**, yield 70%. **^1^H NMR** (400 MHz, DMSO-*d6*) δ(ppm): 9.55 (1H, bs), 8.35 (1H, bs), 8.06 (1H, bs), 7.90 (1H, bs), 7.80 (2H, bs), 7.50 (3H, m), 7.34–7.27 (6H, m), 5.08 (1H, m), 4.59 (1H, m), 4.39–4.02 (3H, m), 3.50 (2H, s), 2.92–2.84 (5H, m); **^13^C NMR** (100 MHz, DMSO-*d6*) δ(ppm): 167.5, 164.4, 155.1, 154.9, 154.8, 146.9, 144.1, 142.6, 138.2, 135.5, 129.7, 129.5, 127.5, 127.1, 126.2, 125.9, 125.7, 111.2, 53.7, 48.4, 45.9, 41.41, 38.7, 35.8, 28.6; MS (ESI positive) *m/z*: 549.2 [M + H]^+^

#### 4.2.41. General synthesis of compounds 34a-c

A mixture of appropriate carbamate (**11a,b,d**, 0.494 mmol, 1 eq) and 1,2,3,6,7,11 b-hexahydro-4H-pyrazino[2,1-a]isoquinolin-4-one (**2**) (0.494 mmol, 1 eq) in acetonitrile (6 mL) was stirred at reflux overnight. Then water was added, and the precipitate was filtered off and dried under vacuum.

#### 4.2.42. 4-Oxo-N-(4-sulfamoylphenyl)-1,3,4,6,7,11b-hexahydro-2H-pyrazino[2,1-a]isoquinoline-2-carboxamide (34a)

Following the general procedure, the product was a white solid **34a**, yield 58%. **^1^H NMR** (400 MHz, DMSO-*d6*) δ(ppm): 9.12 (1H, bs), 7.76 (2H, d, *J* = 8.79 Hz), 7.72 (2H, d, *J* = 8.80 Hz), 7.46 (2H, d, *J* = 7.46 Hz), 7.36–7.25 (5H, m), 5.01 (1H, dd, *J* = 10.28, 3.45 Hz), 4.73 (1H, dd, *J* = 13.11, 3.18 Hz), 4.61 (1H, d, *J* = 11.40 Hz), 4.50 (1H, d, *J* = 17.34 Hz), 4.00 (1H, d, *J* = 17.34 Hz), 3.21–3.15 (1H, m), 2.94–2.84 (3H, m); **^13^C NMR** (100 MHz, DMSO-*d6*) δ(ppm):165.4, 154.8, 144.3, 136.0, 134.4, 130.0, 128.0, 127.5, 127.3, 126.6, 119.9, 55.2, 48.6, 47.9, 39.1, 29.2; MS (ESI positive) *m/z*: 401.1 [M + H]^+^

#### 4.2.43. 4-Oxo-N-(3-sulfamoylphenyl)-1,3,4,6,7,11b-hexahydro-2H-pyrazino[2,1-a]isoquinoline-2-carboxamide (34 b)

Following the general procedure, the product was a white solid **34b**, yield 53%. **^1^H NMR** (400 MHz, DMSO-*d6*) δ(ppm): 9.11 (1H, bs), 8.10 (1H, s), 7.77 (1H, d, *J* = 7.11 Hz), 7.50–7.45 (3H, m), 7.36 (2H, bs), 7.33–7.26 (3H, m), 5.01 (1H, dd, *J* = 10.15, 2.87 Hz), 4.74 (1H, d, *J* = 10.38 Hz), 4.62 (1H, d, *J* = 11.24 Hz), 3.98 (1H, d, *J* = 17.35 Hz), 3.21–3.15 (1H, m), 2.94–2.84 (3H, m); **^13^C NMR** (100 MHz, DMSO-*d6*) δ(ppm): 165.5, 154.9, 145.3, 141.5, 136.0, 134.4, 130.2, 130.0, 128.0, 127.5, 126.5, 123.4, 120.1, 117.6, 55.2, 48.5, 47.9, 39.1, 29.1; MS (ESI positive) *m/z*: 401.1 [M + H]^+^

#### 4.2.44. 4-Oxo-N-(4-sulfamoylphenethyl)-1,3,4,6,7,11b-hexahydro-2H-pyrazino[2,1-a]isoquinoline-2-carboxamide (34c)

Following the general procedure, the product was a white solid **34c**, yield 90%. **^1^H NMR** (400 MHz, DMSO-*d6*) δ(ppm): 7.78 (2H, d, *J* = 7.95 Hz), 7.44 (2H, d, *J* = 8.03 Hz), 7.39 (1H, d, *J* = 7.45 Hz), 7.33 (2H, bs), 7.30–7.23 (3H, m), 7.01 (1H, t, *J* = 4.95 Hz), 4.86 (1H, dd, *J* = 10.14, 3.49 Hz), 4.61–4.35 (2H, m), 4.33 (1H, d, *J* = 17.40 Hz), 3.77 (1H, d, *J* = 17.41 Hz), 3.35–3.33 (2H, m), 2.99 (1H, m), 2.96–2.81 (5H, m); **^13^C NMR** (100 MHz, DMSO-*d6*) δ(ppm): 165.8, 157.4, 144.9, 142.9, 135.9, 134.6, 130.1, 129.9, 127.9, 127.4, 126.6, 126.5, 55.2, 48.4, 47.6, 42.4, 39.0, 36.5, 29.2; MS (ESI positive) *m/z*: 429.1 [M + H]^+^

#### 4.2.45. General synthesis of compounds 35a–c

A mixture of appropriate isothiocyanate (**8a–c**) (0.494 mmol, 1 eq) and 1,2,3,6,7,11 b-hexahydro-4H-pyrazino[2,1-a]isoquinolin-4-one (**2**) (0.494 mmol, 1 eq) in acetonitrile (5 ml) was stirred at room temperature overnight. Then water was added, and the precipitate was filtered off and dried under vacuum.

#### 4.2.46. 4-Oxo-N-(4-sulfamoylphenyl)-1,3,4,6,7,11b-hexahydro-2H-pyrazino[2,1-a]isoquinoline-2-carbothioamide (35a)

Following the general procedure, the product was a white solid **35a**, yield 84%. **^1^H NMR** (400 MHz, DMSO-*d6*) δ(ppm): 9.78 (1H, bs), 7.80 (2H, bs), 7.58 (2H, bs), 7.44 (1H, bs), 7.35–7.30 (5H, m), 5.23 (1H, bs), 5.11 (1H, bs), 4.87 (1H, d, *J* = 16.84 Hz), 4.59 (1H, bs), 4.40 (1H, d, *J* = 17.28 Hz), 3.44 (1H, m), 2.95–2.87 (3H, m); **^13^C NMR** (100 MHz, DMSO-*d6*) δ(ppm):181.3, 164.9, 144.6, 140.6, 136.1, 133.7, 130.0, 128.1, 127.6, 126.8, 126.7, 125.9, 54.3, 52.6, 52.3, 39.1, 29.1; MS (ESI positive) *m/z*: 417.1 [M + H]^+^

#### 4.2.47. 4-Oxo-N-(3-sulfamoylphenyl)-1,3,4,6,7,11b-hexahydro-2H-pyrazino[2,1-a]isoquinoline-2-carbothioamide (35 b)

Following the general procedure, the product was a white solid **35 b**, yield 89%. **^1^H NMR** (400 MHz, DMSO-*d6*) δ(ppm): 9.78 (1H, bs), 7.85 (1H, s), 7.65 (2H, t, *J* = 8.28 Hz), 7.55 (1H, d, *J* = 7.84 Hz), 7.46–7.43 (3H, m), 7.37–7.28 (3H, m), 5.24 (1H, d, *J* = 12.00 Hz), 5.11 (1H, dd, *J* = 10.54, 3.08 Hz), 4.90 (1H, d, *J* = 17.41 Hz), 4.61 (1H, m), 4.39 (1H, d, *J* = 17.41 Hz), 3.46–3.43 (1H, m), 2.93–2.87 (3H, m); **^13^C NMR** (100 MHz, DMSO-*d6*) δ(ppm): 181.5, 164.9, 144.9, 141.9, 136.1, 133.8, 130.0, 129.9, 129.5, 128.1, 127.6, 126.8, 123.4, 122.7, 54.3, 52.6, 52.2, 39.1, 29.1; MS (ESI positive) *m/z*: 417.1 [M + H]^+^

#### 4.2.48. 4-Oxo-N-(4-sulfamoylphenethyl)-1,3,4,6,7,11b-hexahydro-2H-pyrazino[2,1-a]isoquinoline-2-carbothioamide (35c)

Following the general procedure, the product was a white solid **35c**, yield 78%. **^1^H NMR** (400 MHz, DMSO-*d6*) δ(ppm): 8.11 (1H, bs), 7.80 (2H, d, *J* = 7.94 Hz), 7.46 (2H, d, *J* = 7.70 Hz), 7.39–7.28 (6H, m), 5.09 (1H, d, *J* = 10.60 Hz), 4.98 (1H, d, *J* = 6.53 Hz), 4.74 (1H, d, *J* = 17.50 Hz), 4.57 (1H, d, *J* = 8.52 Hz), 4.22 (1H, d, *J* = 17.27 Hz), 3.78 (2H, m), 3.24 (1H, t, *J* = 11.81 Hz), 3.01 (2H, m), 2.91–2.84 (3H, m); **^13^C NMR** (100 MHz, DMSO-*d6*) δ(ppm): 181.4, 165.2, 155.1, 144.6, 143.0, 136.1, 133.9, 130.1, 130.0, 128.1, 127.6, 126.7, 54.4, 51.8, 51.6, 47.4, 39.0, 35.3, 29.1; MS (ESI positive) *m/z*: 445.1 [M + H]^+^

### 4.3. Carbonic anhydrase inhibition

An Applied Photophysics stopped-flow instrument was used to assay the CA catalysed CO_2_ hydration activity^23^. Phenol red (at a concentration of 0.2 mM) was used as an indicator, working at the absorbance maximum of 557 nm, with 20 mM Hepes (pH 7.4) as a buffer, and 20 mM Na_2_SO_4_ (to maintain constant ionic strength), following the initial rates of the CA-catalysed CO_2_ hydration reaction for a period of 10–100 s. The CO_2_ concentrations ranged from 1.7 to 17 mM for the determination of the kinetic parameters and inhibition constants^15^. Enzyme concentrations ranged between 5 and 12 nM. For each inhibitor, at least six traces of the initial 5–10% of the reaction were used to determine the initial velocity. The uncatalyzed rates were determined in the same manner and subtracted from the total observed rates. Stock solutions of the inhibitor (0.1 mM) were prepared in distilled–deionized water and dilutions up to 0.01 nM were done thereafter with the assay buffer. Inhibitor and enzyme solutions were preincubated together for 15 min at room temperature prior to the assay, to allow for the formation of the E–I complex. The inhibition constants were obtained by non-linear least-squares methods using PRISM 3 and the Cheng-Prusoff equation as reported earlier and represent the mean from at least three different determinations. All CA isoforms were recombinant proteins obtained in house, as reported earlier^14^^,^^26–29^.

### 4.4. Crystallisation and X-ray data collection

The SmCA enzyme, purified in recombinant form, as described previously^14^, was crystallised at 296 K using the sitting-drop vapour-diffusion method in 96-well plates (CrystalQuick, Greiner Bio-One, Germany). Drops were prepared using 1 µL protein solution mixed with 1 µL reservoir solution and were equilibrated against 100 µL precipitant solution. The concentration of the protein was 10 mg mL^−1^ in 50 mM Tris pH 8.3. Initial crystallisation conditions were found using the JCSG-plus screen (Molecular Dimensions) and were optimised. Crystal of the complex with **35c** was obtained using 20% PEG 6000, 0.1 M citrate pH 5.0. The crystals belonged to the primitive trigonal space group P3_2_21. The complexes were prepared soaking the SmCA native crystals in the mother liquor solution containing the inhibitors at concentration of 10 mM for two days. Crystals of hCAII were obtained using the hanging drop vapour diffusion method using 24 well Linbro plate. 2 µL of 10 mg/mL solution of hCA II in Tris-HCl 20 mM pH 8.0 were mixed with 2 µL of a solution of 1.5 M sodium citrate, 0.1 M Tris pH 8.0 and were equilibrated against the same solution at 296 K. The complexes were prepared by soaking the hCA II native crystals in the mother liquor solution containing the inhibitors at concentration of 10 mM for two days. All crystals were flash-frozen at 100 K using a solution obtained by adding 15% (v/v) glycerol to the mother liquor solution as cryoprotectant. Data on crystals of the complexes were collected using synchrotron radiation at the XRD2 beamline at Elettra Synchrotron (Trieste, Italy) with a wavelength of 1.000 Å and a DECTRIS Pilatus 6 M detector. Data were integrated and scaled using the program XDS^30^. Data processing statistics are shown in supporting information.

### 4.5. Structure determination

The crystal structure of hCA II (PDB accession code: 4FIK) and SmCA (PDB accession code: 6QQM) without solvent molecules and other heteroatoms was used to obtain initial phases using Refmac5^31^. 5% of the unique reflections were selected randomly and excluded from the refinement data set for the purpose of Rfree calculations. The initial |Fo - Fc| difference electron density maps unambiguously showed the inhibitor molecules. The inhibitor was introduced in the model with 1.0 occupancy. Refinements proceeded using normal protocols of positional, isotropic atomic displacement parameters alternating with manual building of the models using COOT^32^. The quality of the final models were assessed with COOT and RAMPAGE^33^. Crystal parameters and refinement data are summarised in Electronic Supplementary Information (ESI). Atomic coordinates were deposited in the Protein Data Bank (PDB accession code: 7YZH, 7YWT, 7QZX, 7R1X). Graphical representations were generated with Chimaera^34^.

### 4.6. Antischistosomal assays

*In vitro* studies were carried out in accordance with Swiss national and cantonal regulations on animal welfare under the permission number 2070. The drug sensitivity assays with *S. mansoni* (adult and newly transformed schistosomules (NTS)) were carried out as described recently^35^. Briefly, to obtain NTS, cercariae were collected from infected *Biomphalaria glabrata* snails and were mechanically transformed. Adult *S. mansoni* worms were collected by dissecting the mesenteric veins of infected mice at day 49 postinfection. Approximately 30–40 NTS were incubated with the respective test drug in 250 µL of M199 medium (Gibco, Waltham, MA) supplemented with 5% (v/v) foetal calf serum (FCS) (Bioconcept AG, Switzerland), 1% (v/v) penicillin/streptomycin solution (Sigma–Aldrich, Switzerland) and 1% (v/v) antibacterial/antifungal solution for up to 72 h at 37 °C and 5% CO_2_. The drug was tested at 10 µM in triplicate and repeated once. Three adult *S. mansoni* were incubated in a final volume of 2 mL rpmI 1640 supplemented with 5% (v/v) FCS and 1% (v/v) penicillin/streptomycin at 37 °C and 5% CO_2_ for 72 h at 10 µM. The experiment was conducted in duplicate. Worms were judged via microscopic readout 72 h after incubation; they were scored according to phenotypic reference points such as motility, morphology and granularity (scores from 0 to 3).

## Supplementary Material

Supplemental Material
