# Supplementary material for: Development of Praziquantel sulphonamide derivatives as antischistosomal drugs
Source: J Enzyme Inhib Med Chem. 2022 May 29;37(1):1479–94. doi: 10.1080/14756366.2022.2078970 (PMC9154761; doi:10.1080/14756366.2022.2078970)
Supplement: Supplemental Material [file IENZ_A_2078970_SM5784.pdf]

Supplementary Material for

Development of Praziquantel sulfonamide derivatives as antischistosomal drugs

Andrea Angeli [a,b]*, Marta Ferraroni[e], Fabrizio Carta [a], Cécile Häberli[c,d], Jennifer Keiser[c,d], Gabriele Costantino[b], Claudiu T. Supuran [a]

[a] *NEUROFARBA Department, Sezione di Scienze Farmaceutiche, University of Florence, Via Ugo Schiff 6, 50019 Sesto Fiorentino, Florence, Italy.*

[b] *Department of Food and Drug, University of Parma, Parco Area delle Scienze, 27/A, 43124 Parma, Italy.*

[c] *Department of Medical Parasitology and Infection Biology, Swiss Tropical Institute, Socinstrasse 57, CH-4002 Basel, Switzerland; University of Basel, CH-4003 Basel, Switzerland.*

[d] *University of Basel, CH-4003 Basel, Switzerland.*

[e] *Dipartimento di Chimica " Ugo Schiff", University of Florence, Via della Lastruccia 3, 50019 Sesto Fiorentino, Florence, Italy.*

Index

^{1}H, ^{13}C, ^{77}Se Spectra of compounds *S2-41*

Summary of Data Collection and Atomic Model Refinement Statistics for hCAII *S42*

Summary of Data Collection and Atomic Model Refinement Statistics for SmCA *S43*

Figure S1 *S44*

Copies of NMR Spectrum of synthesized compounds

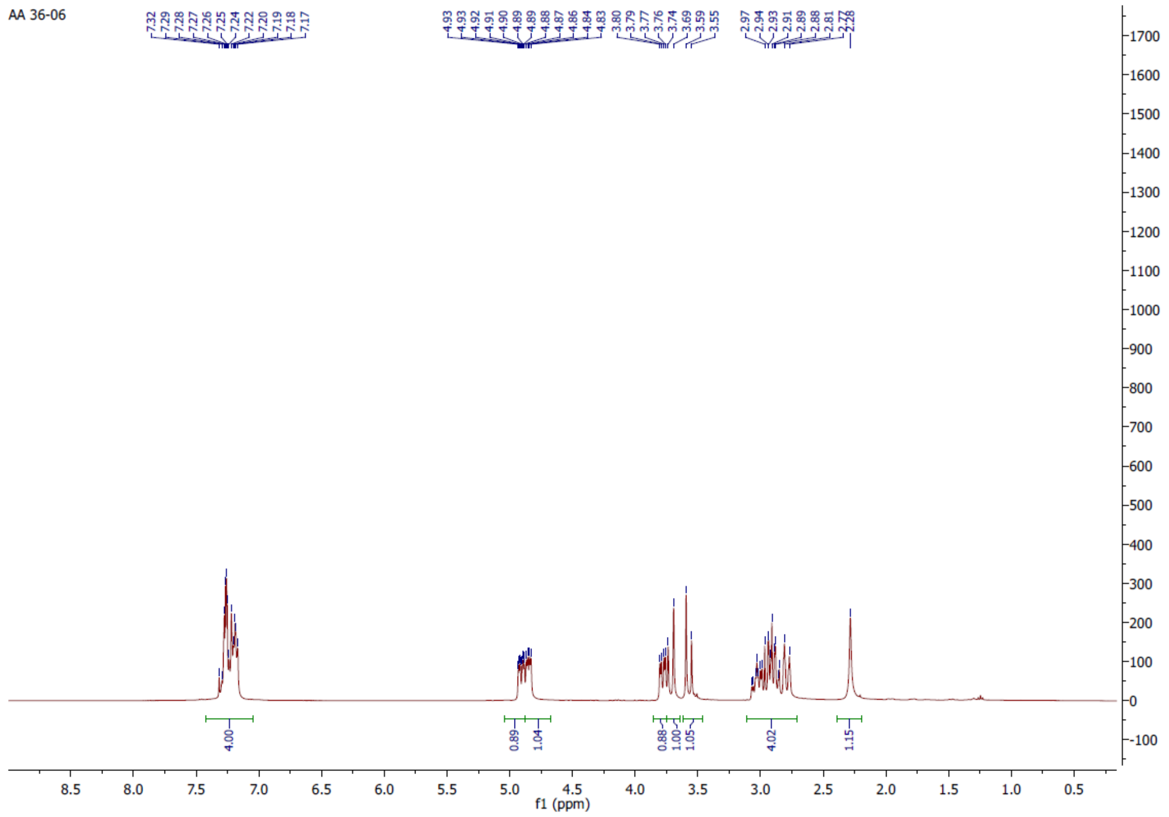

^{1}H NMR spectrum of compound **2** (400 MHz, $CDCl_3$)

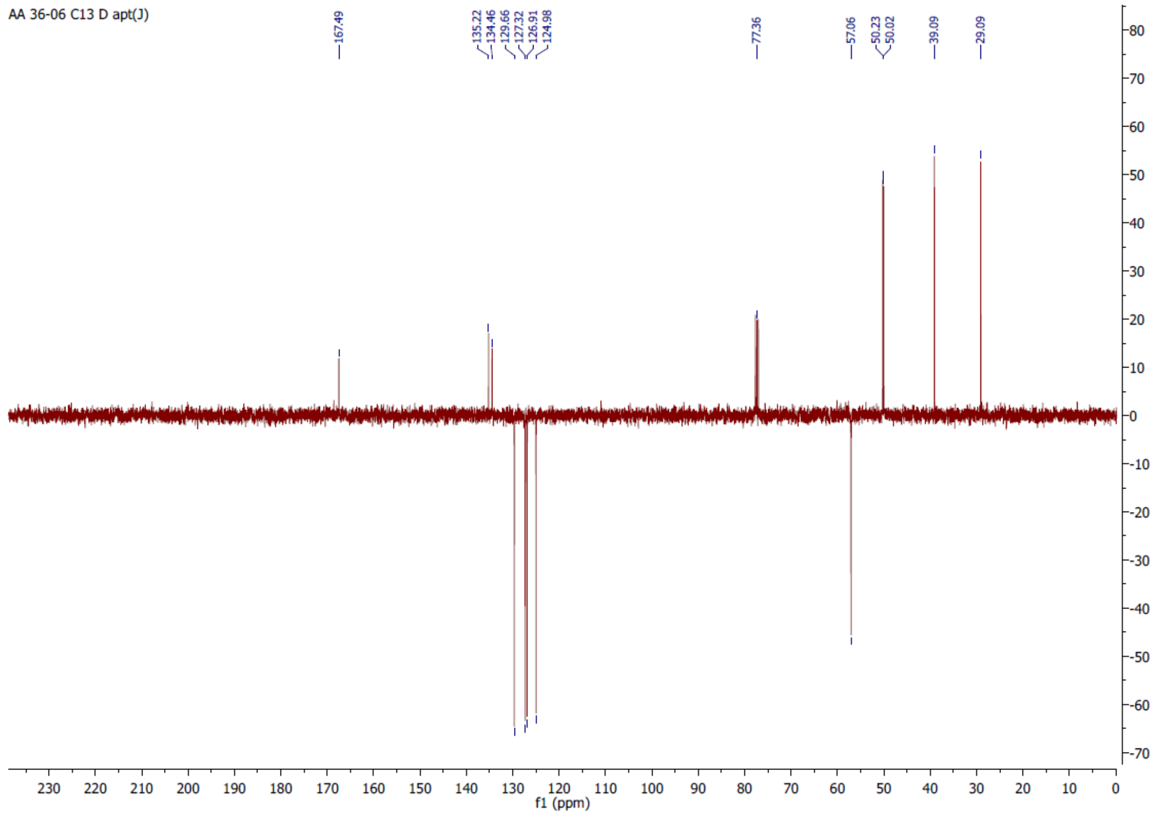

^{13}C NMR spectrum of compound **2** (100 MHz, $CDCl_3$)

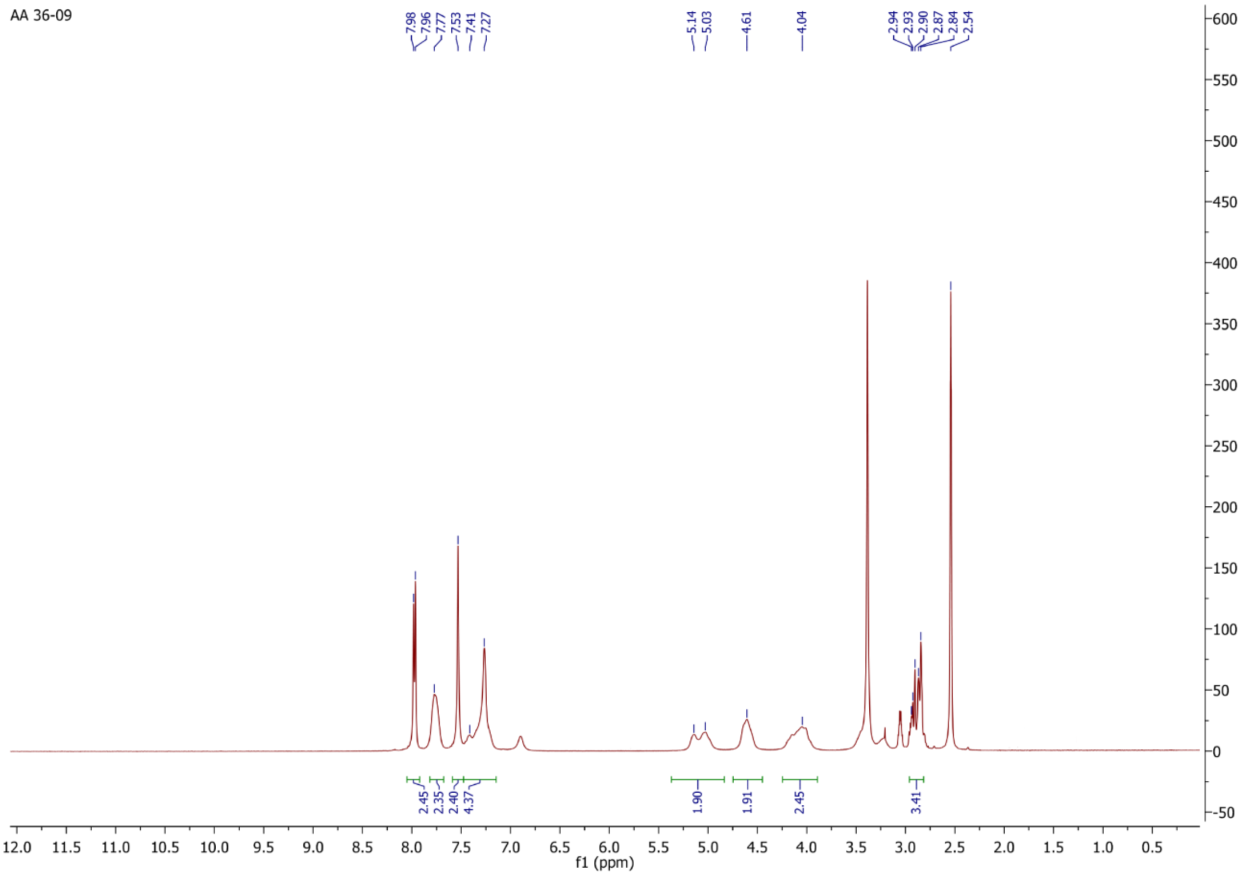

^{1}H NMR spectrum of compound **4a** (400 MHz, DMSO-d_6)

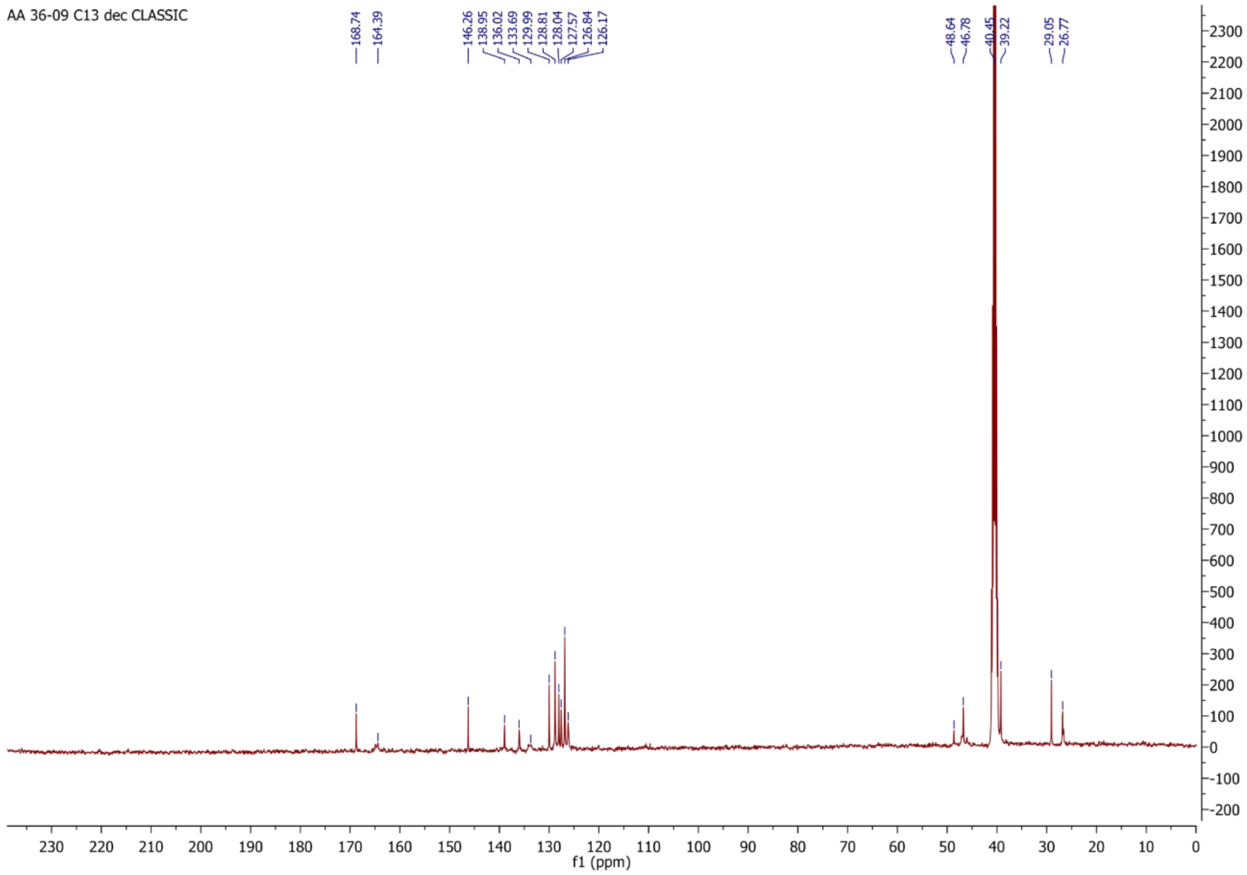

^{13}C NMR spectrum of compound **4a** (100 MHz, DMSO-d_6)

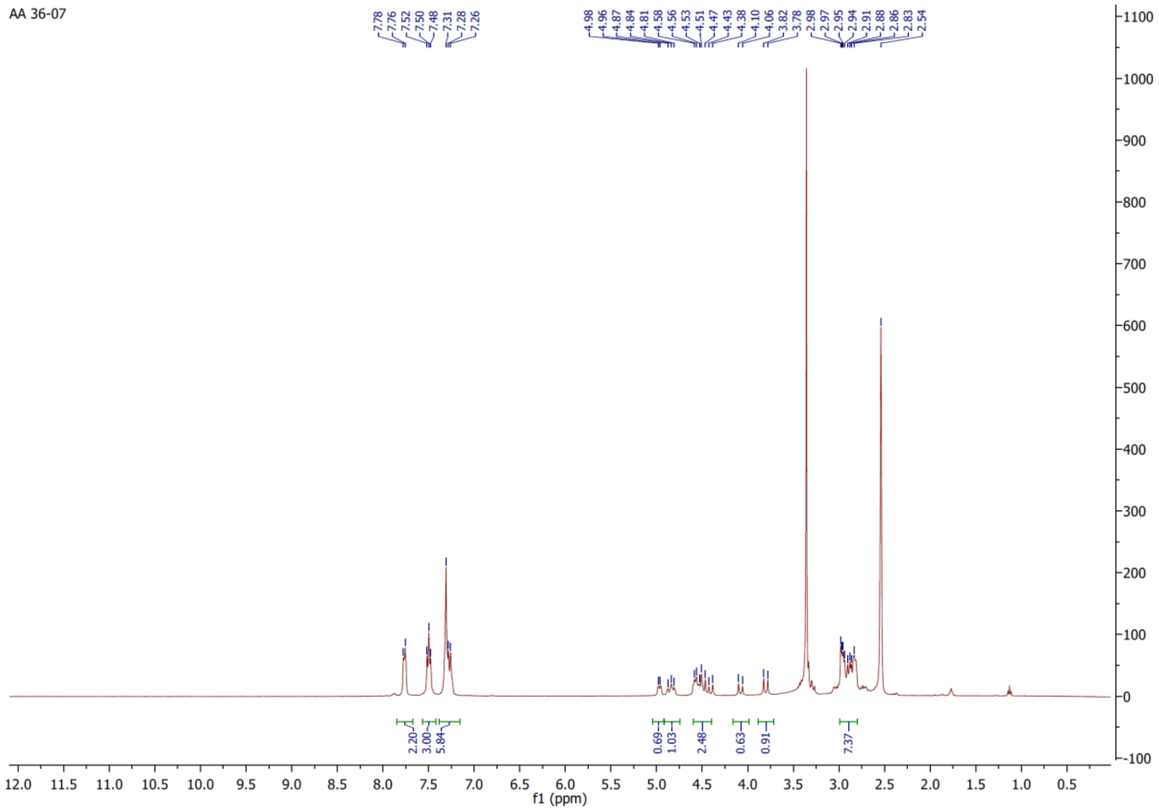

^{1}H NMR spectrum of compound **4b** (400 MHz, DMSO-d_6)

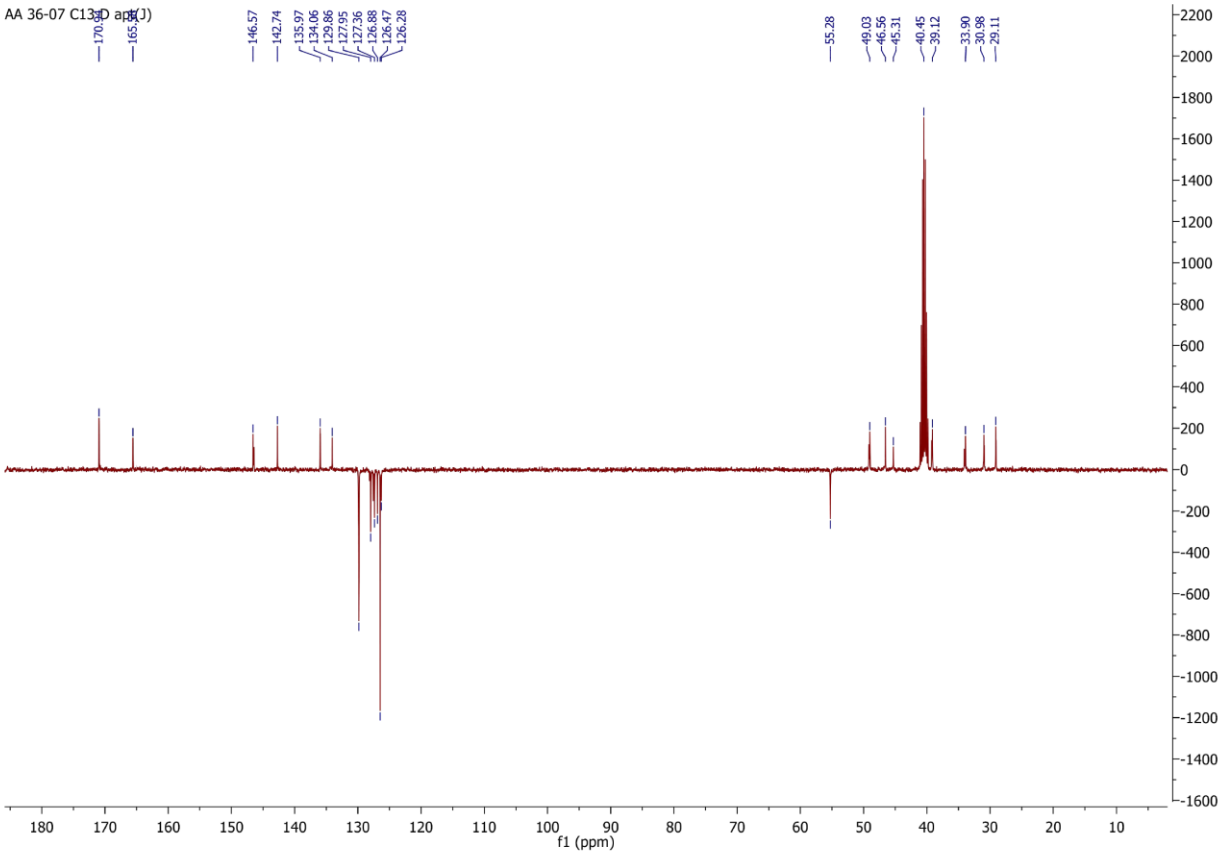

^{13}C NMR spectrum of compound **4b** (100 MHz, DMSO-d_6)

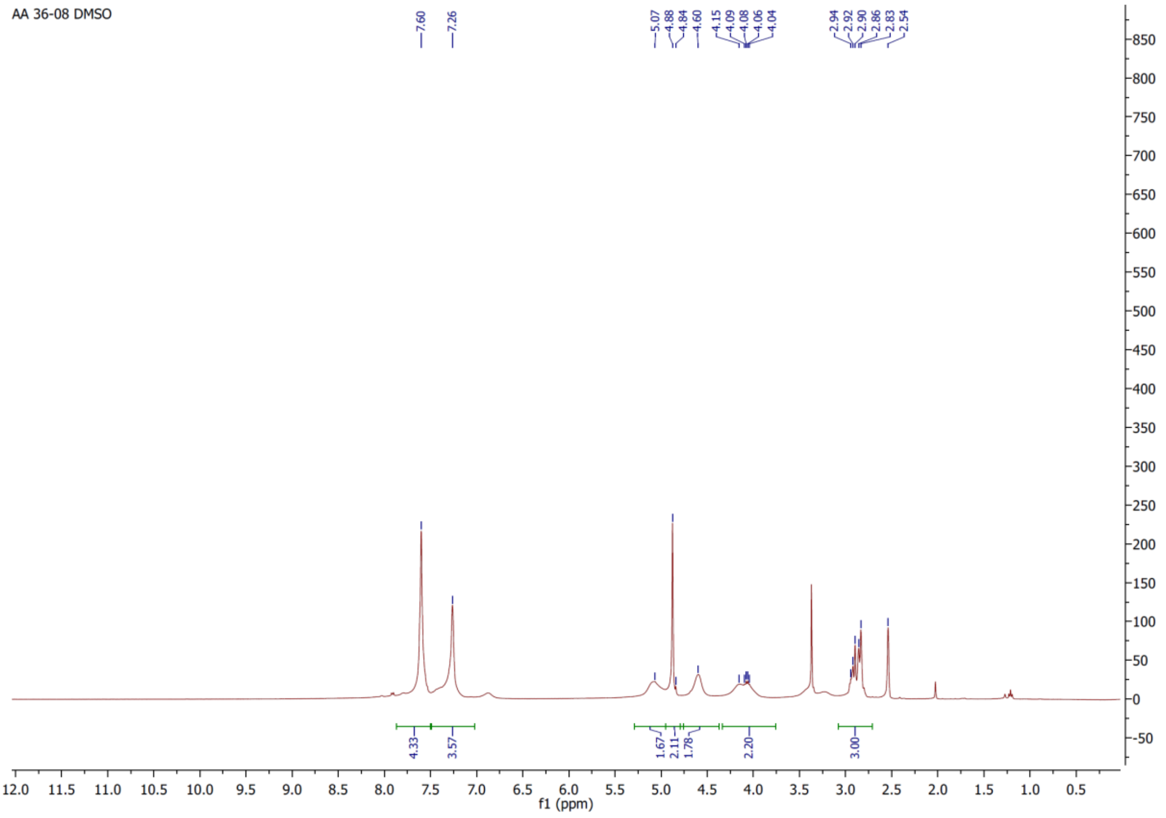

^{1}H NMR spectrum of compound **5** (400 MHz, DMSO-d_6)

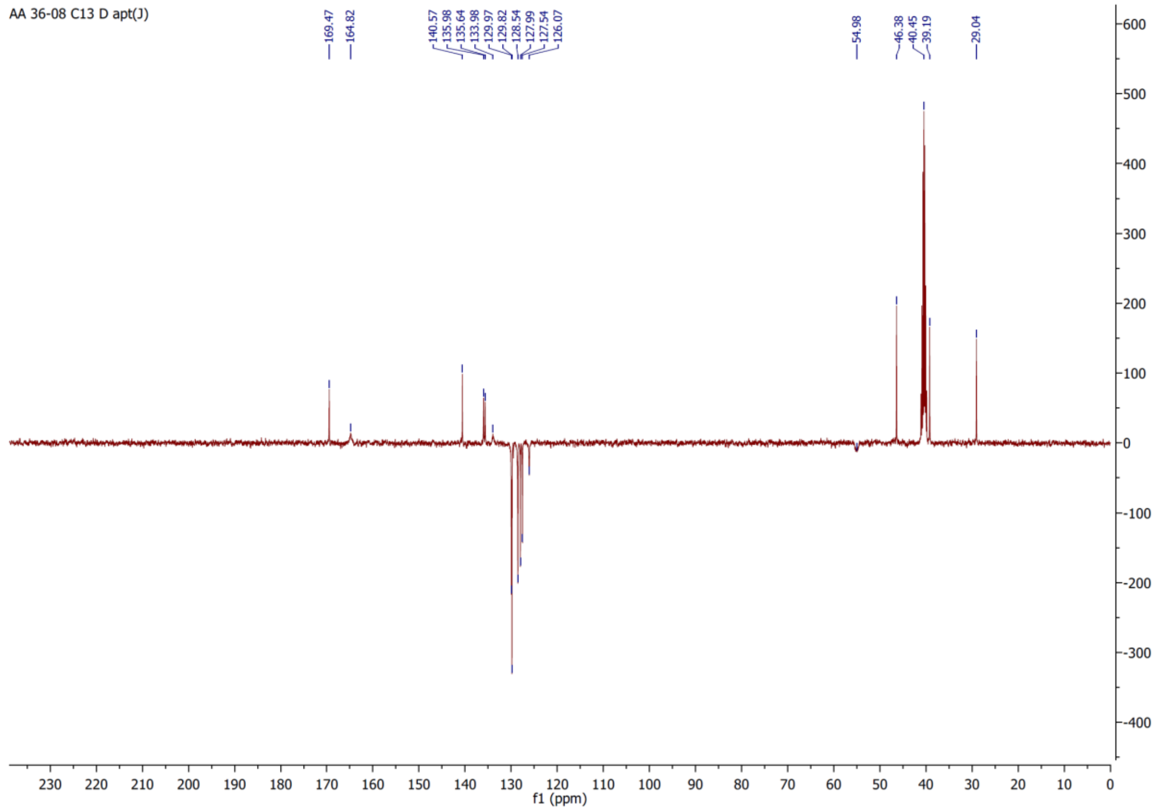

^{13}C NMR spectrum of compound **5** (100 MHz, DMSO-d_6)

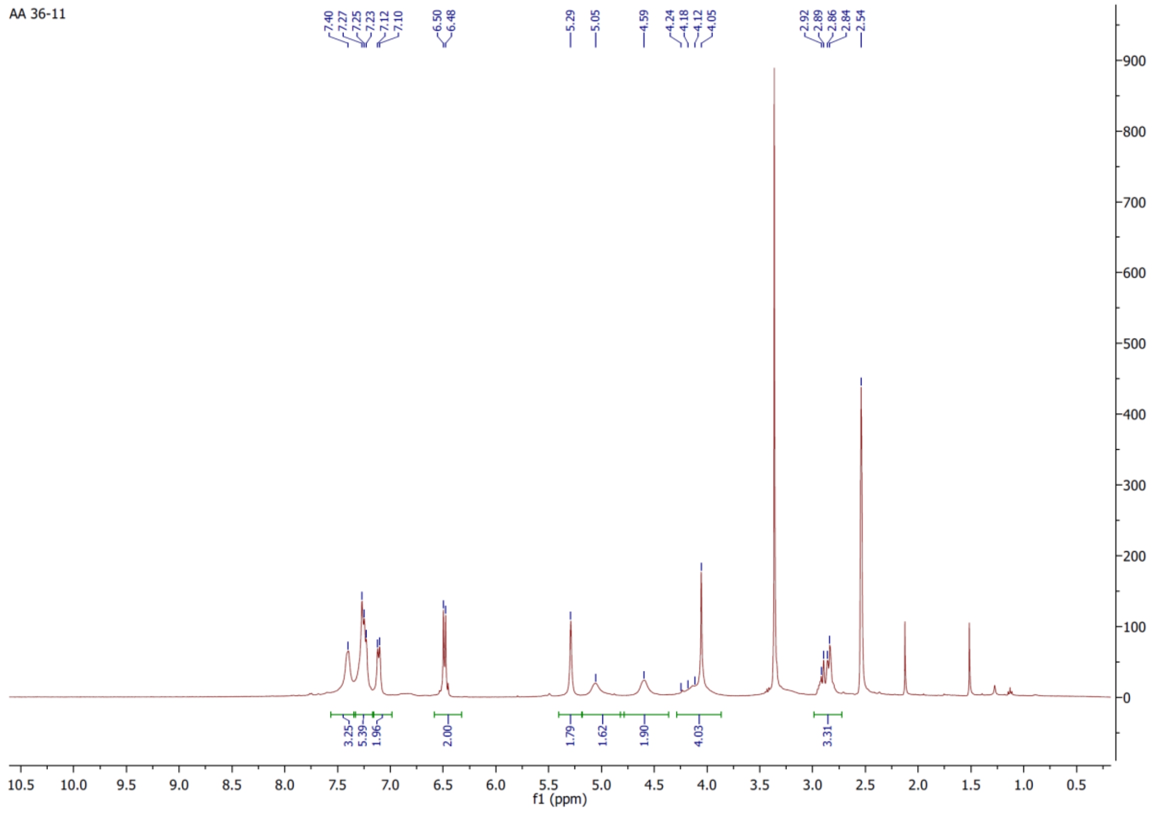

^{1}H NMR spectrum of compound **7** (400 MHz, DMSO-d_6)

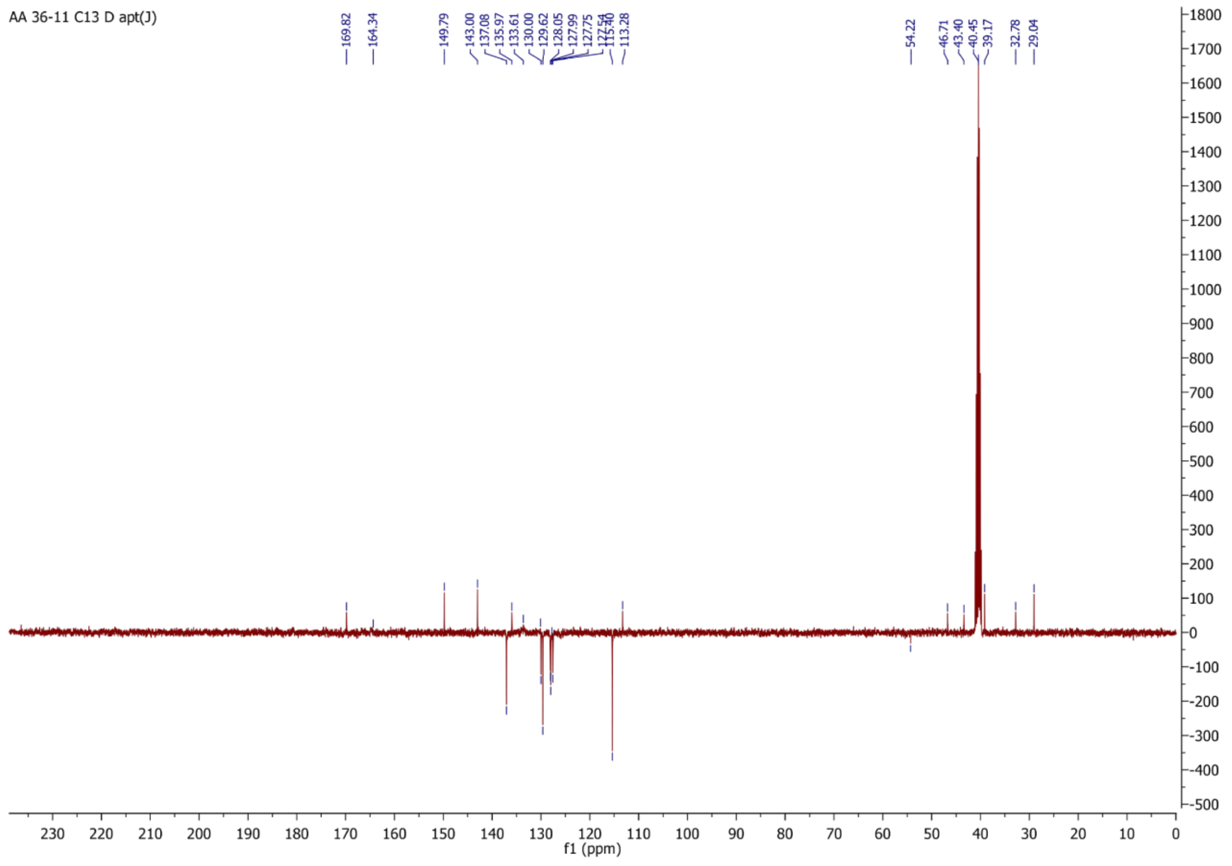

^{13}C NMR spectrum of compound **7** (100 MHz, DMSO-d_6)

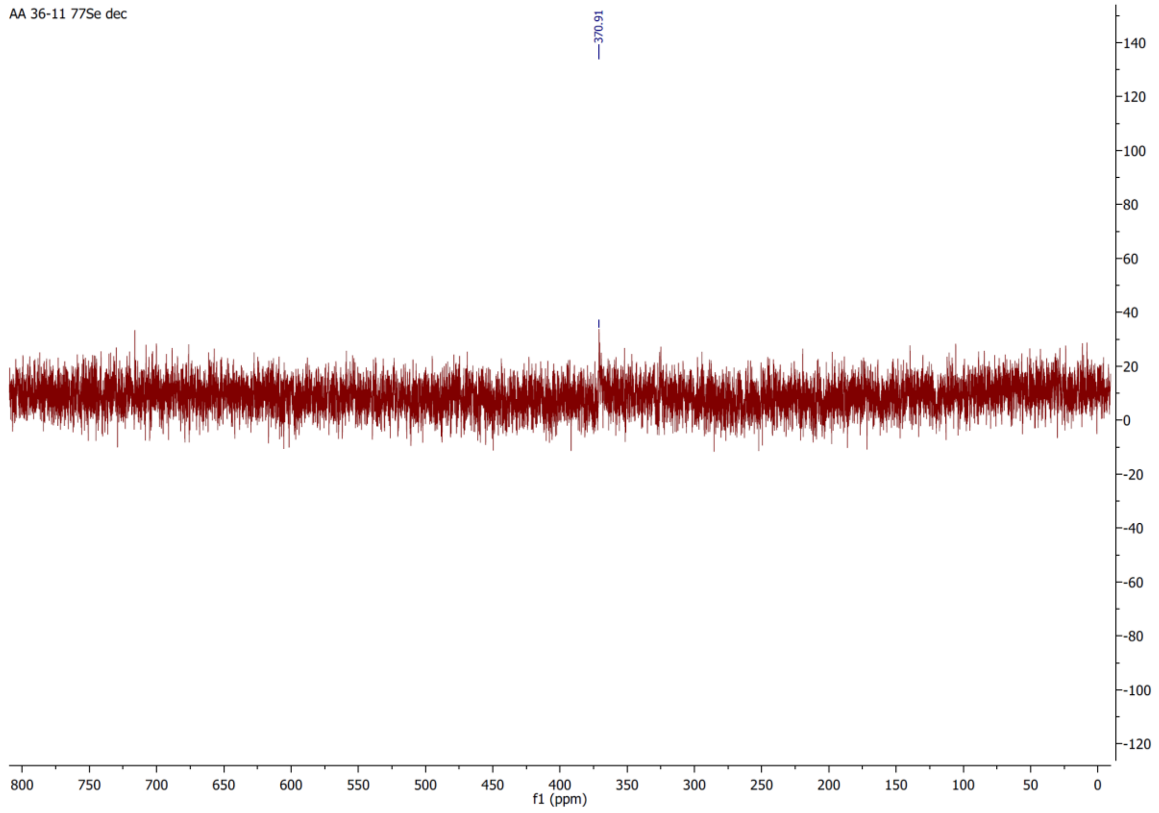

^{77}Se NMR spectrum of compound **7** (76 MHz, DMSO-d_6)

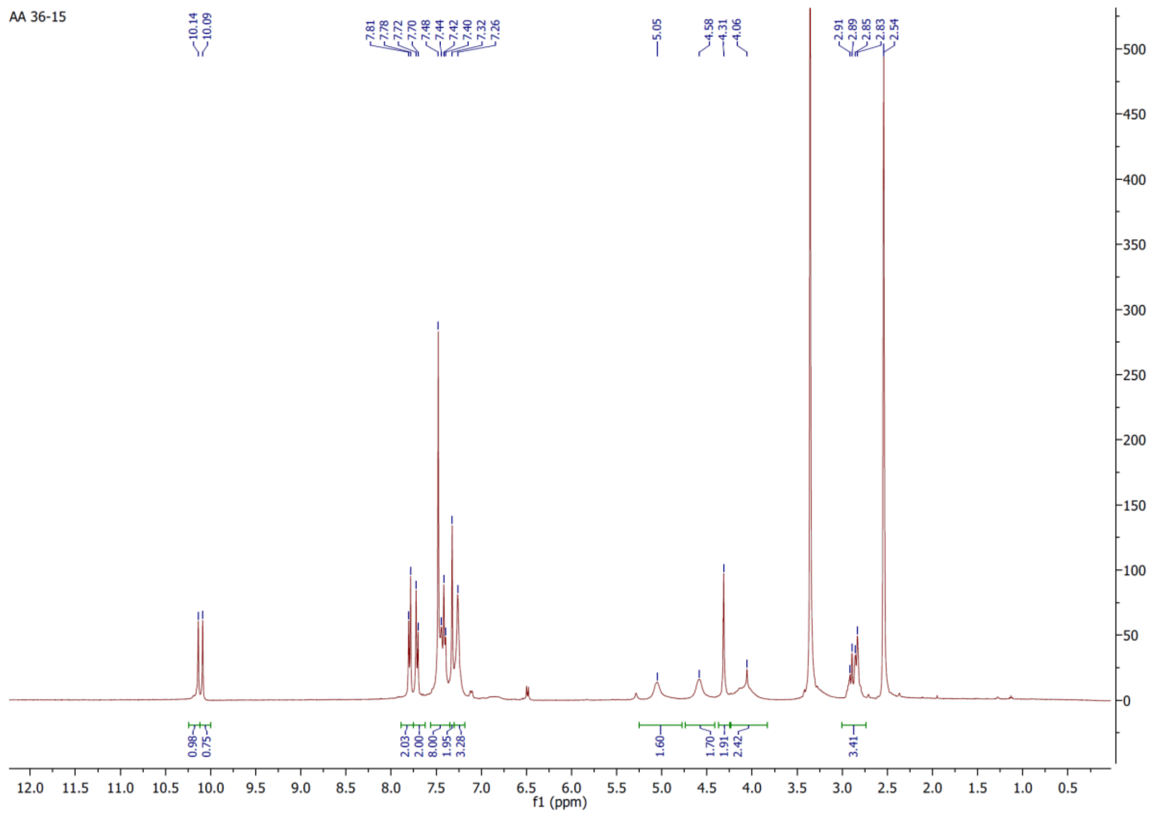

^{1}H NMR spectrum of compound **9** (400 MHz, DMSO-d_6)

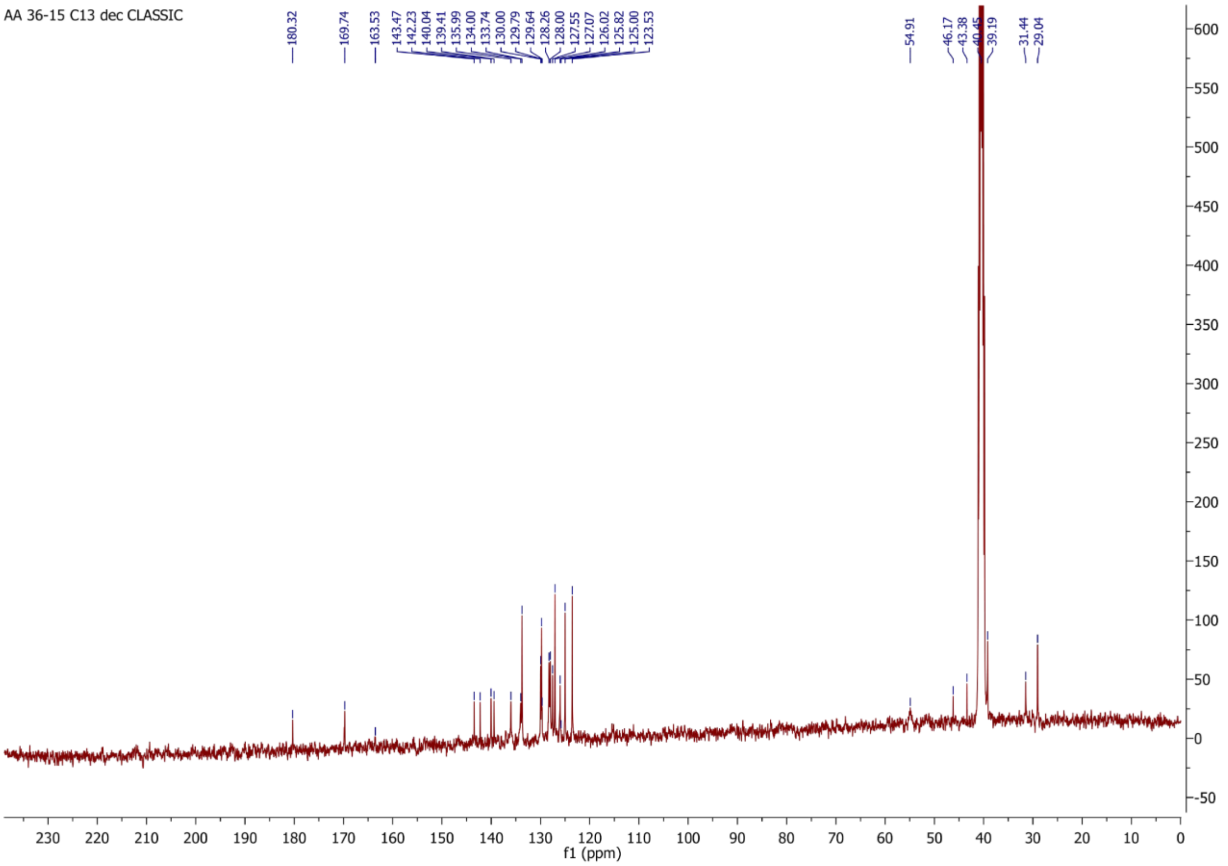

^{13}C NMR spectrum of compound **9** (100 MHz, DMSO-d_6)

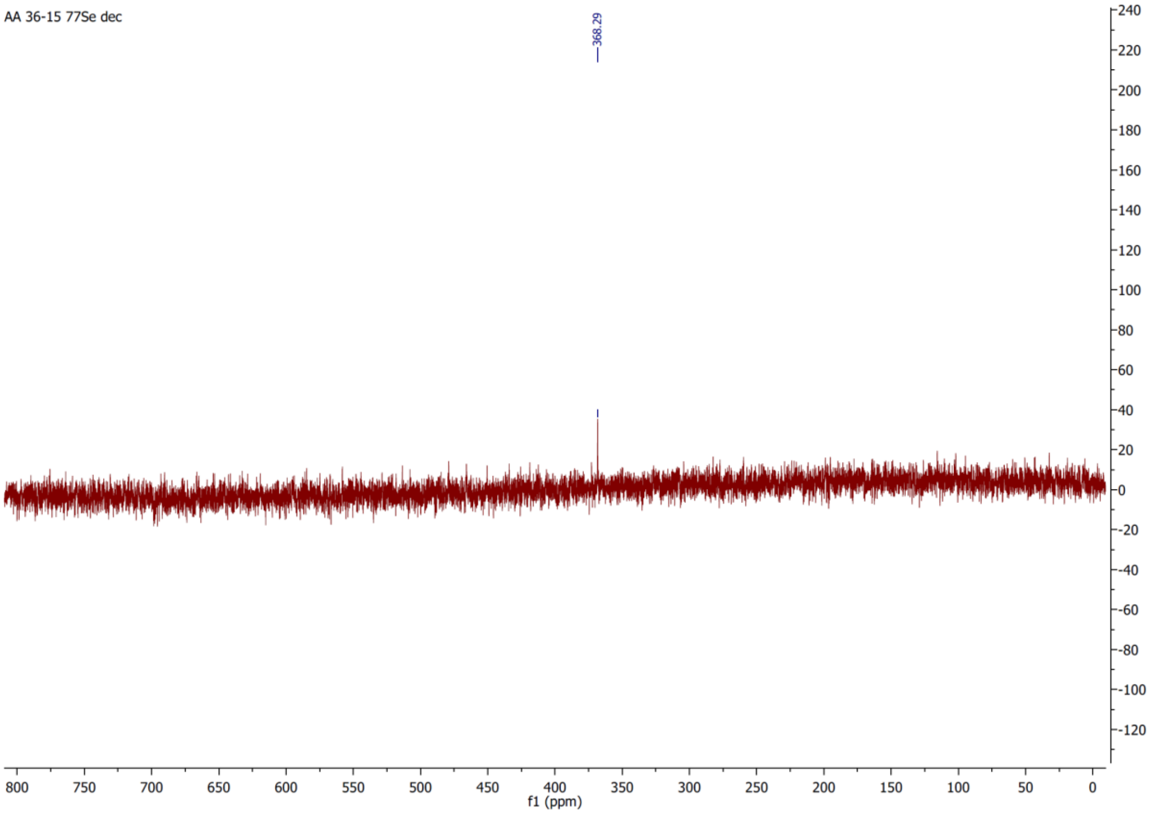

^{77}Se NMR spectrum of compound **9** (76 MHz, DMSO-d_6)

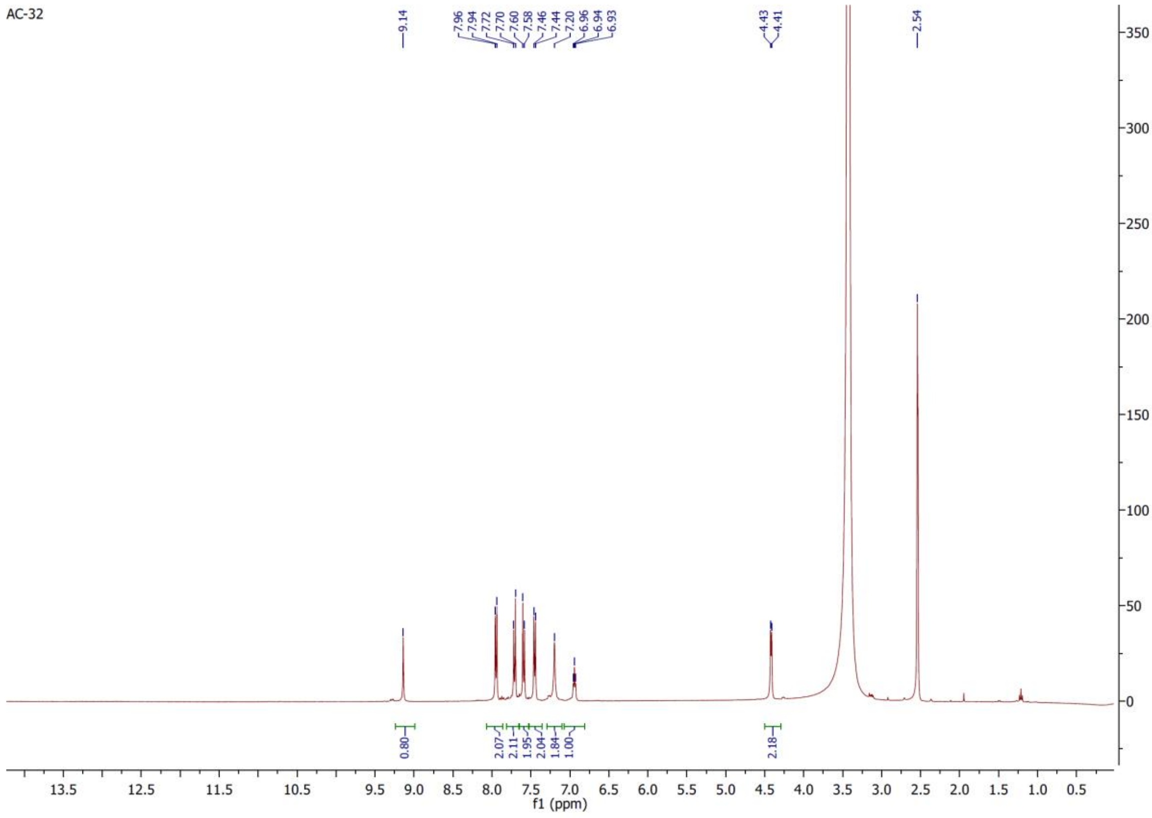

^{1}H NMR spectrum of compound **12a** (400 MHz, DMSO-d_6)

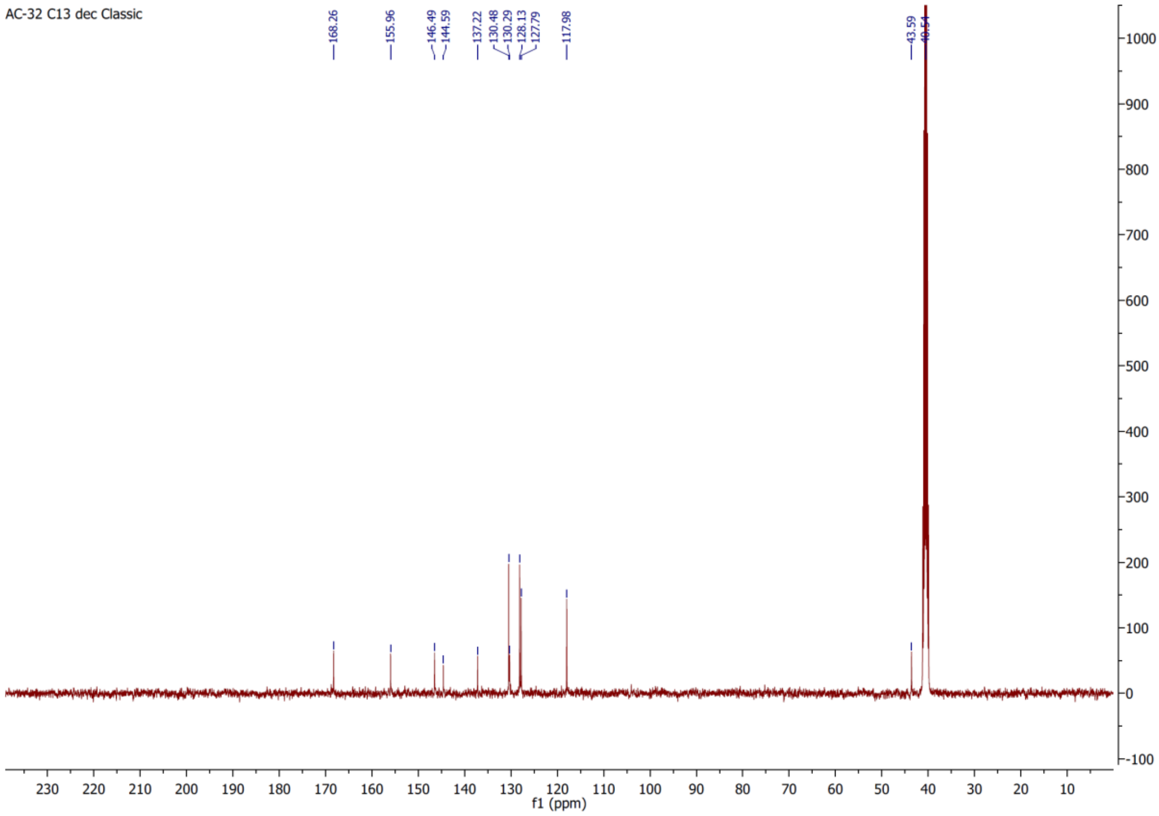

^{13}C NMR spectrum of compound **12a** (100 MHz, DMSO-d_6)

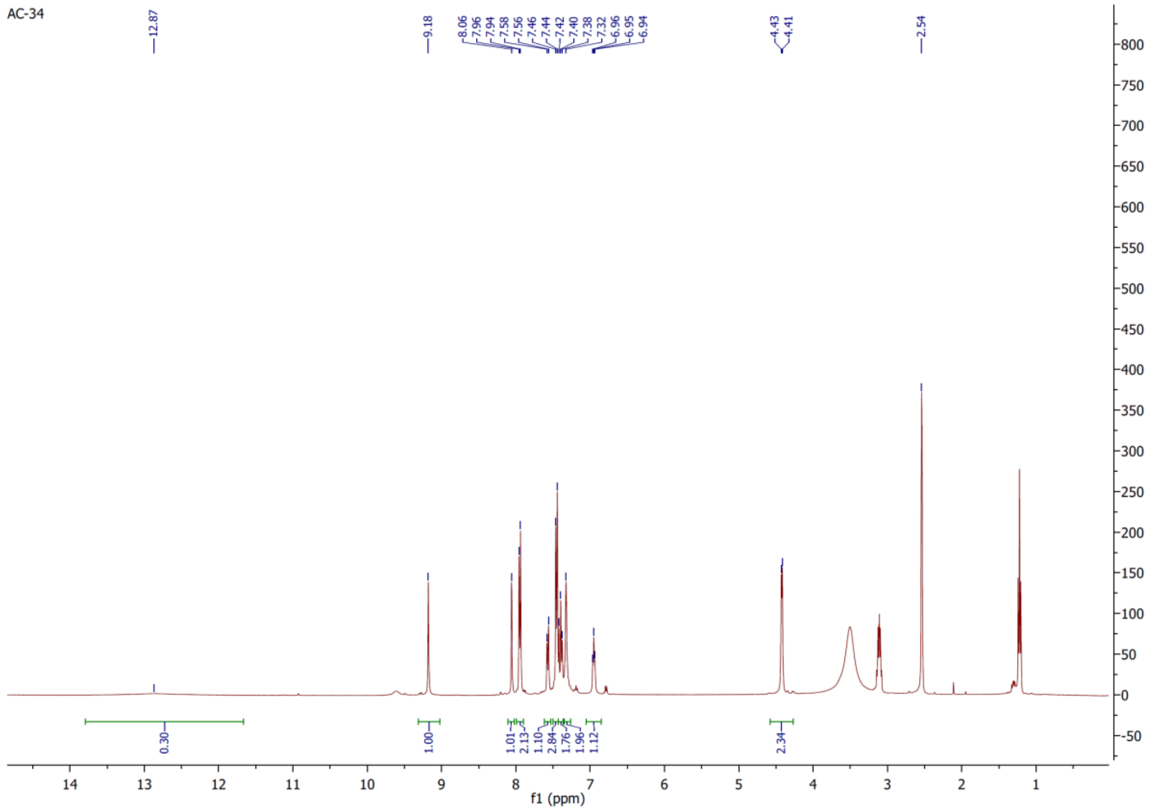

^{1}H NMR spectrum of compound **12b** (400 MHz, DMSO-d_6)

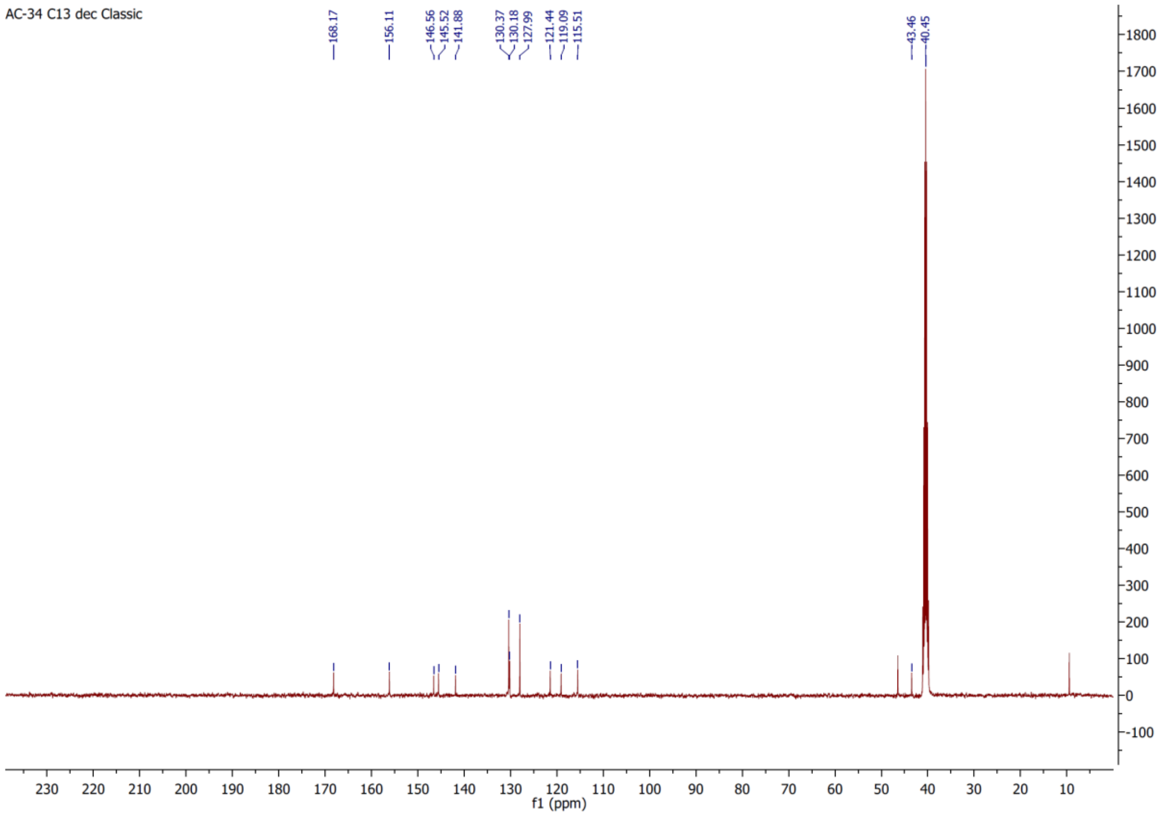

^{13}C NMR spectrum of compound **12b** (100 MHz, DMSO-d_6)

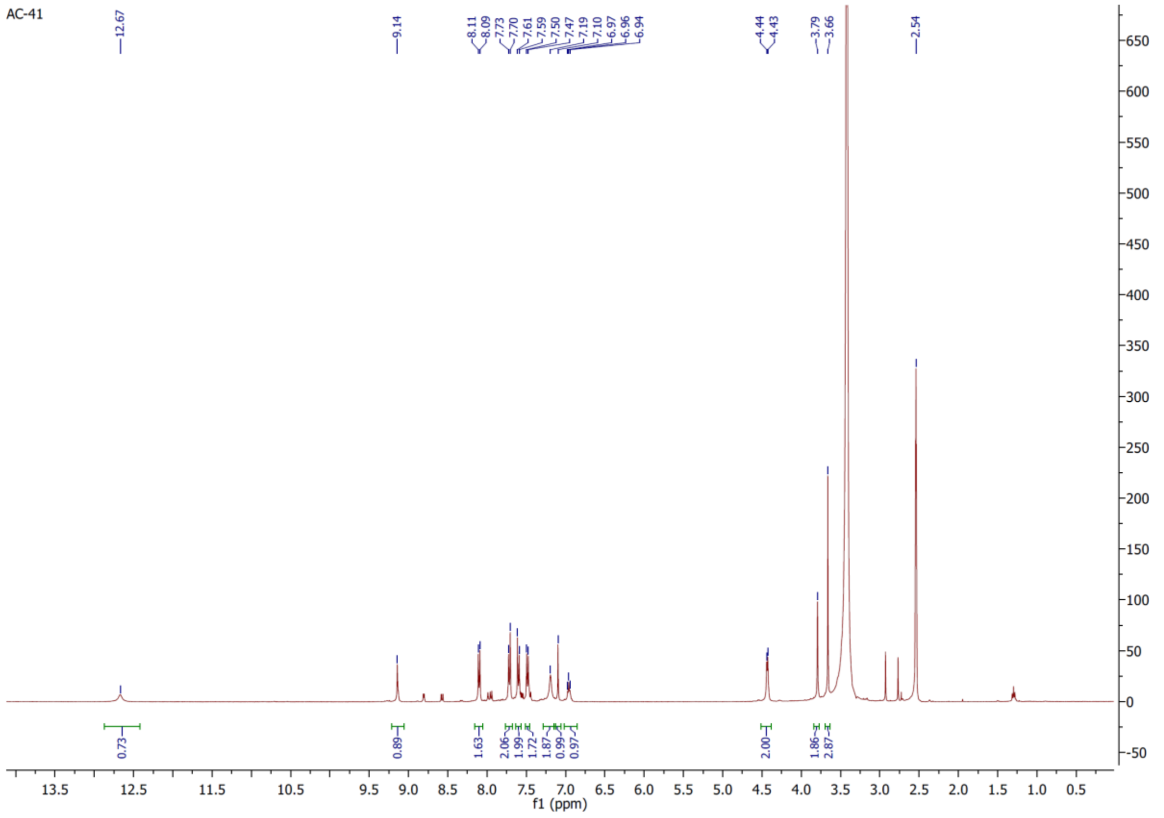

^{1}H NMR spectrum of compound **15a** (400 MHz, DMSO-d_6)

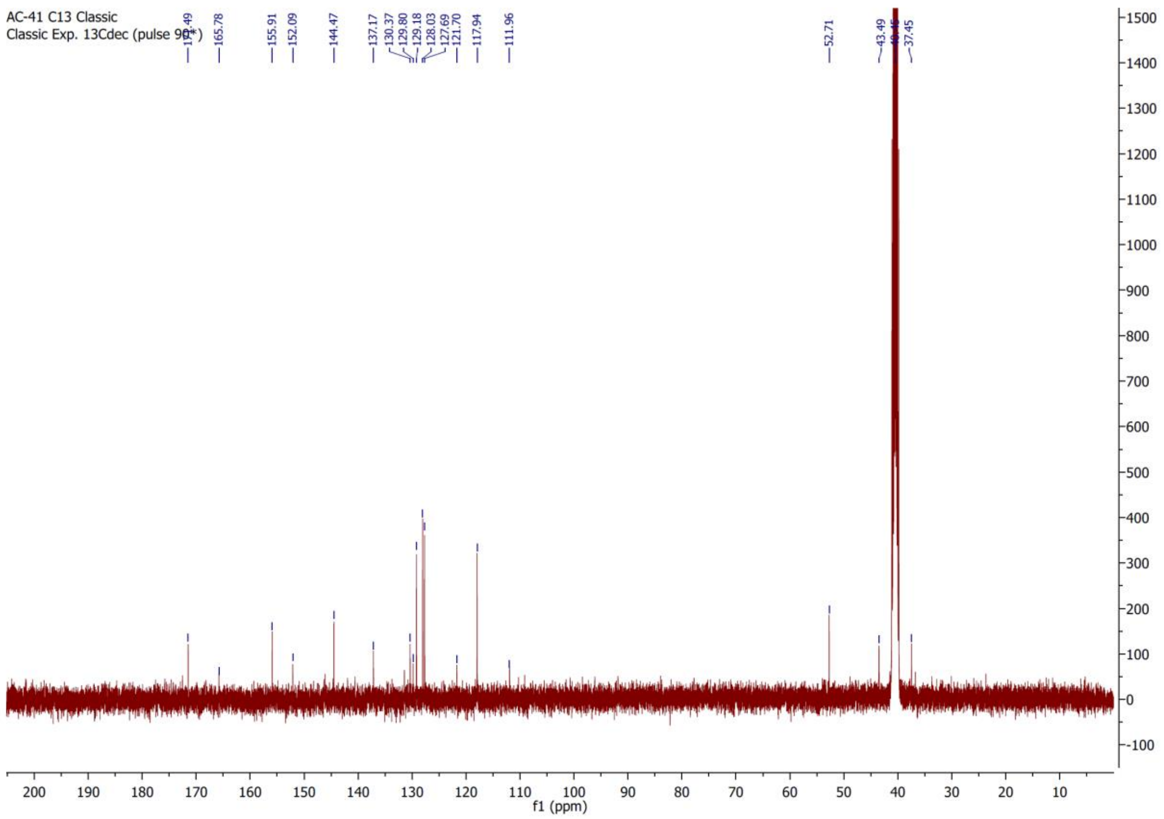

^{13}C NMR spectrum of compound **15a** (100 MHz, DMSO-d_6)

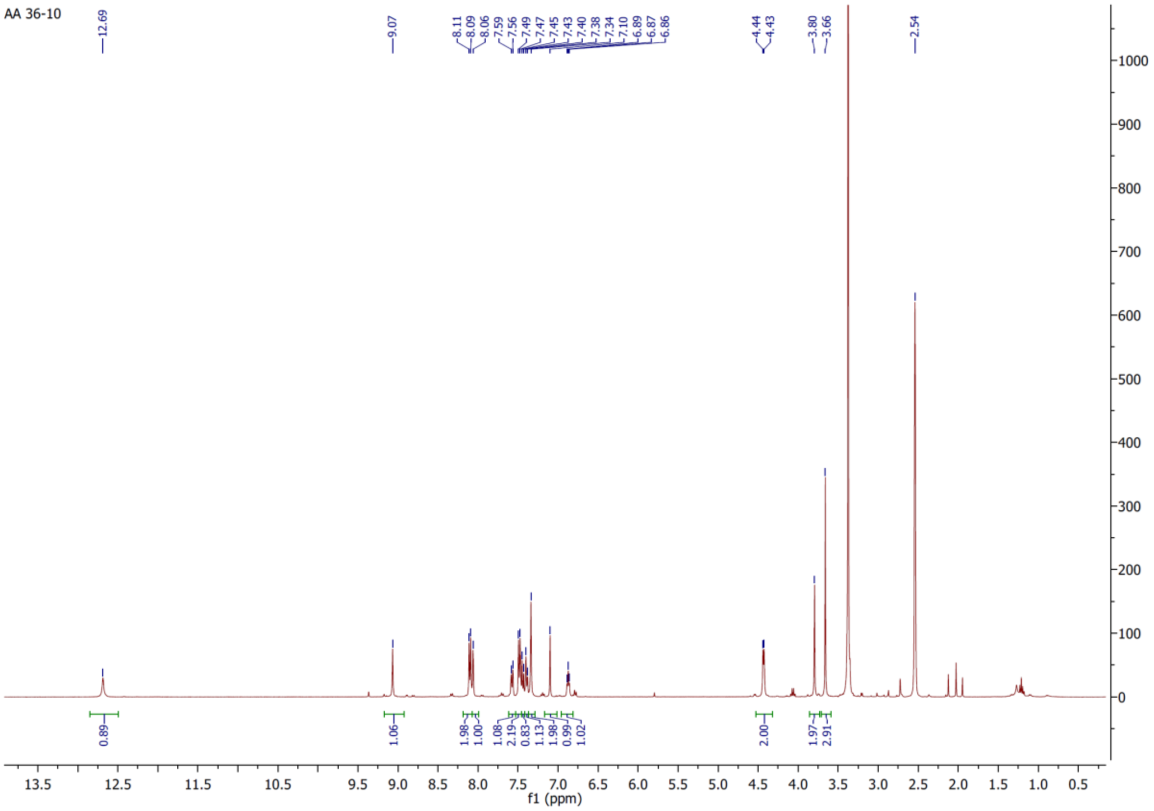

^{1}H NMR spectrum of compound **15b** (400 MHz, DMSO-d_6)

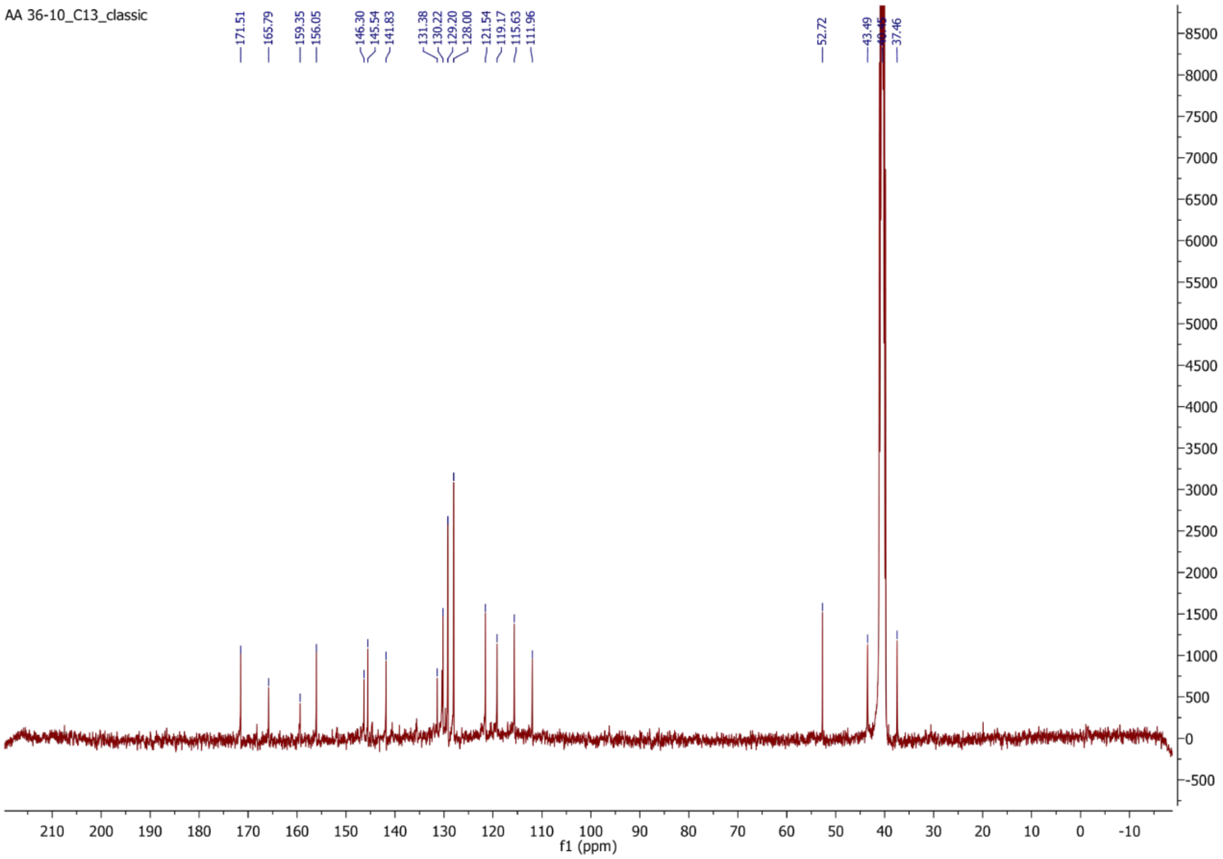

^{13}C NMR spectrum of compound **15b** (100 MHz, DMSO-d_6)

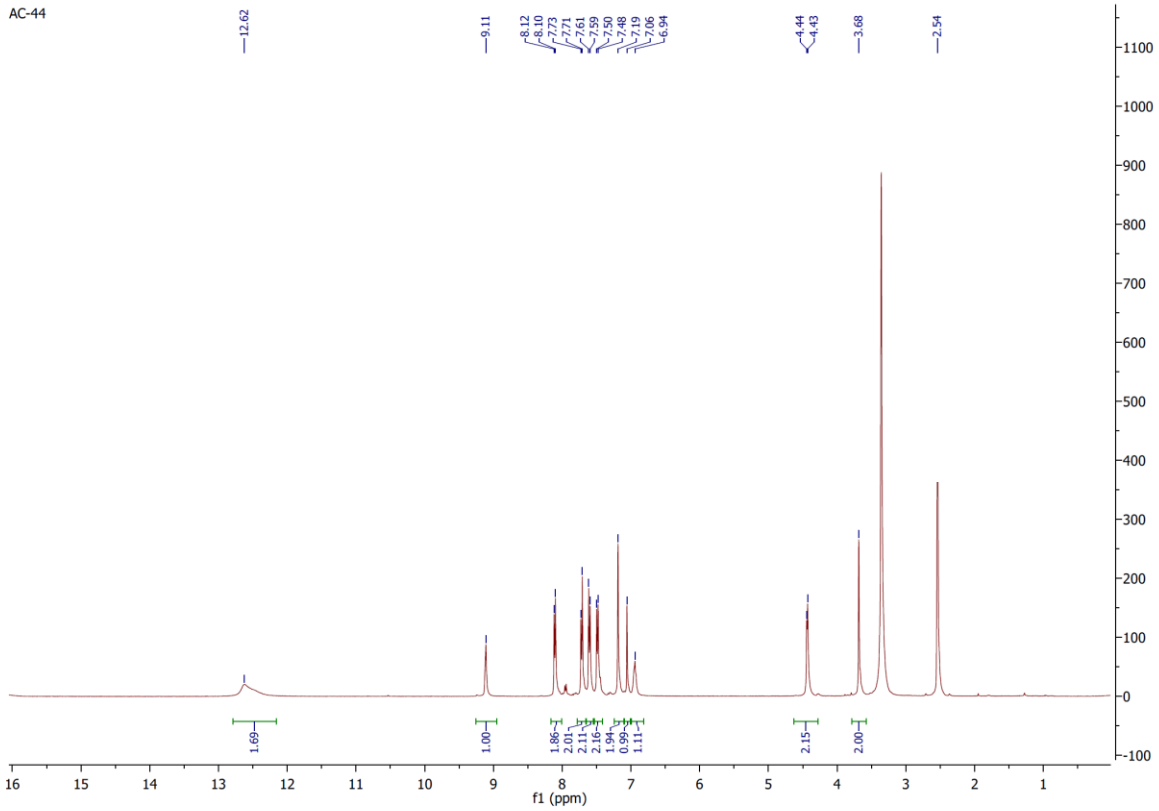

^{1}H NMR spectrum of compound **16a** (400 MHz, DMSO-d_6)

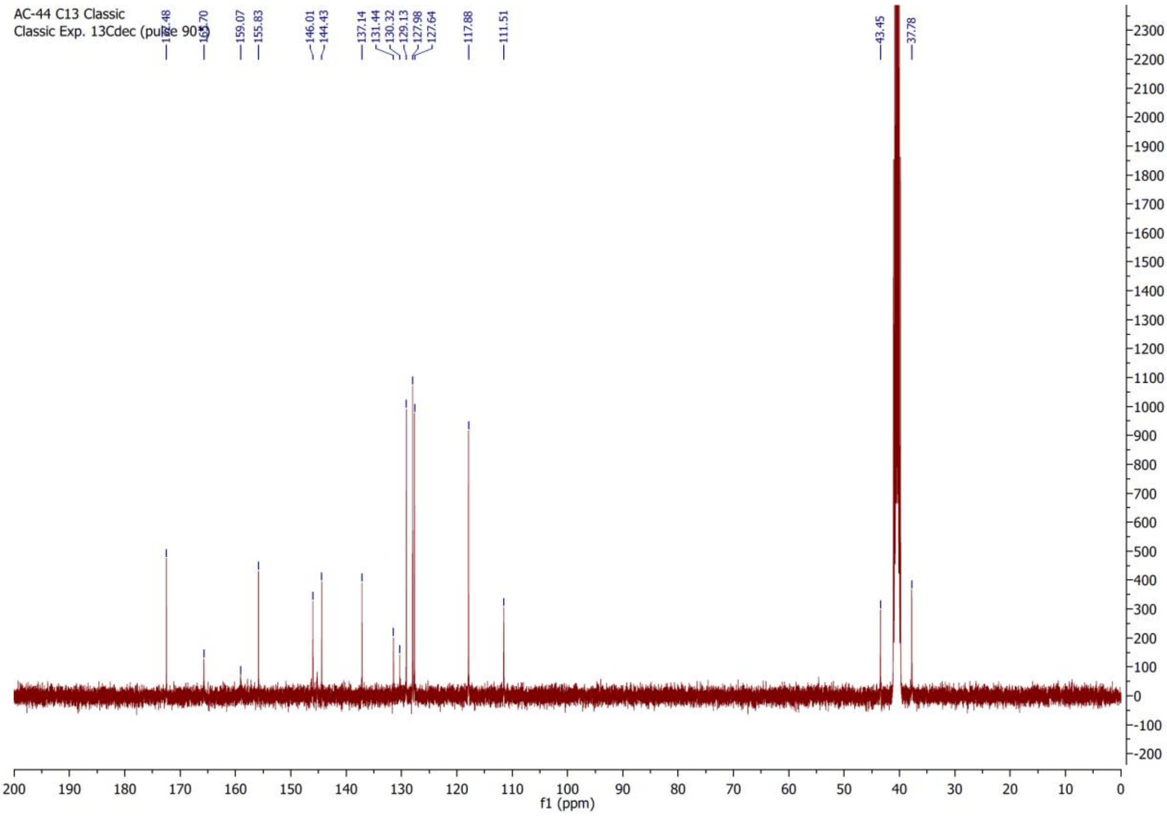

^{13}C NMR spectrum of compound **16a** (100 MHz, DMSO-d_6)

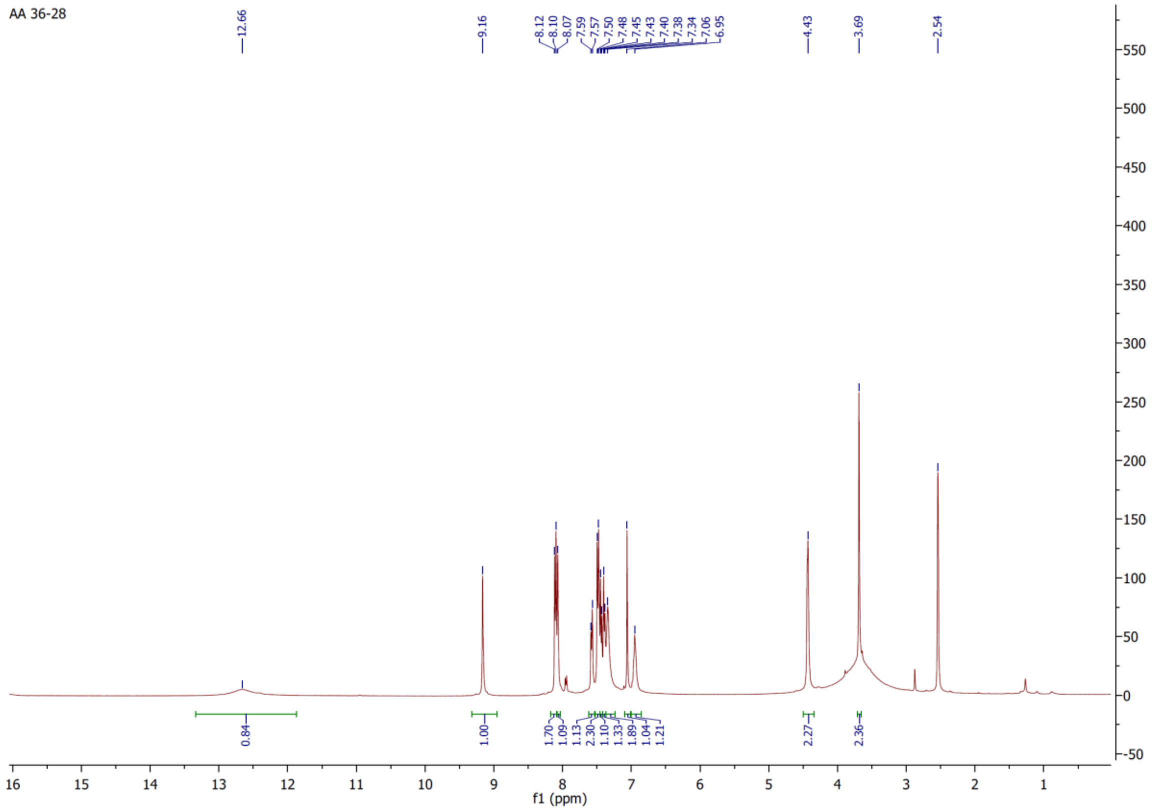

^{1}H NMR spectrum of compound **16b** (400 MHz, DMSO-d_6)

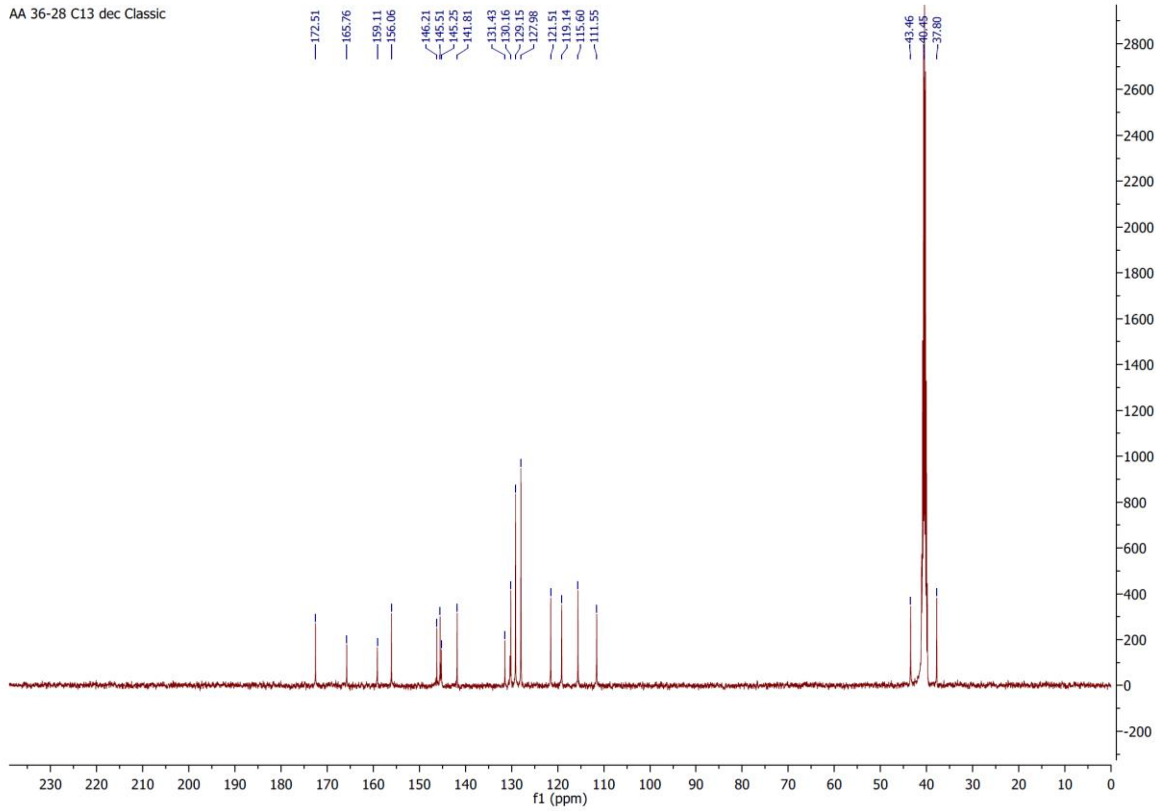

^{13}C NMR spectrum of compound **16b** (100 MHz, DMSO-d_6)

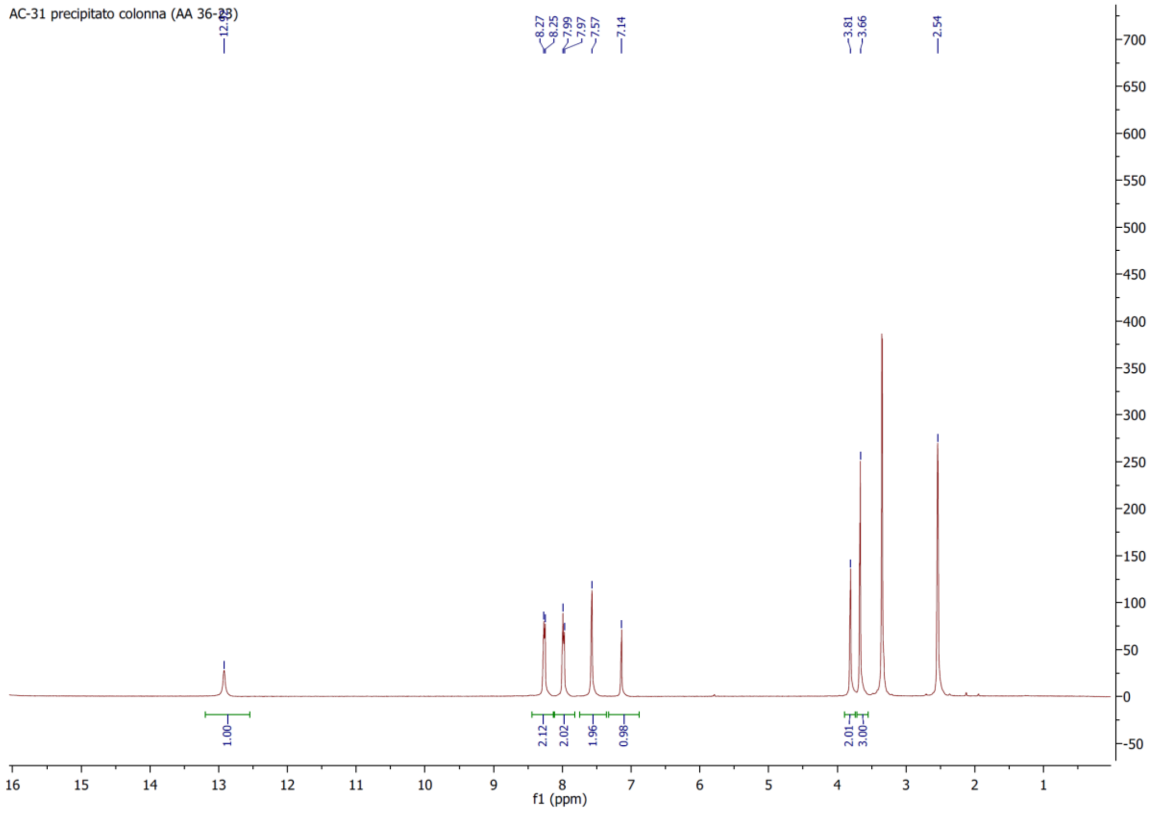

^{1}H NMR spectrum of compound **17** (400 MHz, DMSO-d_6)

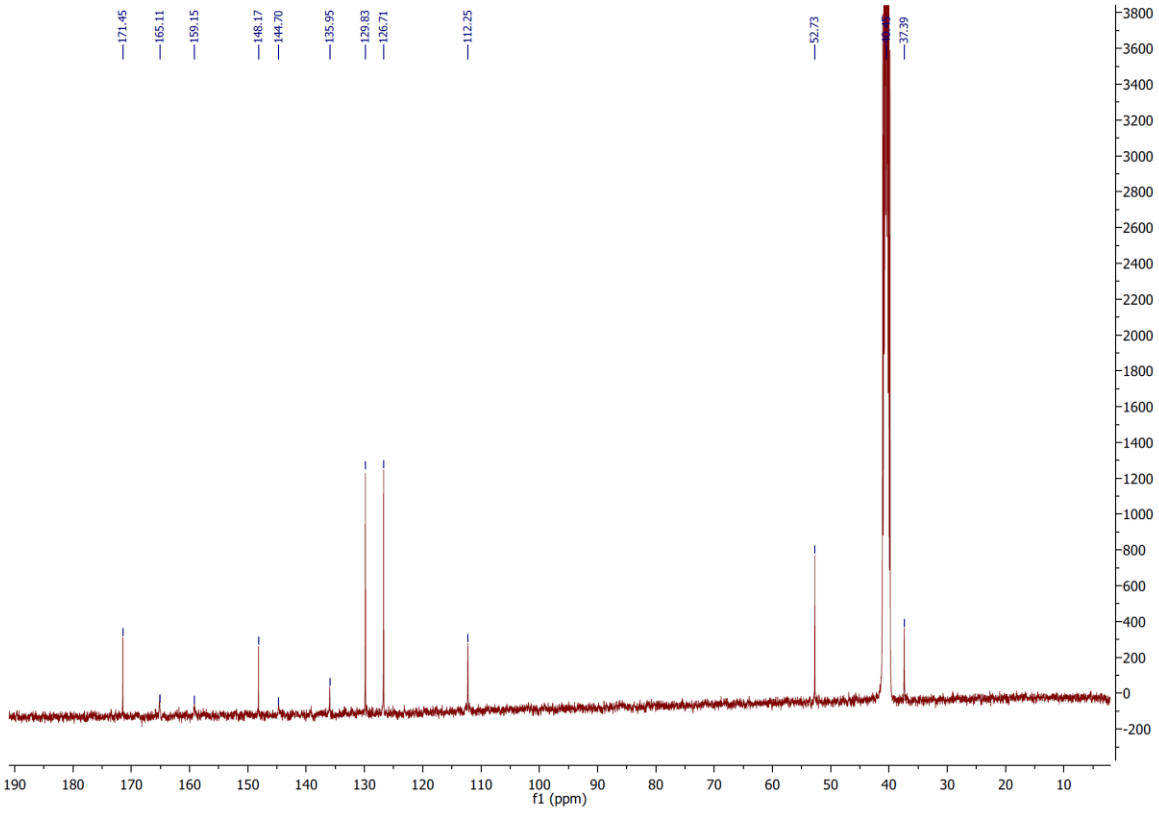

^{13}C NMR spectrum of compound **17** (100 MHz, DMSO-d_6)

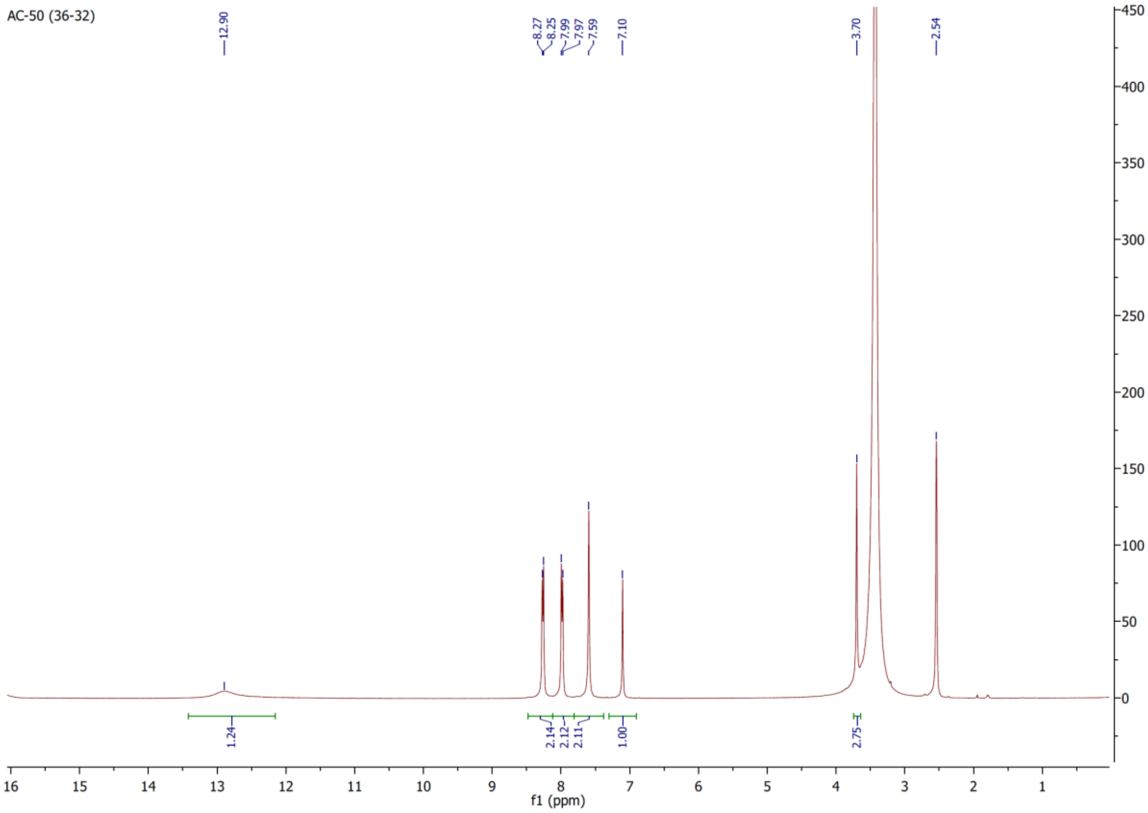

^{1}H NMR spectrum of compound **18** (400 MHz, DMSO-d_6)

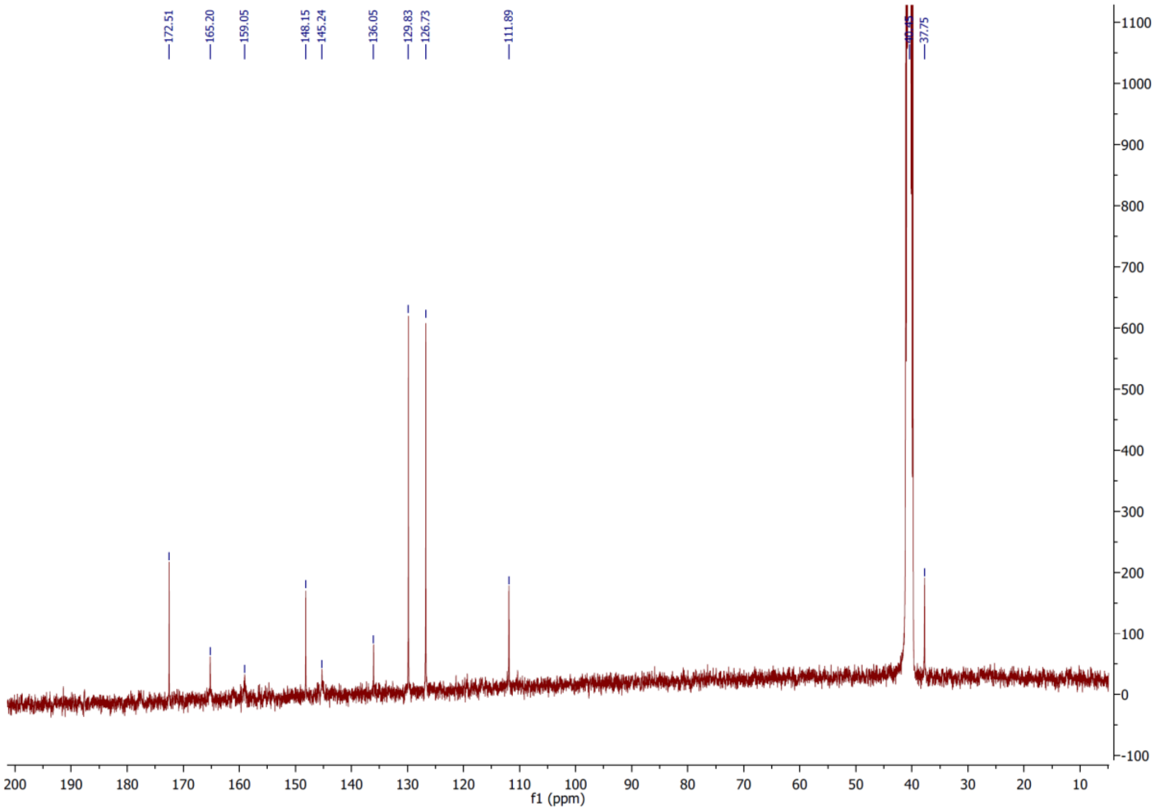

^{13}C NMR spectrum of compound **18** (100 MHz, DMSO-d_6)

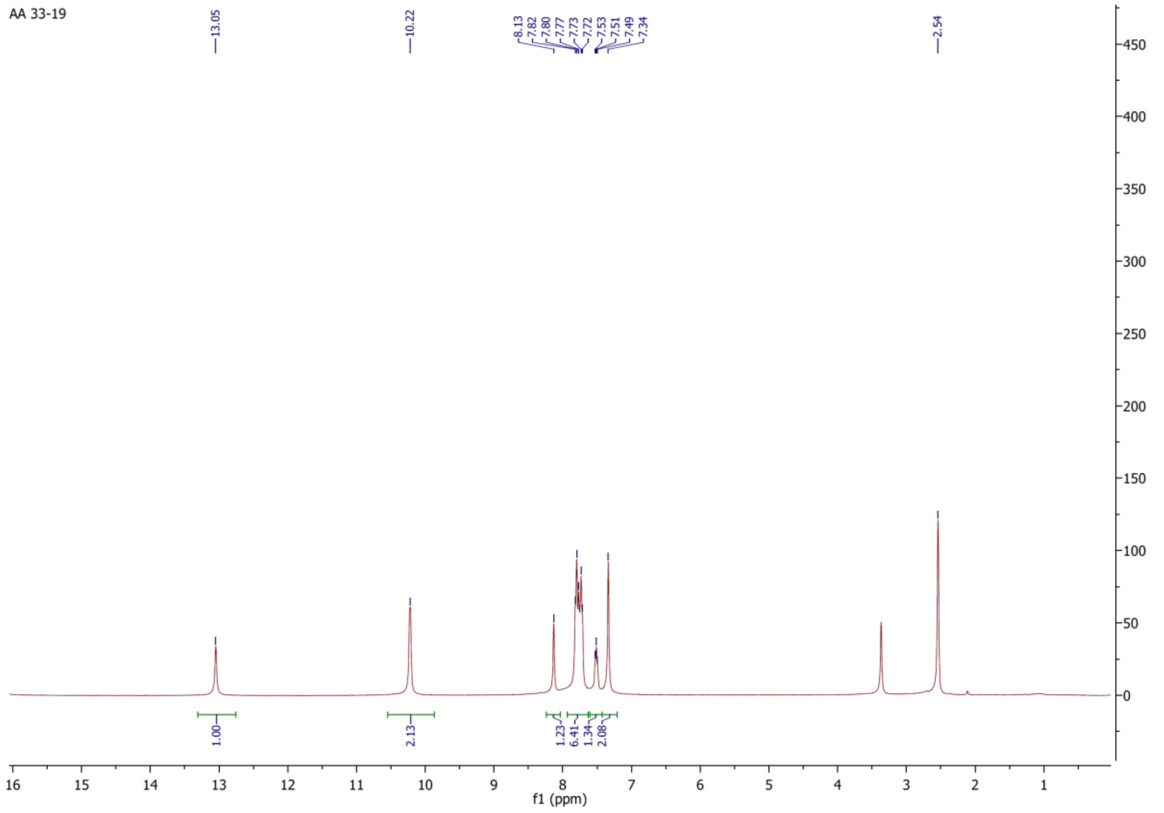

^{1}H NMR spectrum of compound **20a** (400 MHz, DMSO-d_6)

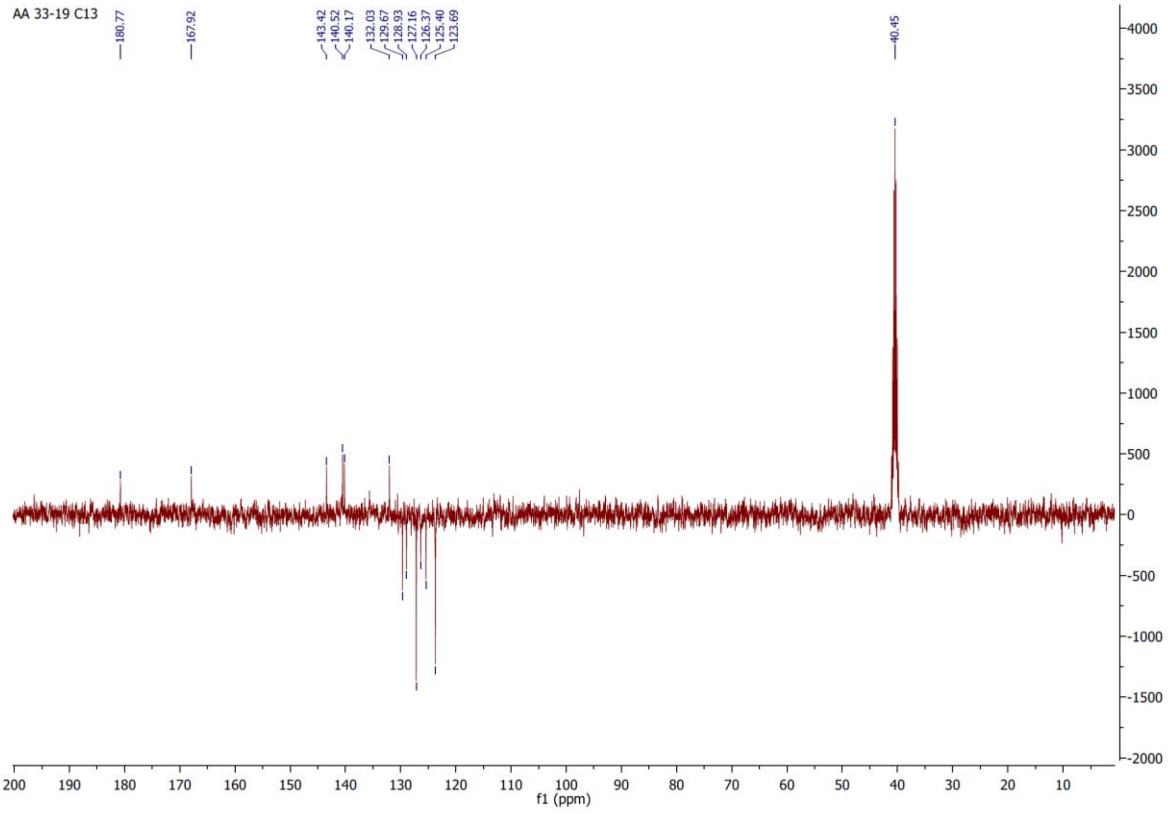

^{13}C NMR spectrum of compound **20a** (100 MHz, DMSO-d_6)

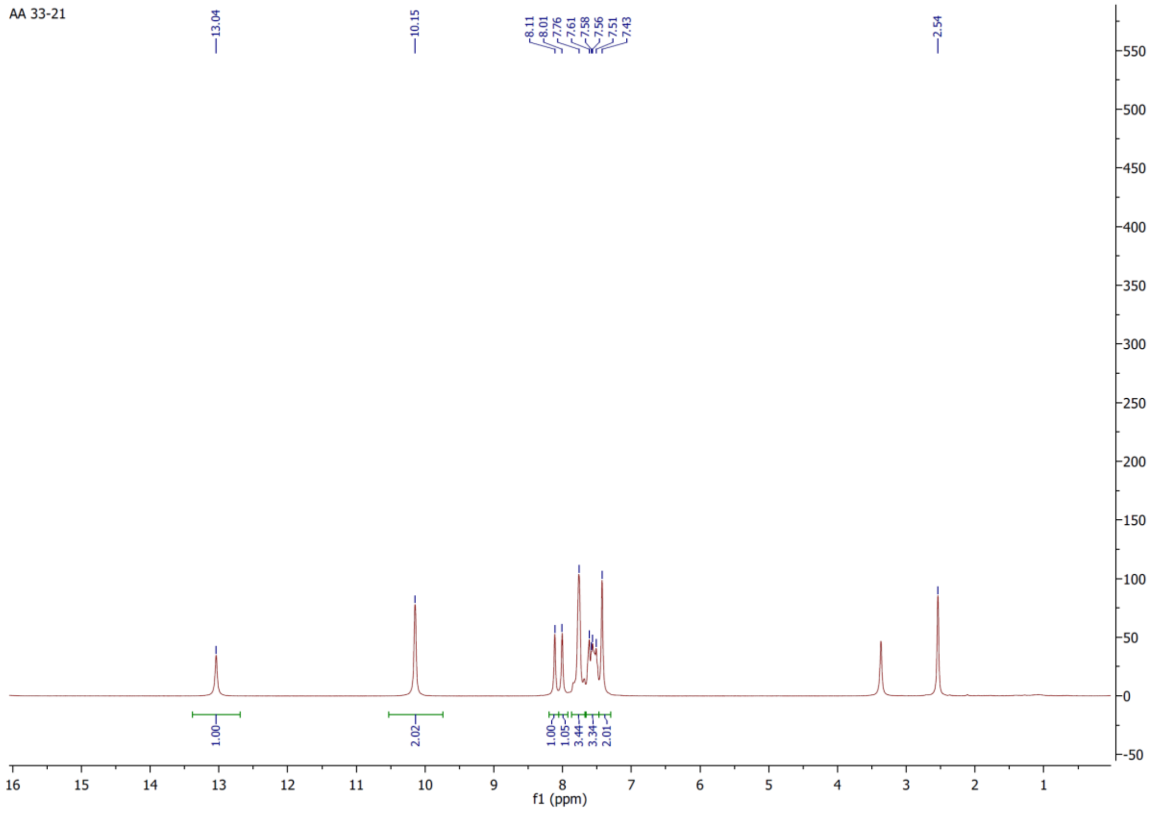

^{1}H NMR spectrum of compound **20b** (400 MHz, DMSO-d_6)

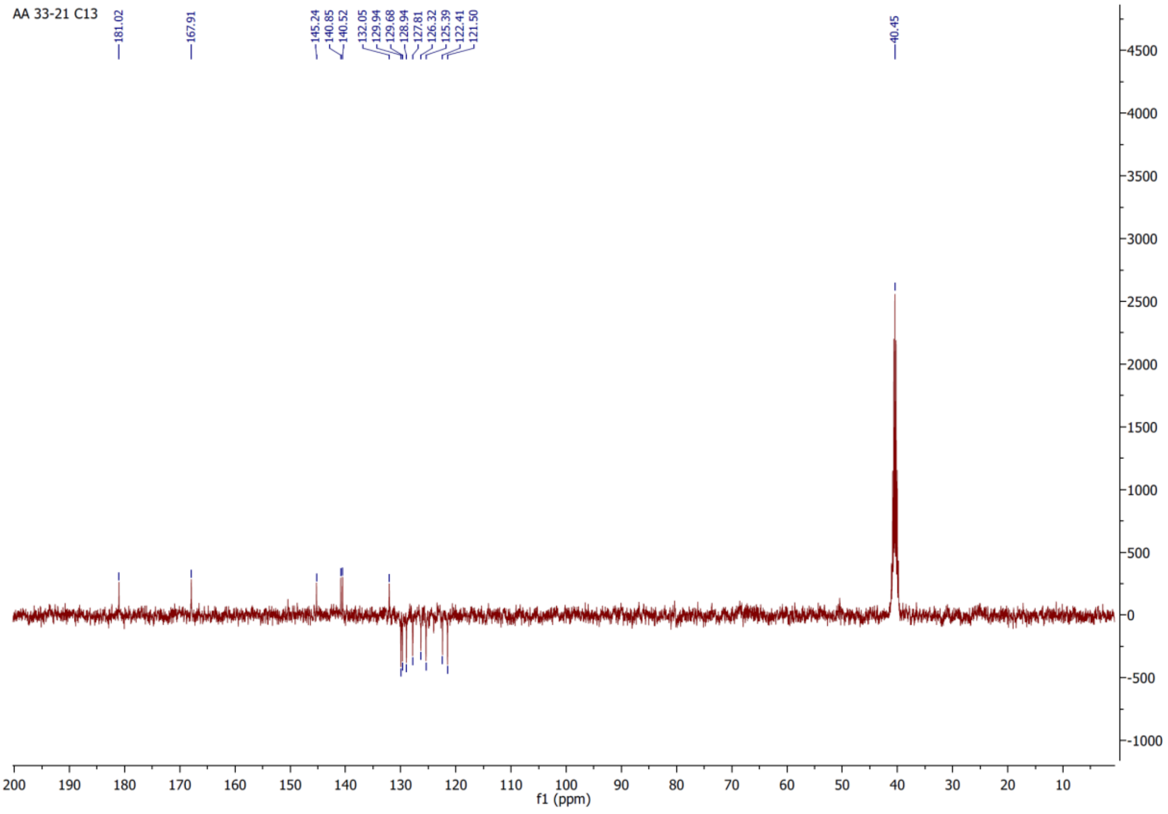

^{13}C NMR spectrum of compound **20b** (100 MHz, DMSO-d_6)

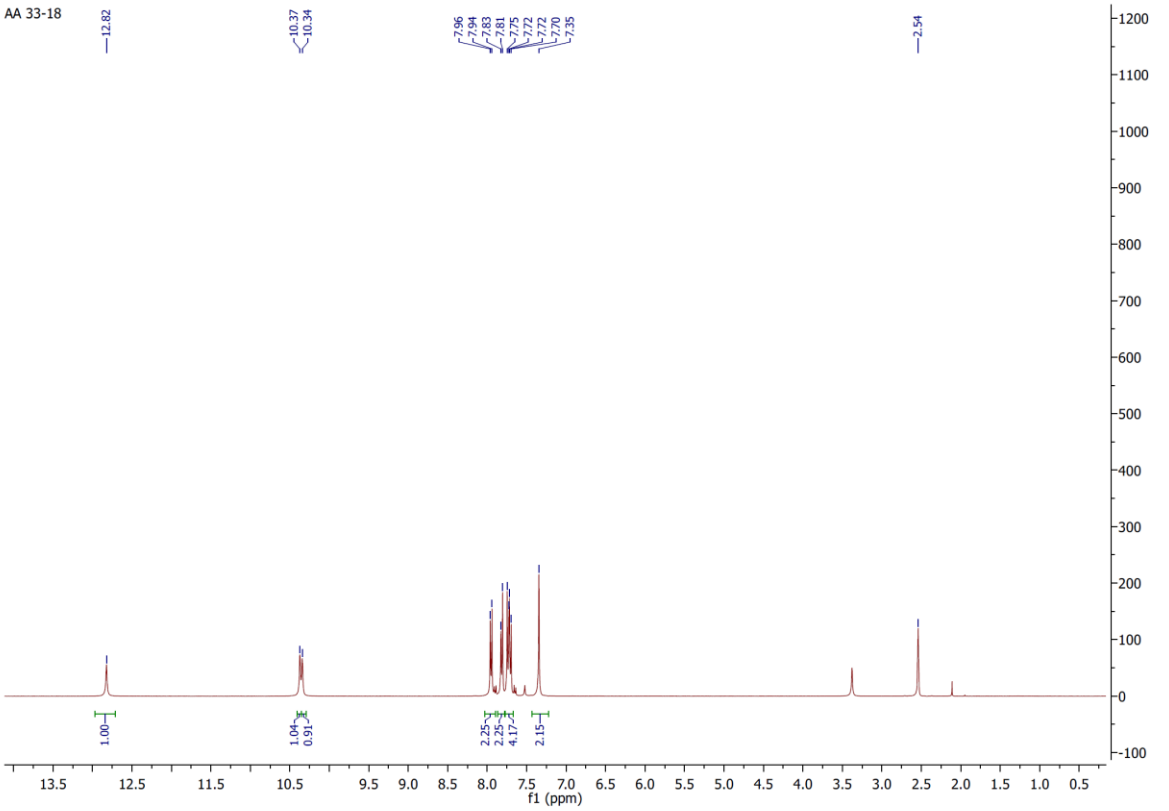

^{1}H NMR spectrum of compound **20c** (400 MHz, DMSO-d_6)

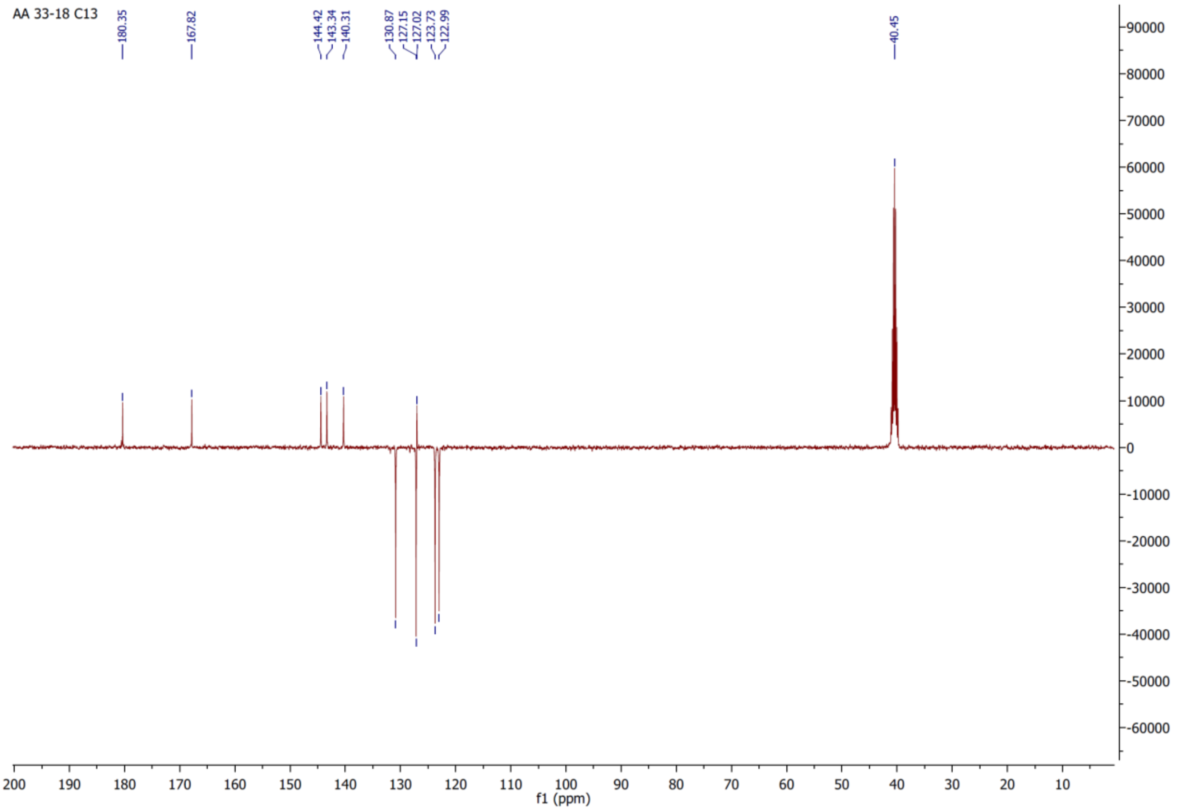

^{13}C NMR spectrum of compound **20c** (100 MHz, DMSO-d_6)

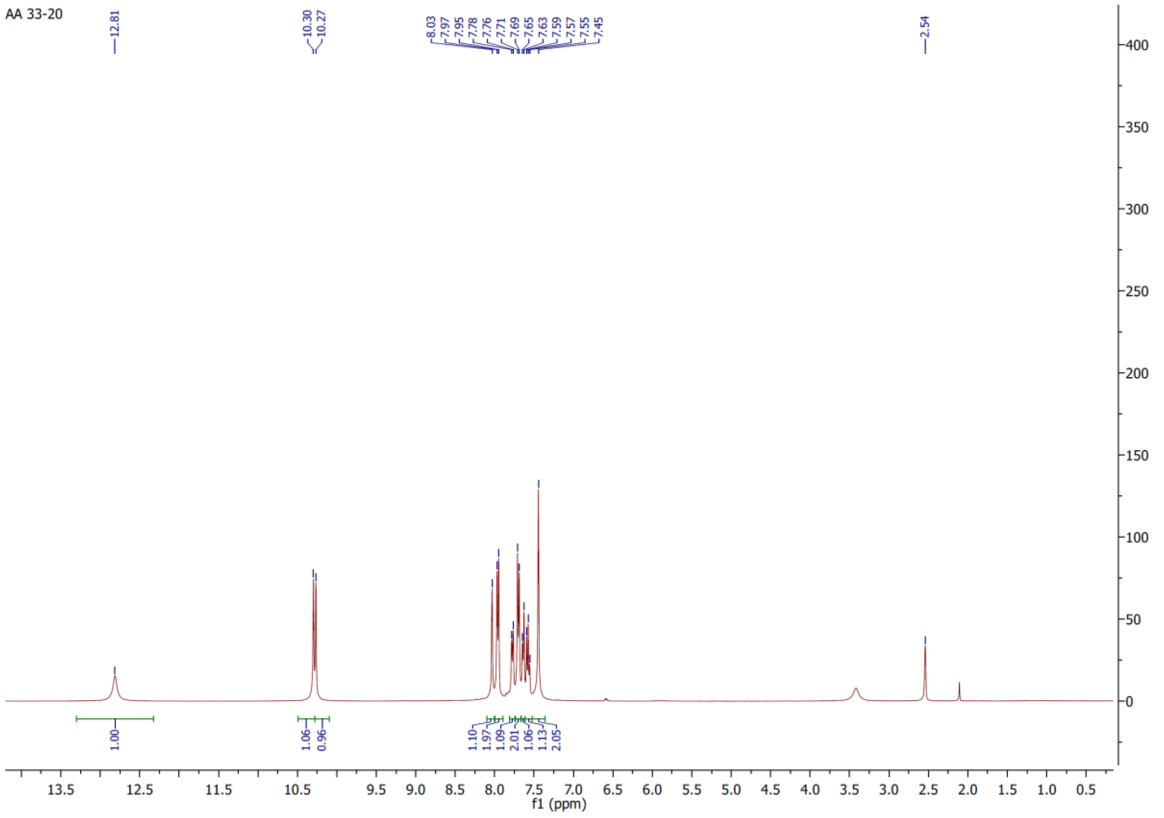

^{1}H NMR spectrum of compound **20d** (400 MHz, DMSO-d_6)

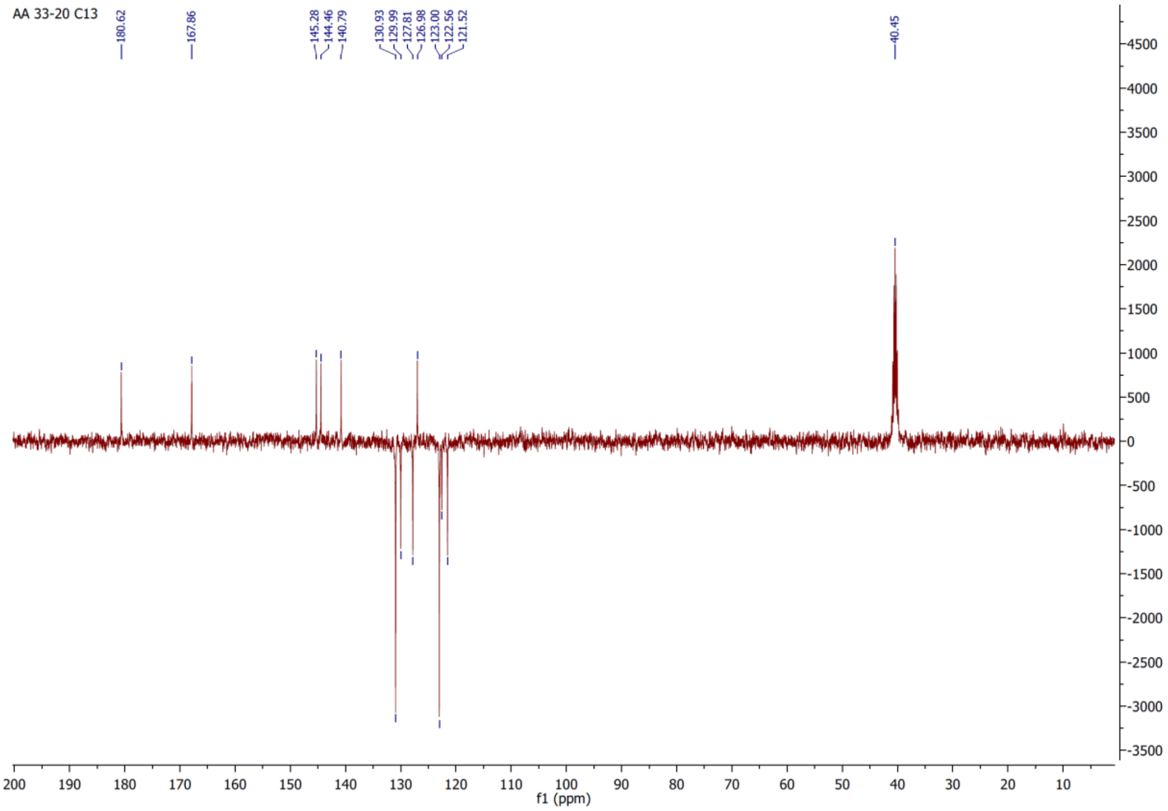

^{13}C NMR spectrum of compound **20d** (100 MHz, DMSO-d_6)

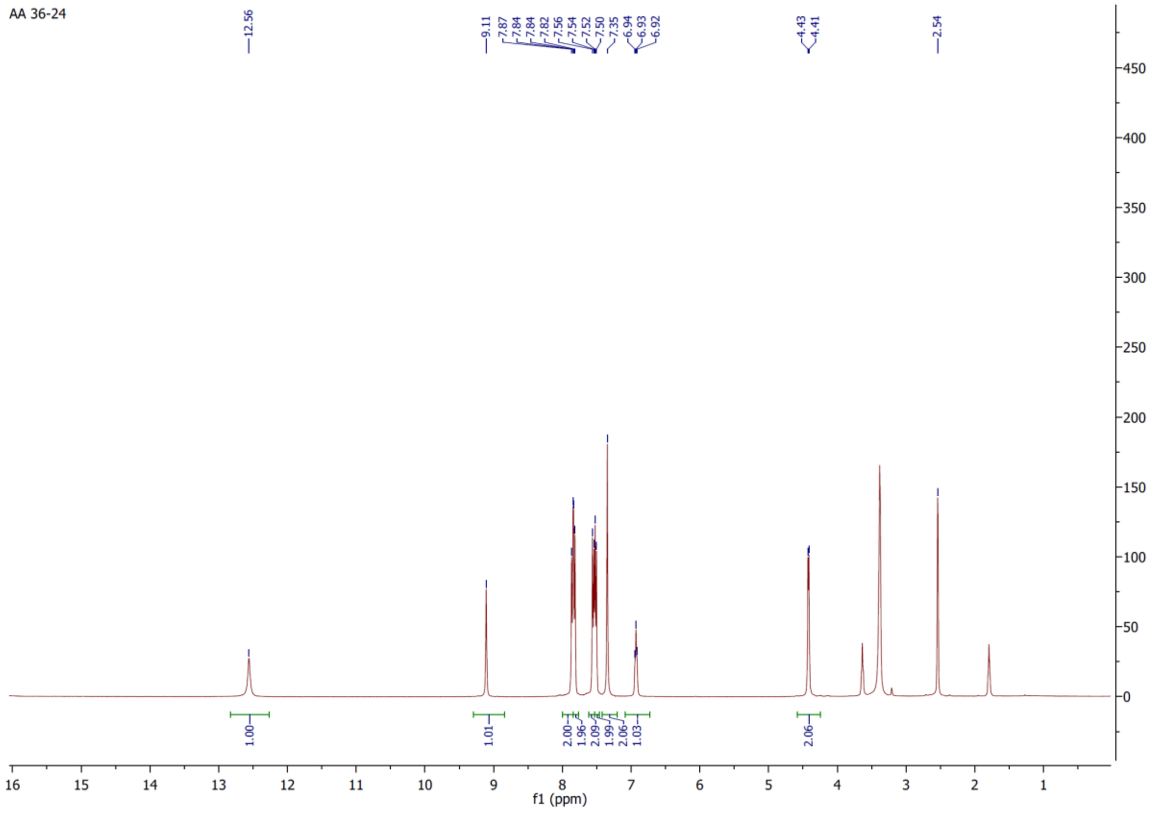

^{1}H NMR spectrum of compound **21** (400 MHz, DMSO-d_6)

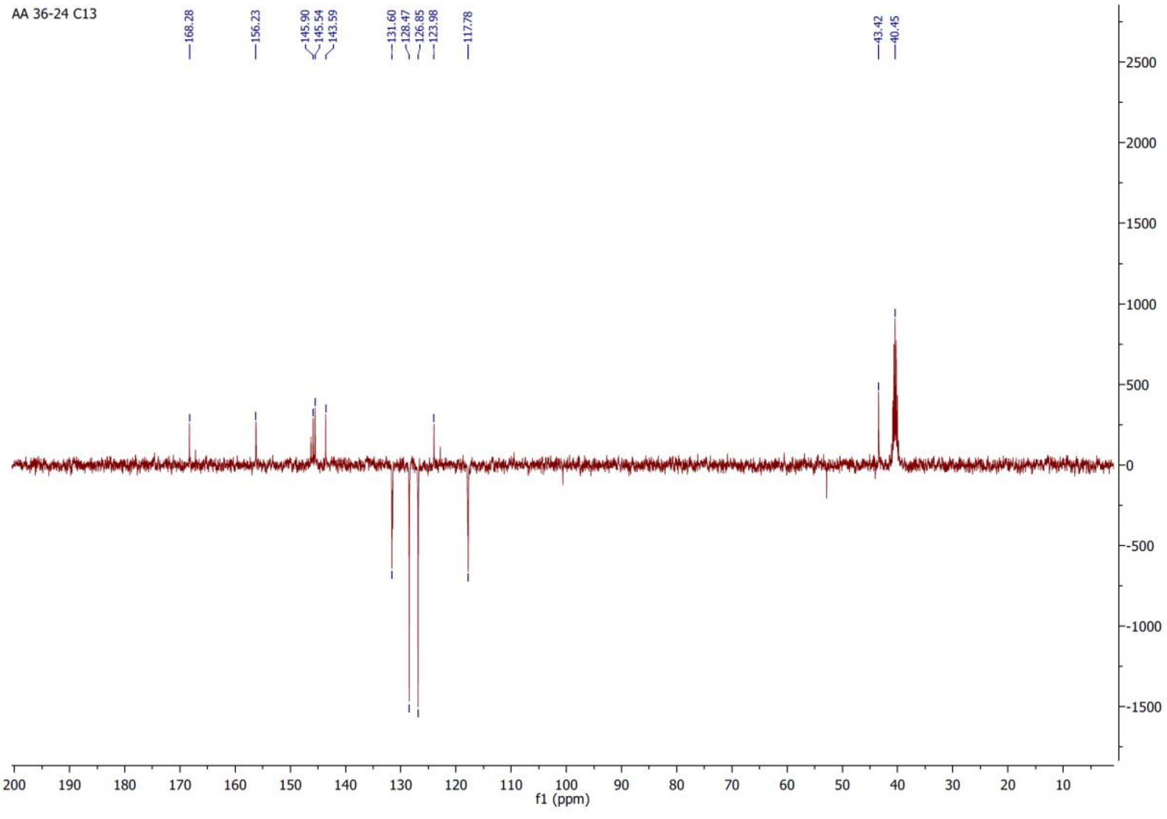

^{13}C NMR spectrum of compound **21** (100 MHz, DMSO-d_6)

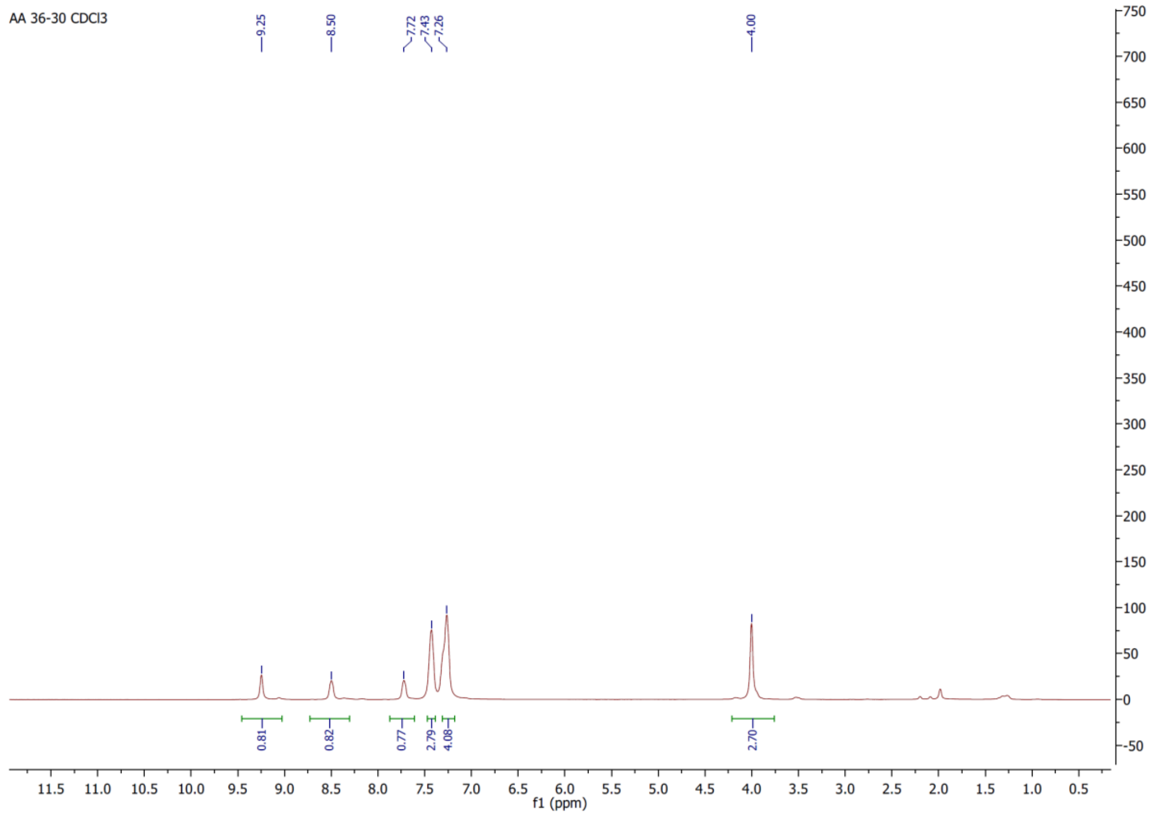

^{1}H NMR spectrum of compound **24** (400 MHz, $CDCl_3$)

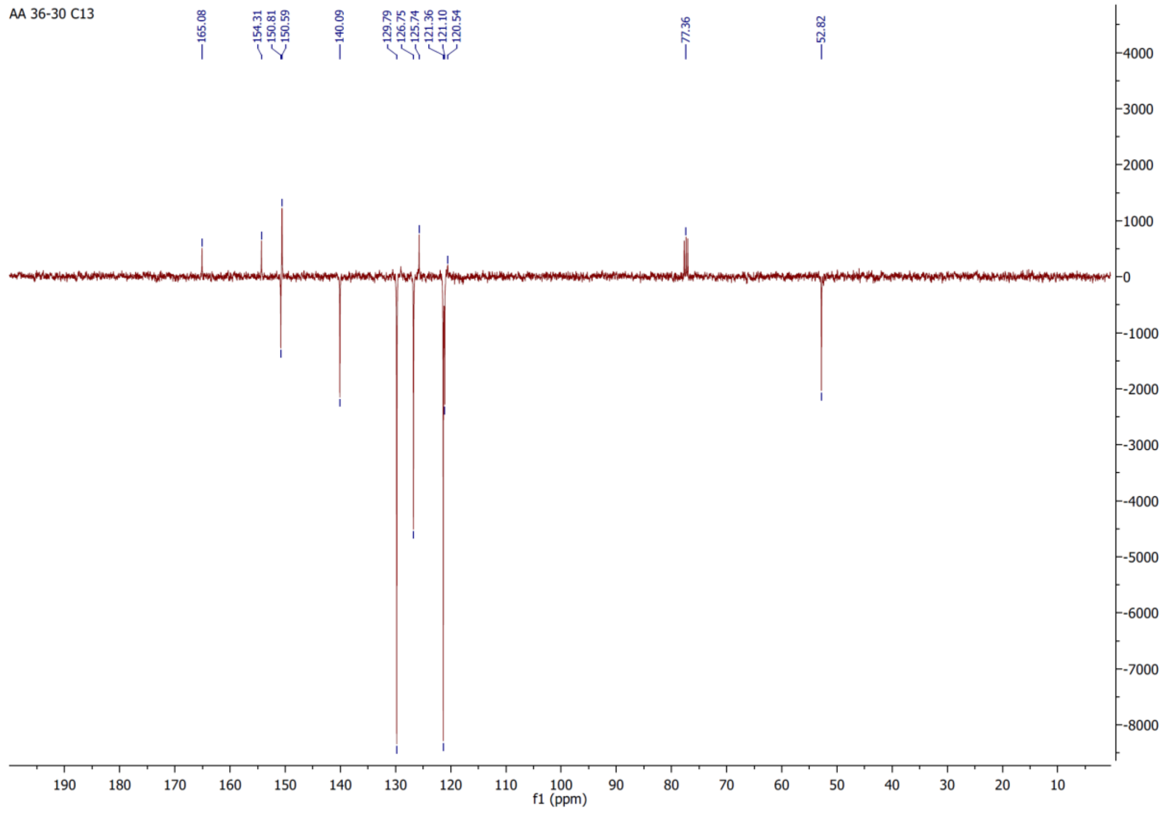

^{13}C NMR spectrum of compound **24** (100 MHz, $CDCl_3$)

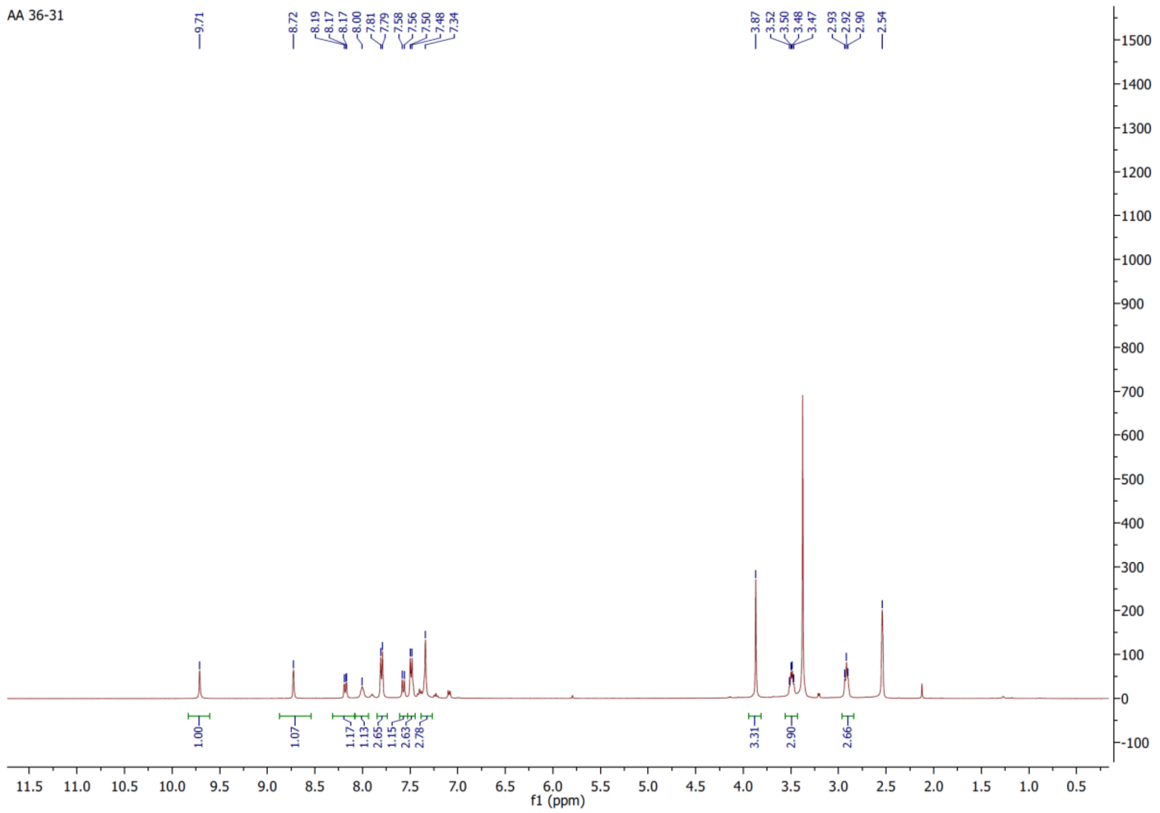

^{1}H NMR spectrum of compound **26** (400 MHz, DMSO-d_6)

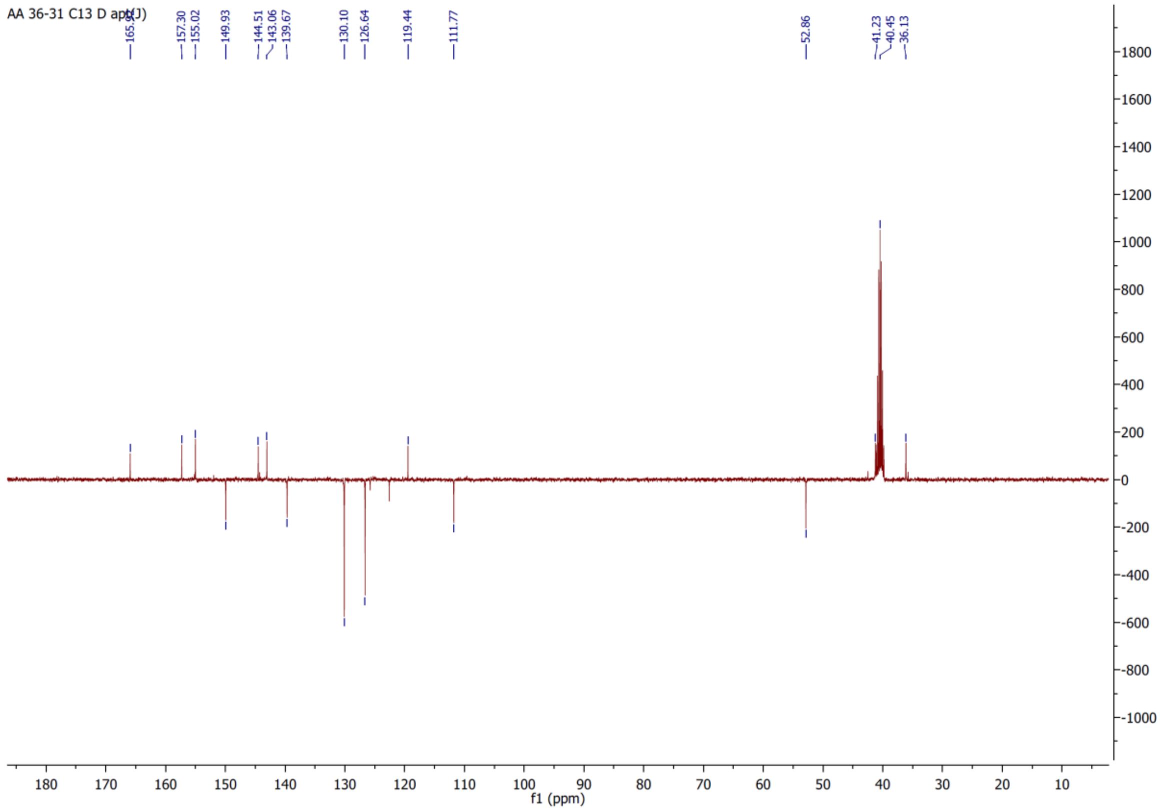

^{13}C NMR spectrum of compound **26** (100 MHz, DMSO-d_6)

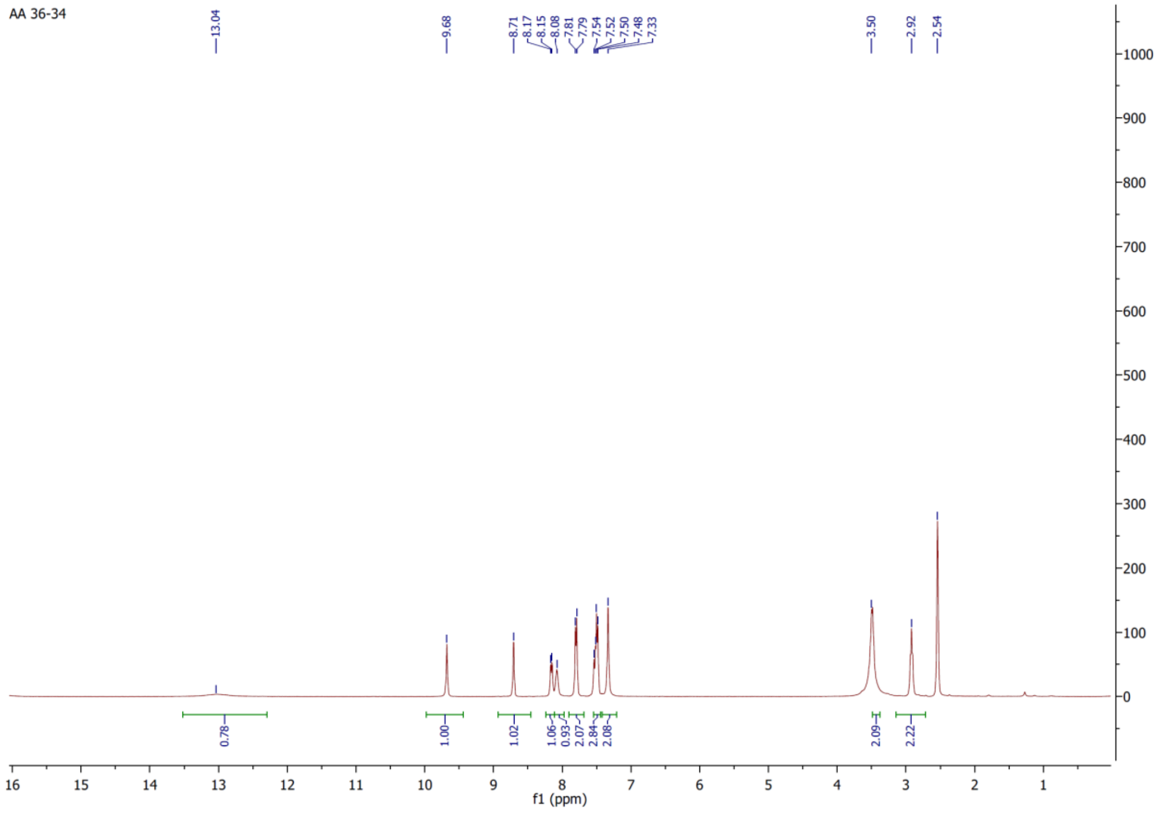

^{1}H NMR spectrum of compound **27** (400 MHz, DMSO-d_6)

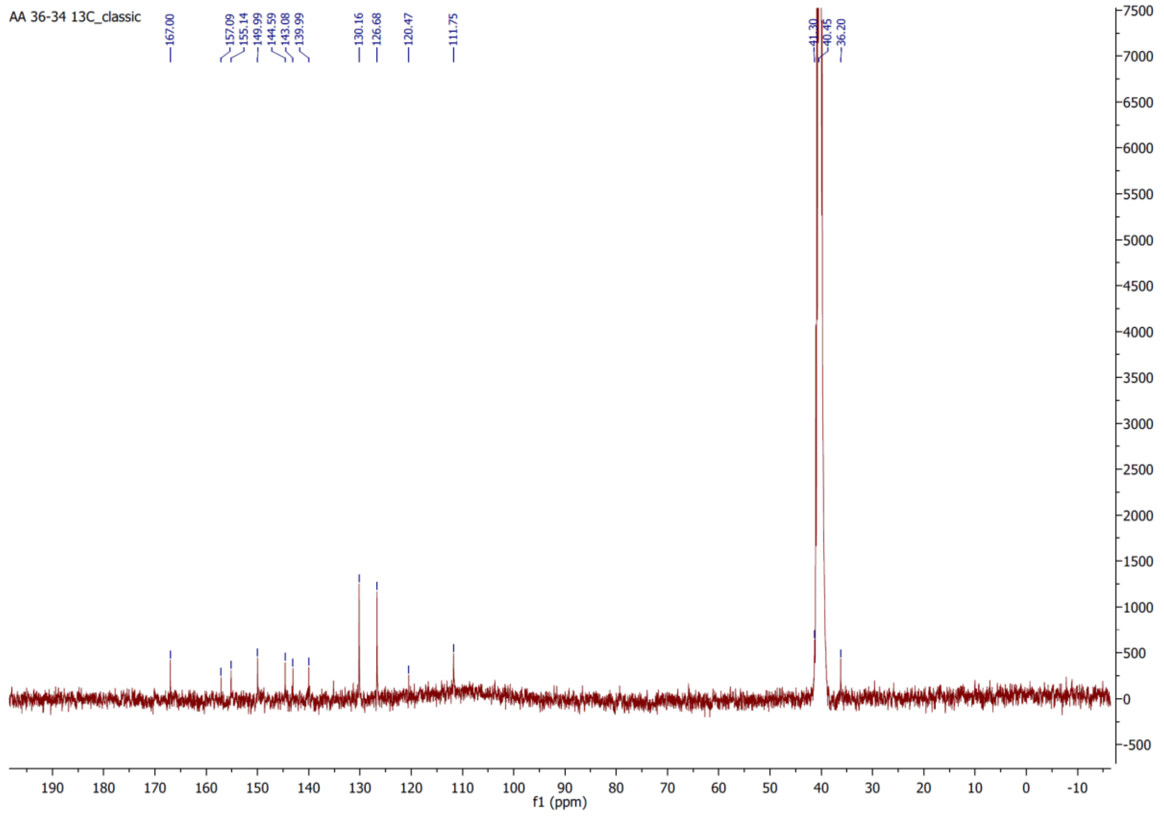

^{13}C NMR spectrum of compound **27** (100 MHz, DMSO-d_6)

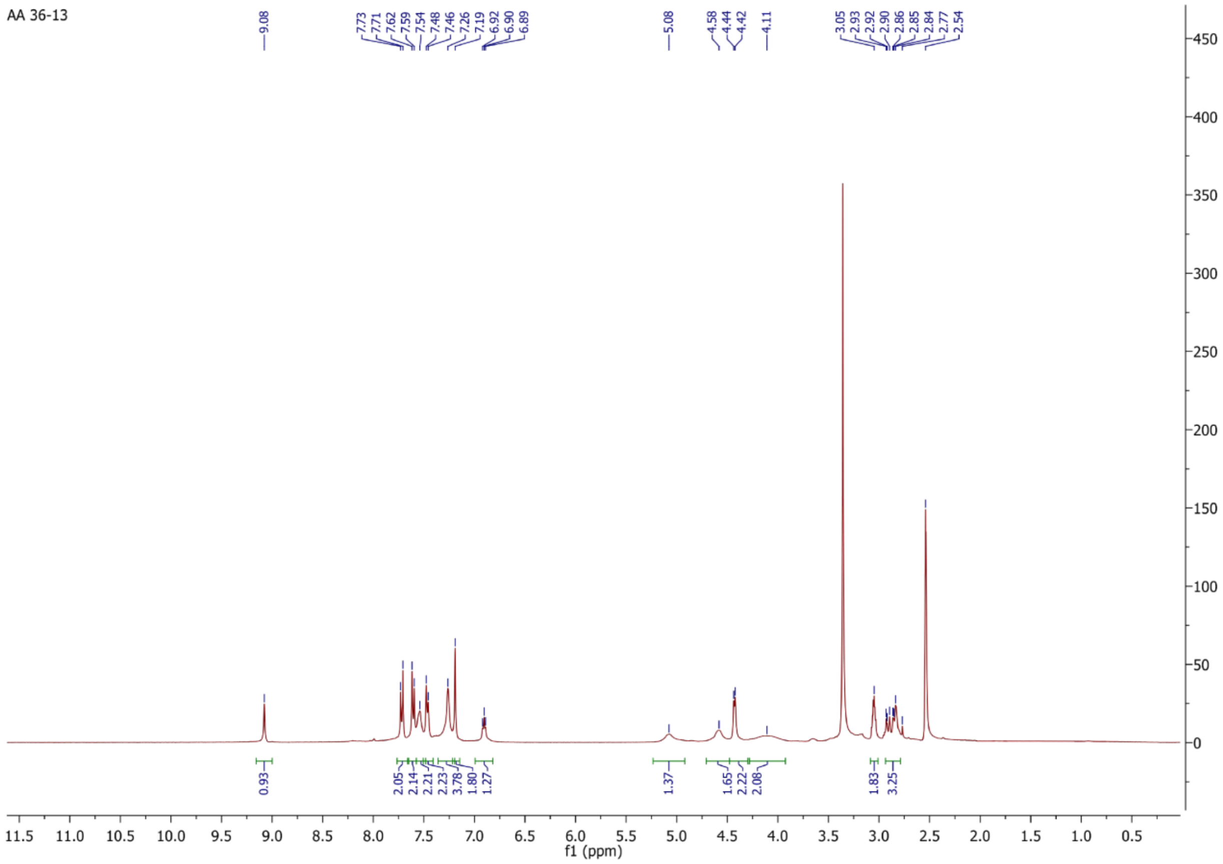

^{1}H NMR spectrum of compound **28a** (400 MHz, DMSO-d_6)

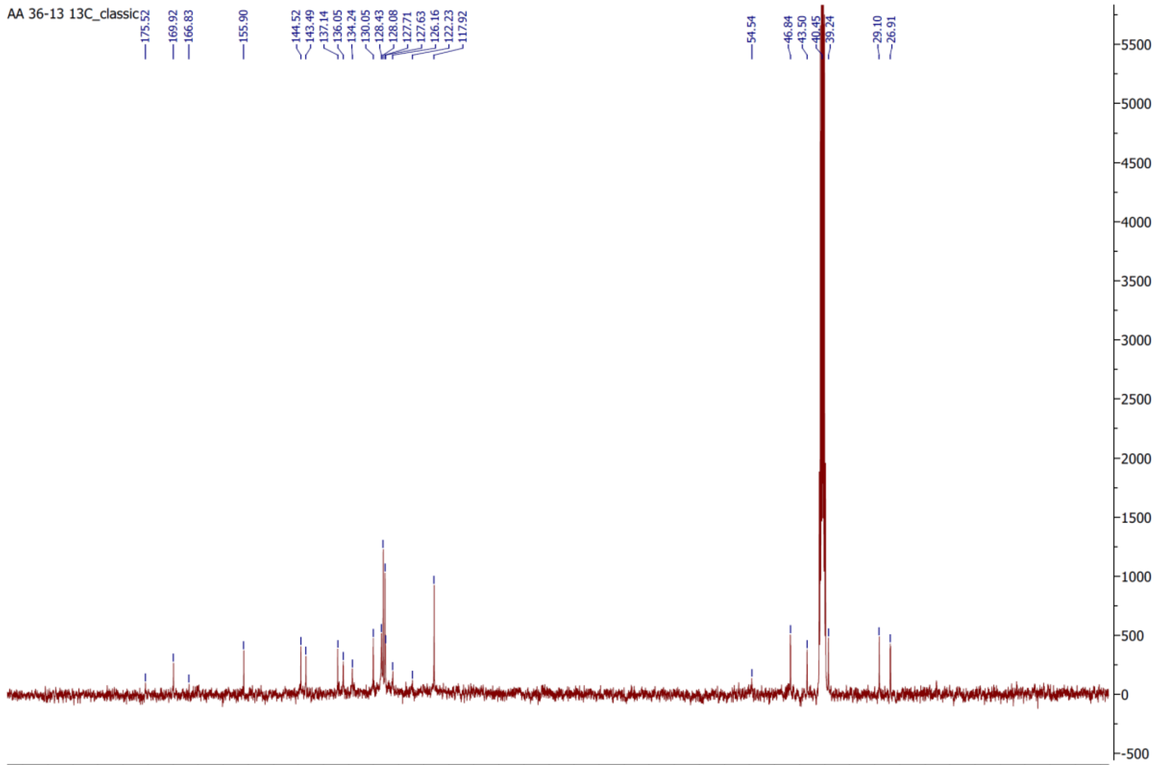

^{13}C NMR spectrum of compound **28a** (100 MHz, DMSO-d_6)

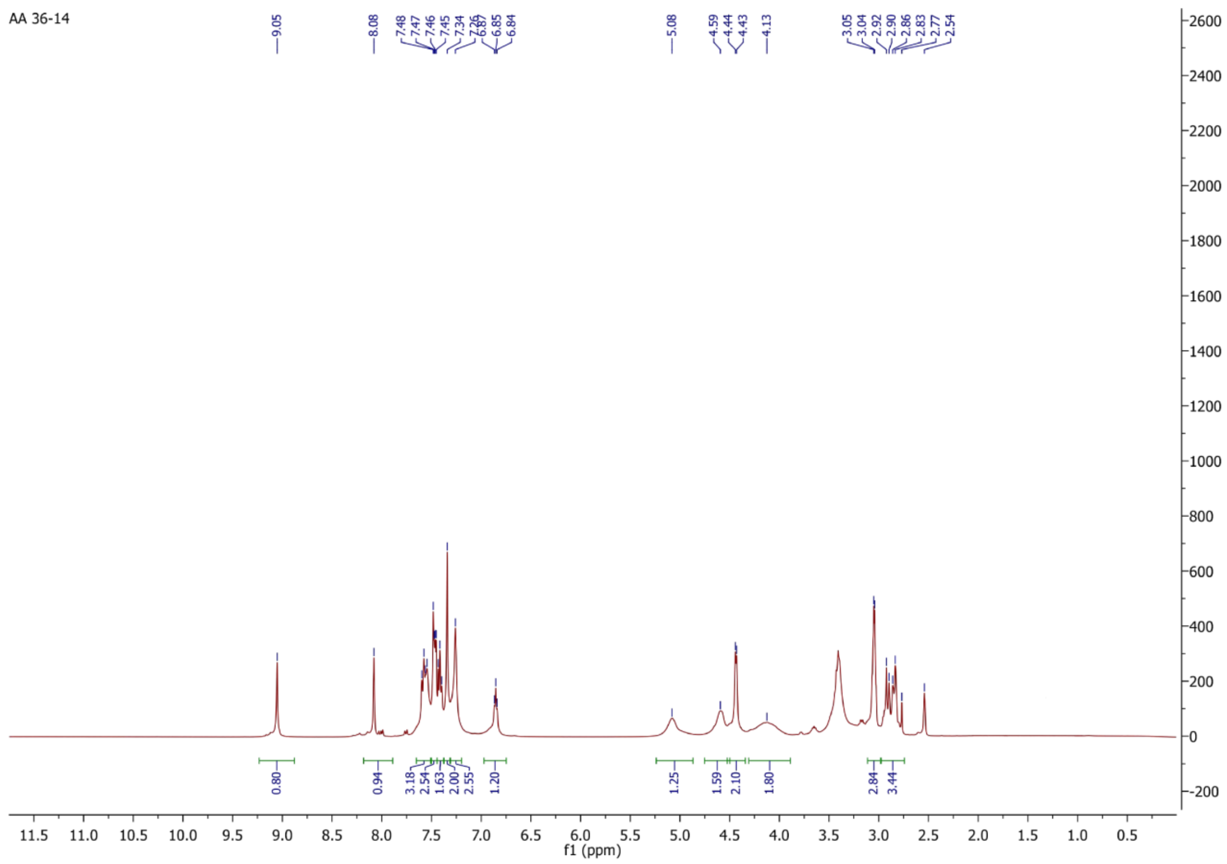

^{1}H NMR spectrum of compound **28b** (400 MHz, DMSO-d_6)

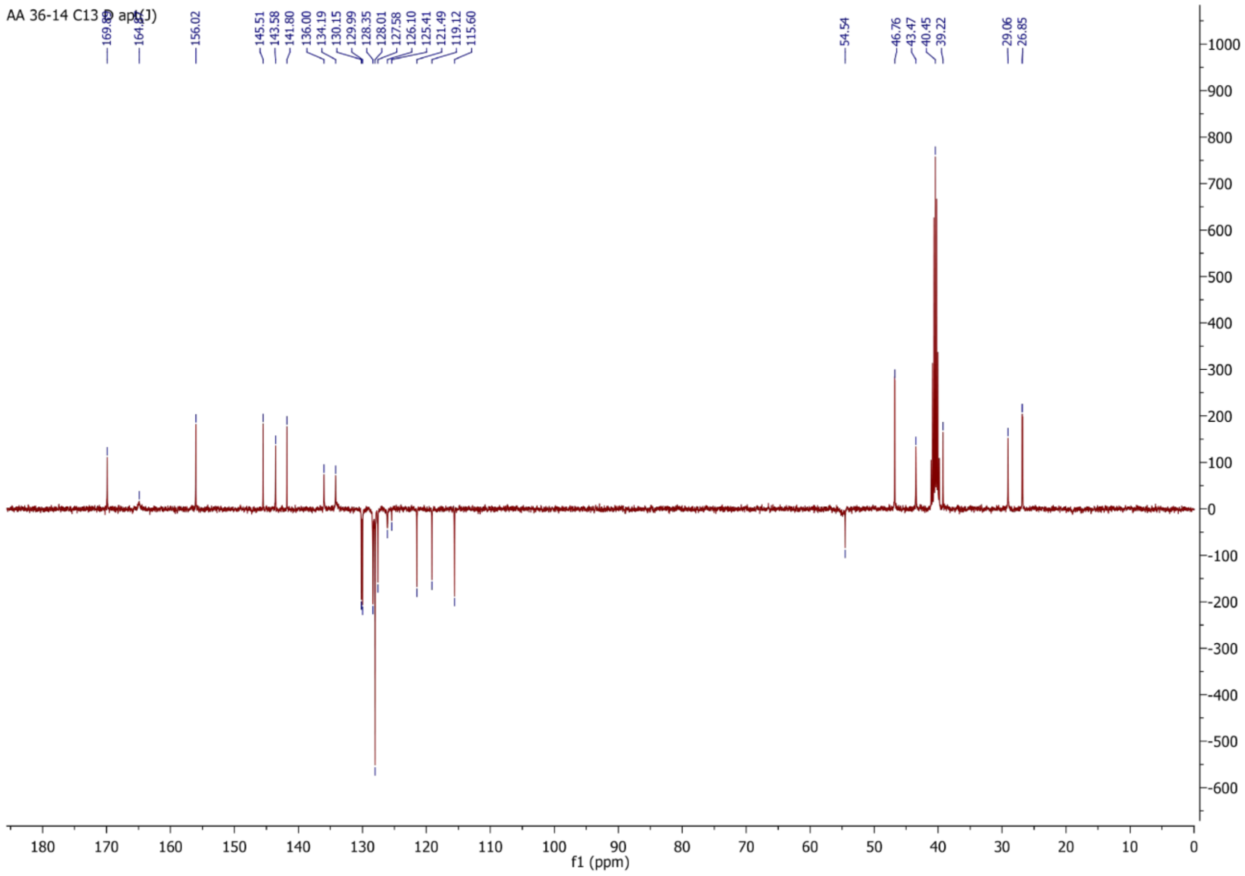

^{13}C NMR spectrum of compound **28b** (100 MHz, DMSO-d_6)

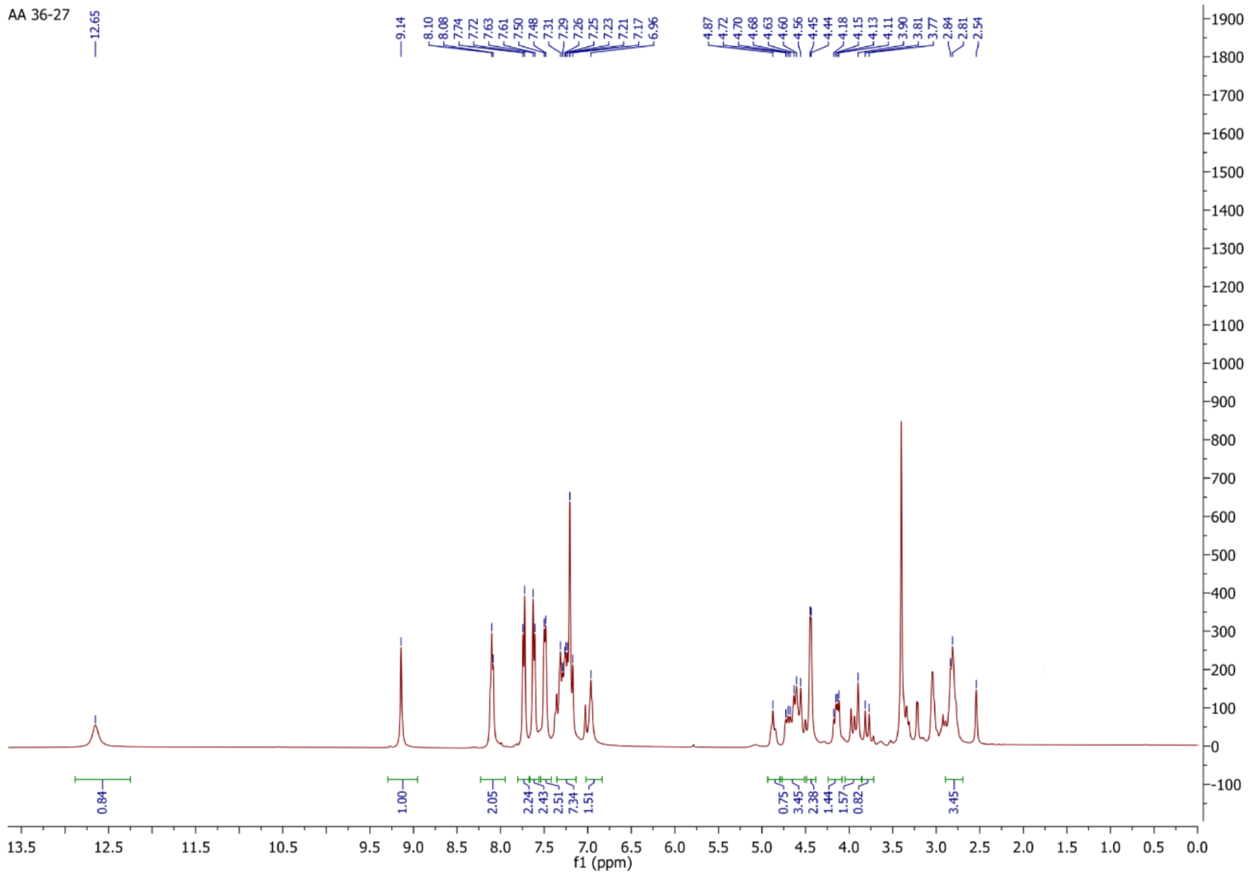

^{1}H NMR spectrum of compound **29a** (400 MHz, DMSO-d_6)

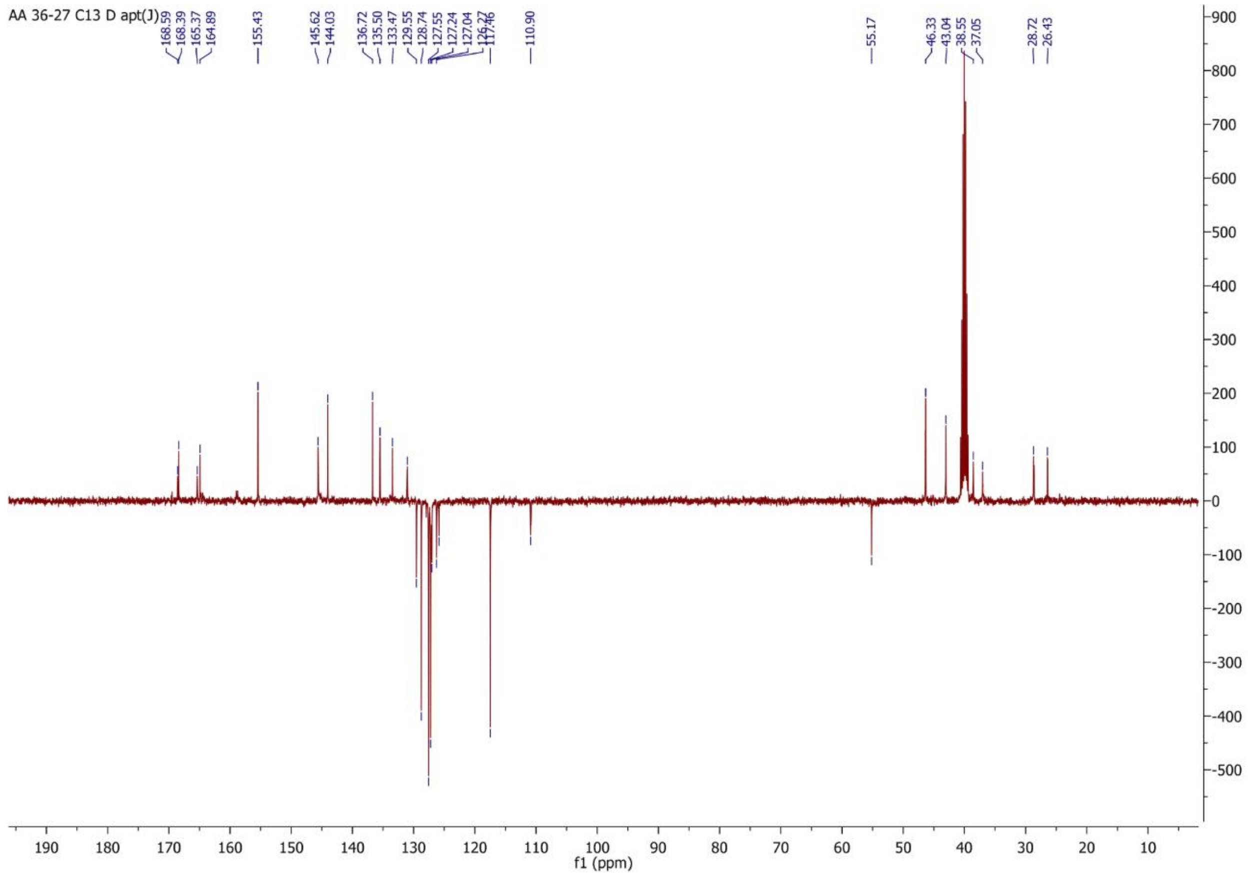

^{13}C NMR spectrum of compound **29a** (100 MHz, DMSO-d_6)

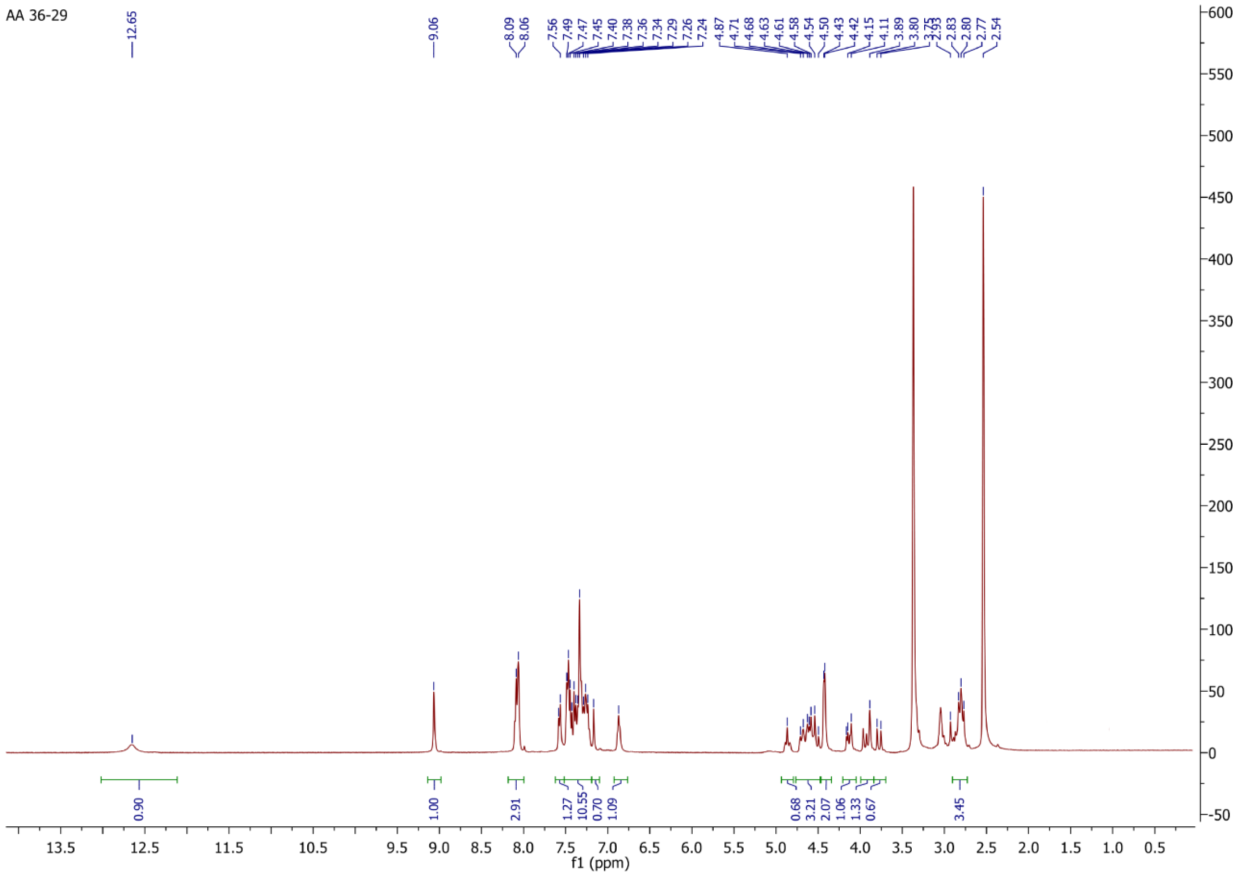

^{1}H NMR spectrum of compound **29b** (400 MHz, DMSO-d_6)

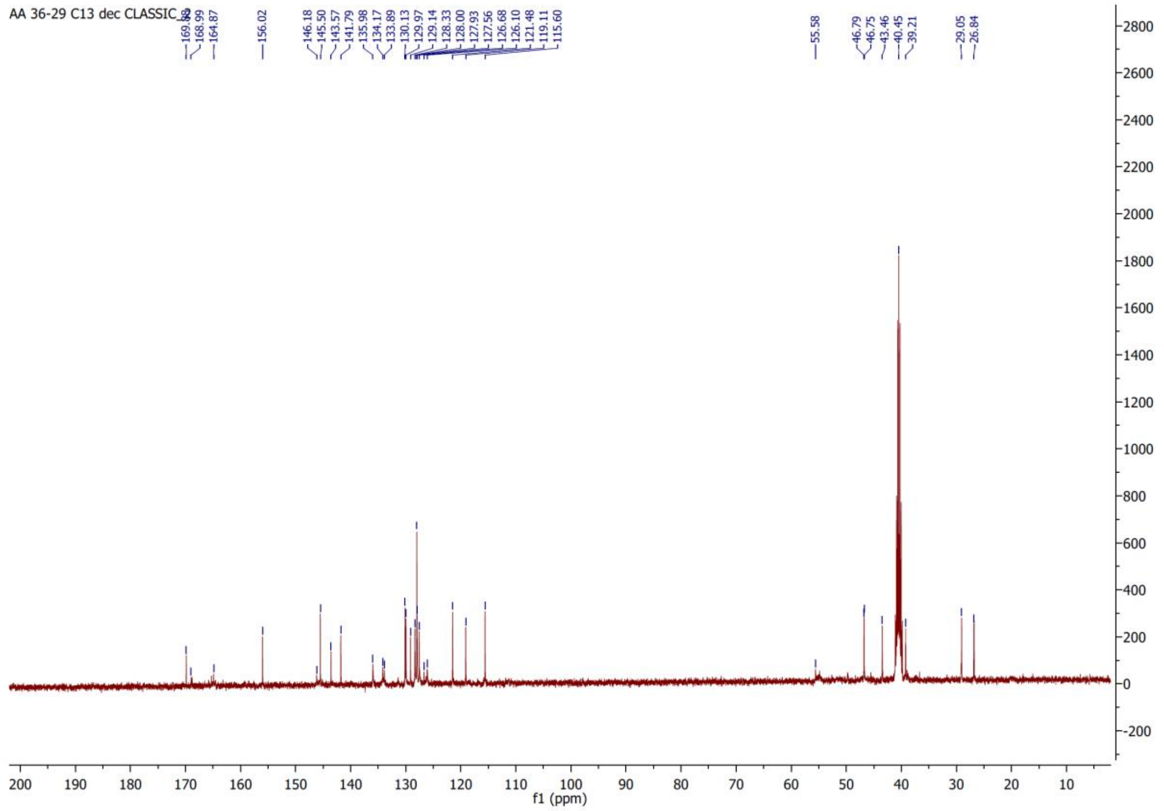

^{13}C NMR spectrum of compound **29b** (100 MHz, DMSO-d_6)

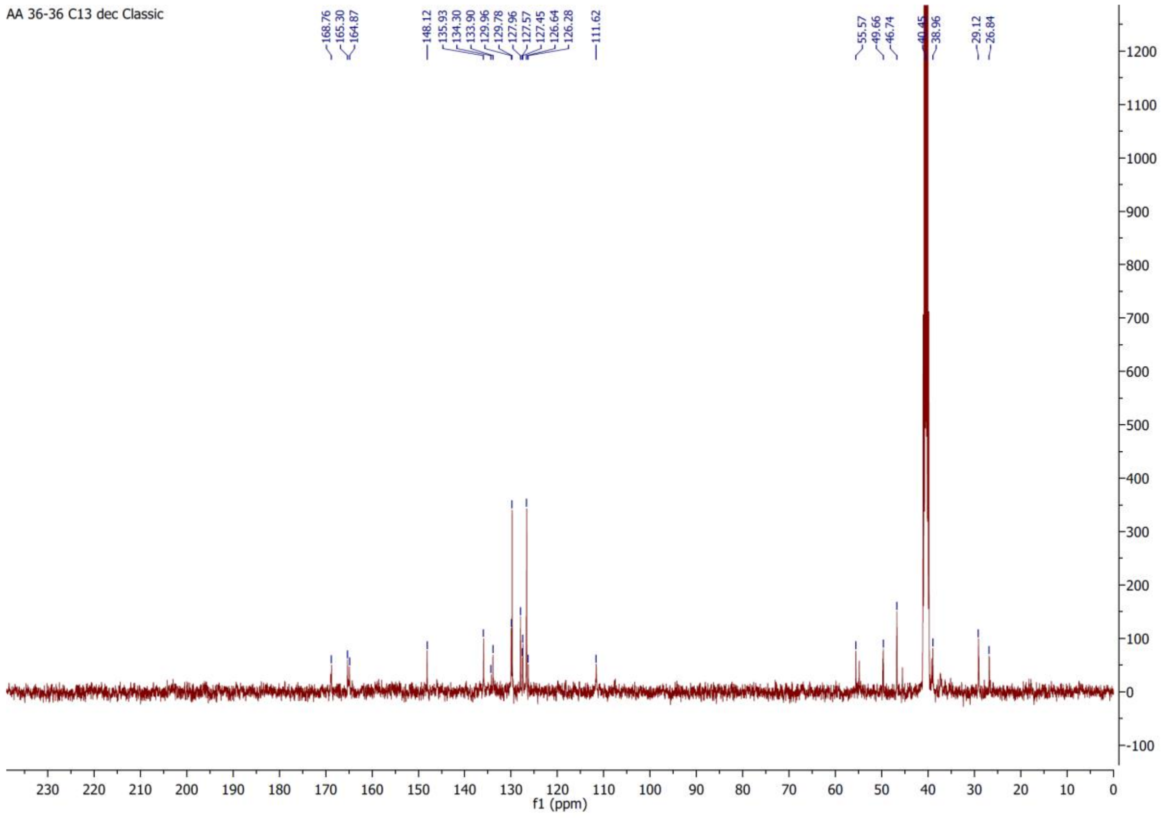

^{1}H NMR spectrum of compound **30** (400 MHz, DMSO-d_6)

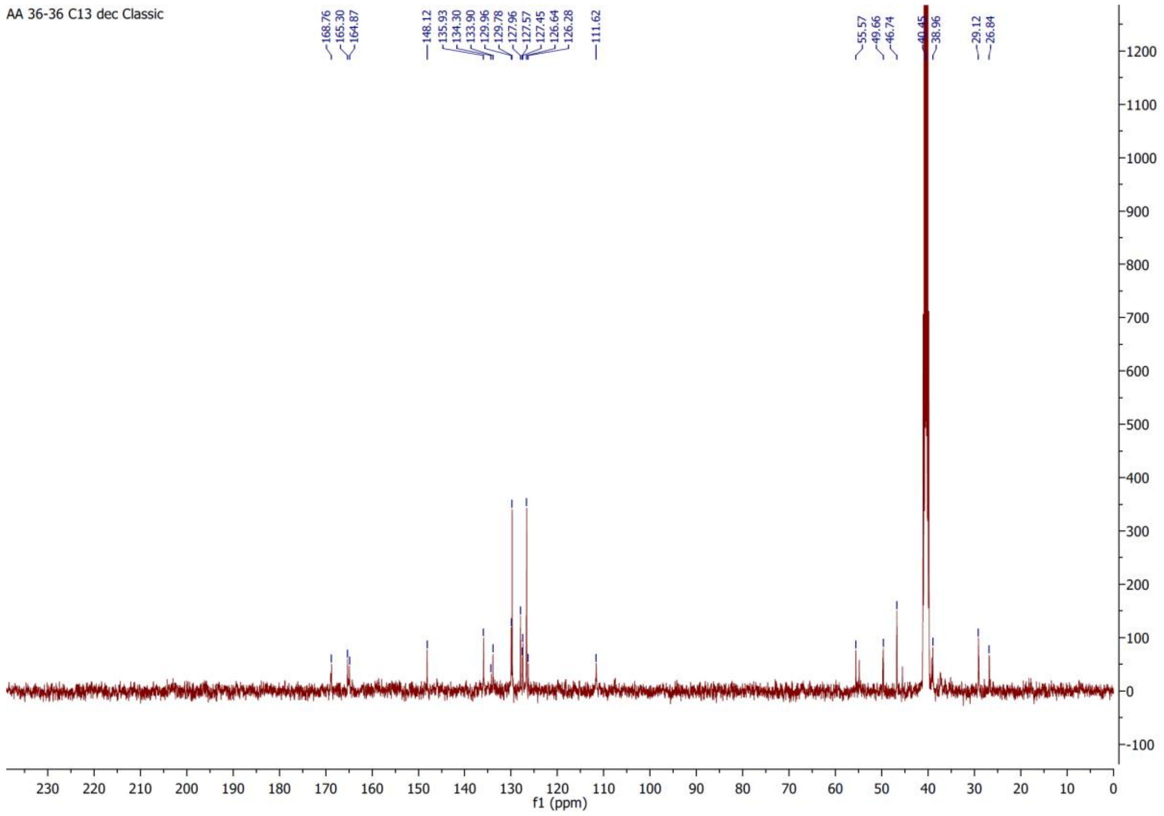

^{13}C NMR spectrum of compound **30** (100 MHz, DMSO-d_6)

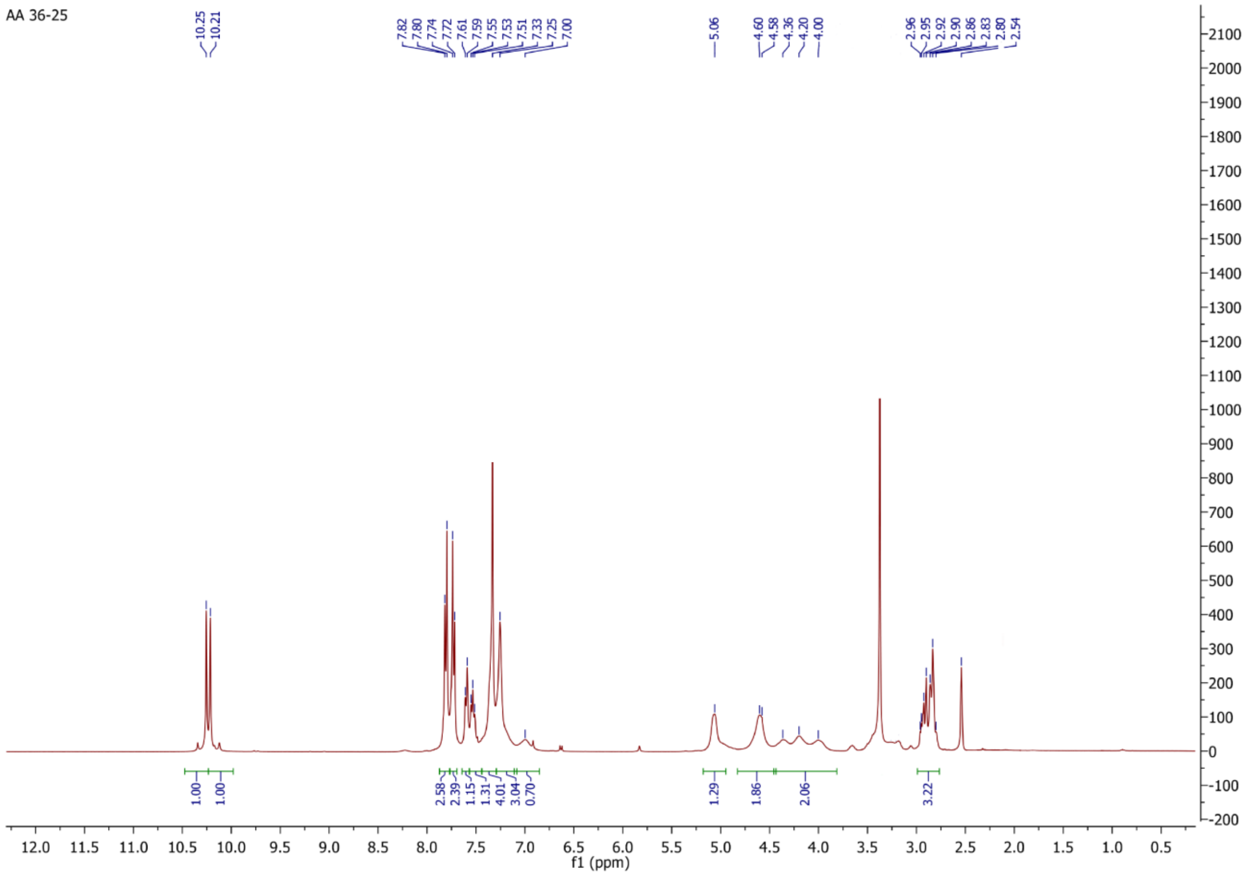

^{1}H NMR spectrum of compound **31a** (400 MHz, DMSO-d_6)

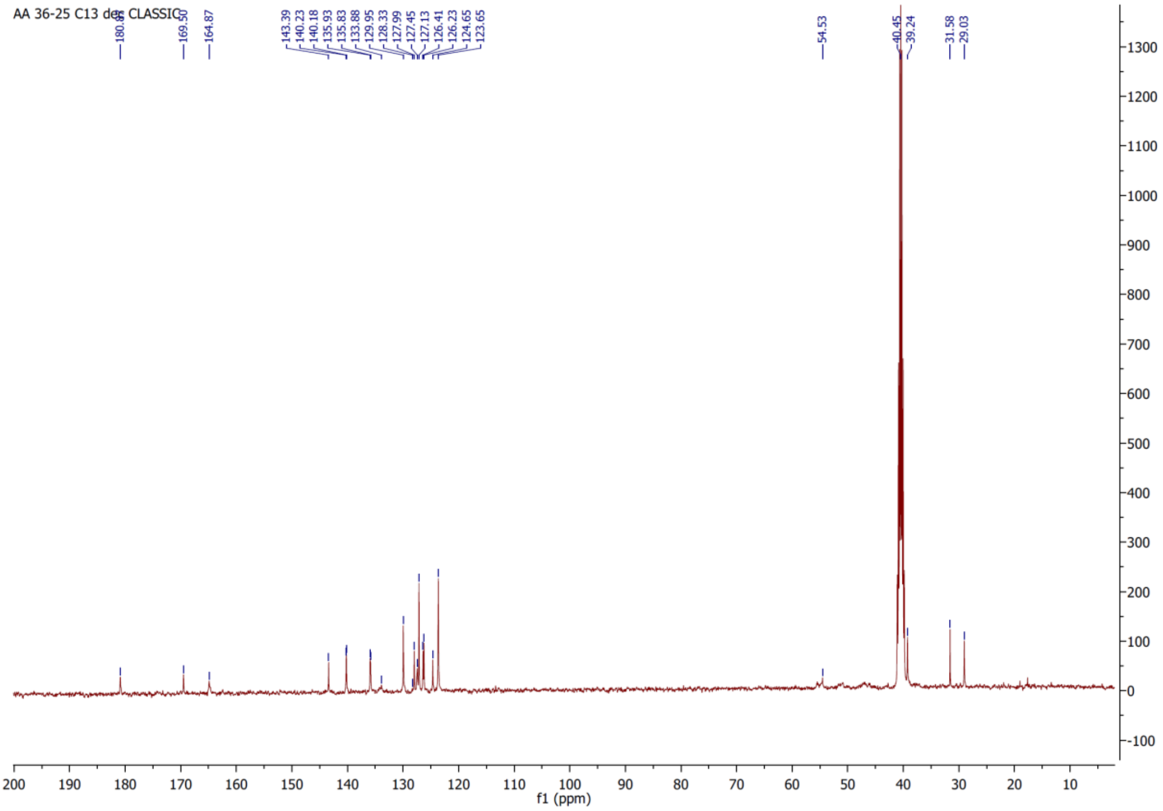

^{13}C NMR spectrum of compound **31a** (100 MHz, DMSO-d_6)

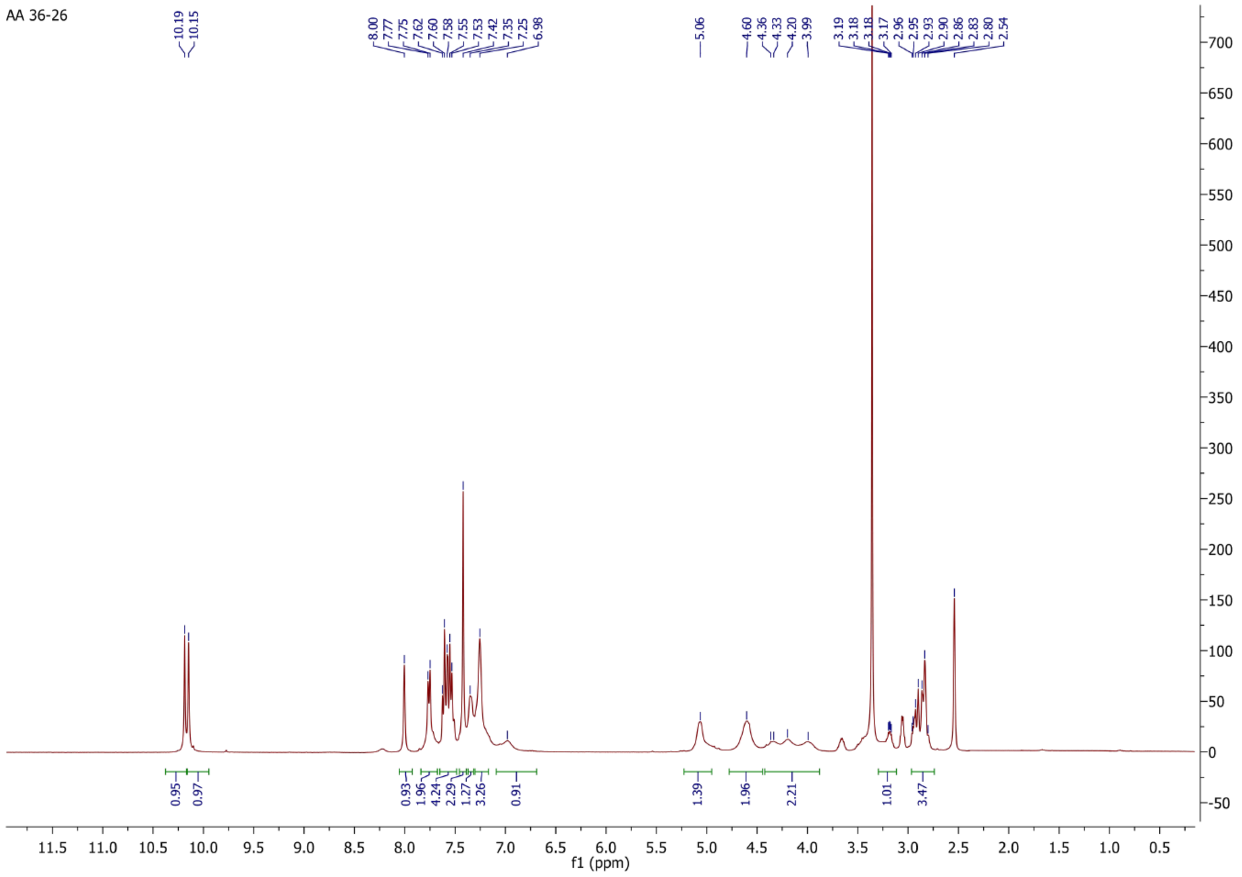

^{1}H NMR spectrum of compound **31b** (400 MHz, DMSO-d_6)

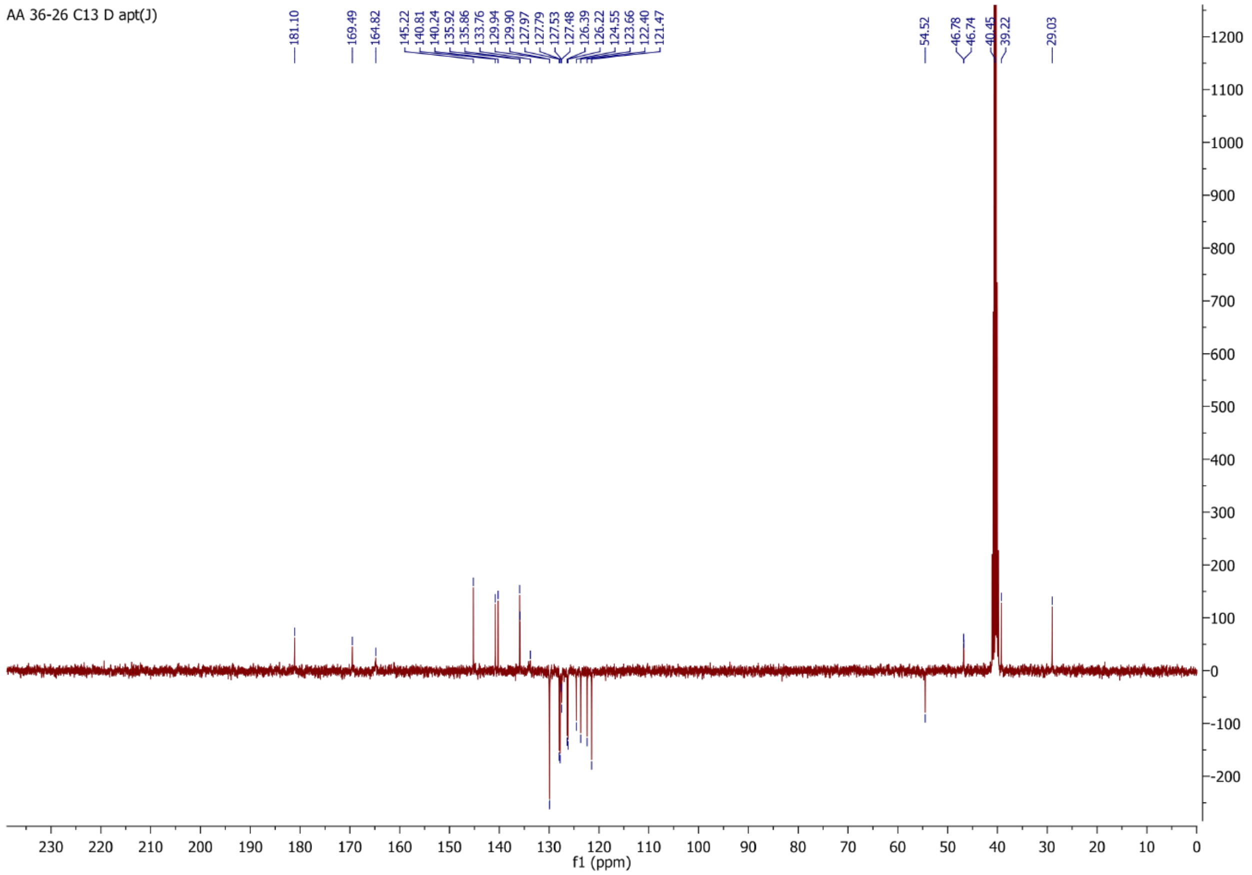

^{13}C NMR spectrum of compound **31b** (100 MHz, DMSO-d_6)

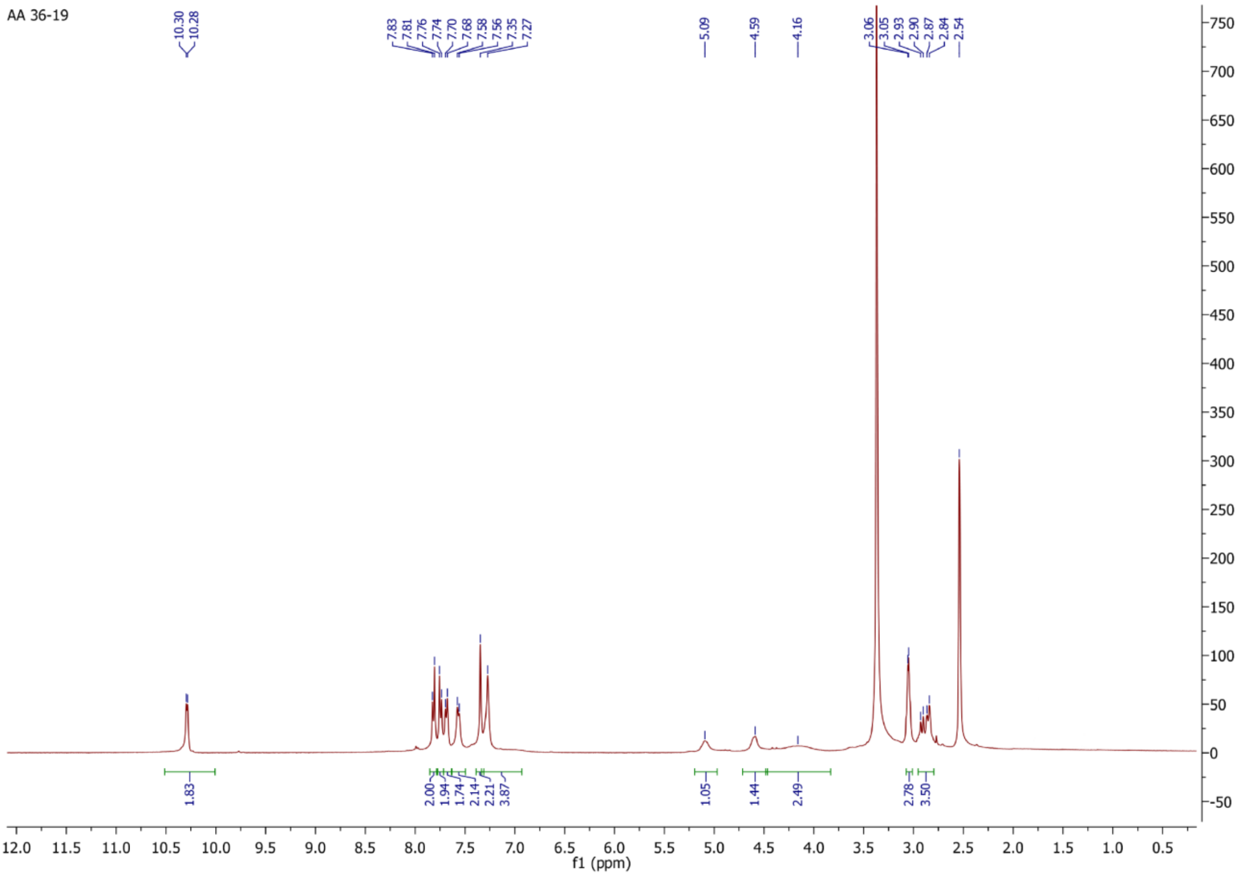

^{1}H NMR spectrum of compound **31c** (400 MHz, DMSO-d_6)

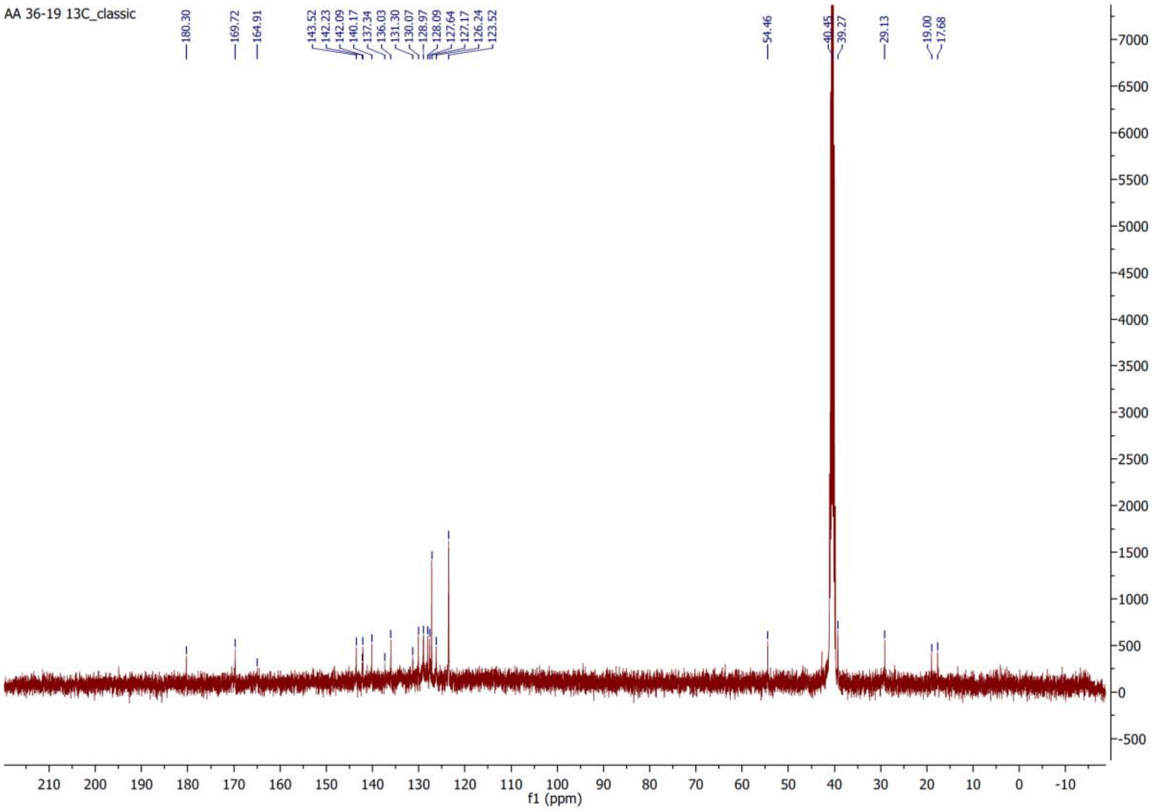

^{13}C NMR spectrum of compound **31c** (100 MHz, DMSO-d_6)

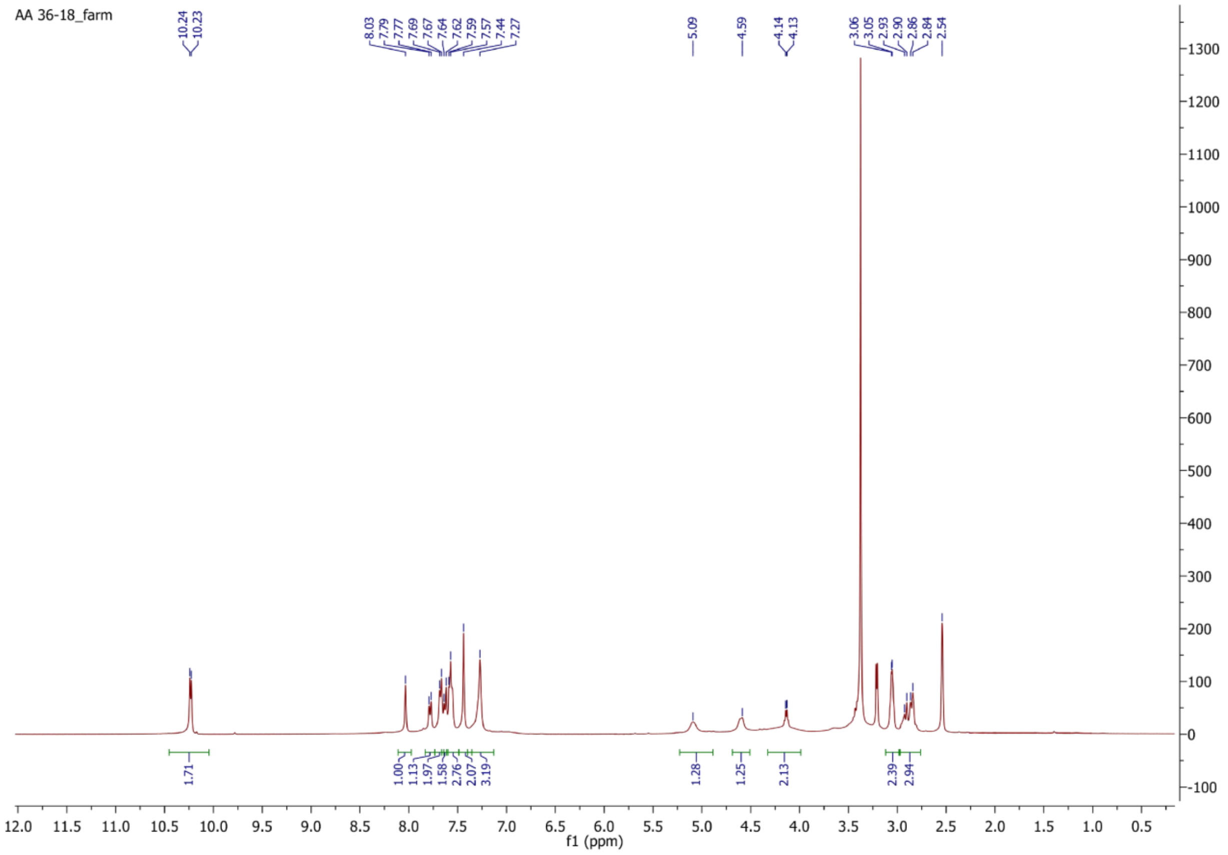

^{1}H NMR spectrum of compound **31d** (400 MHz, DMSO-d_6)

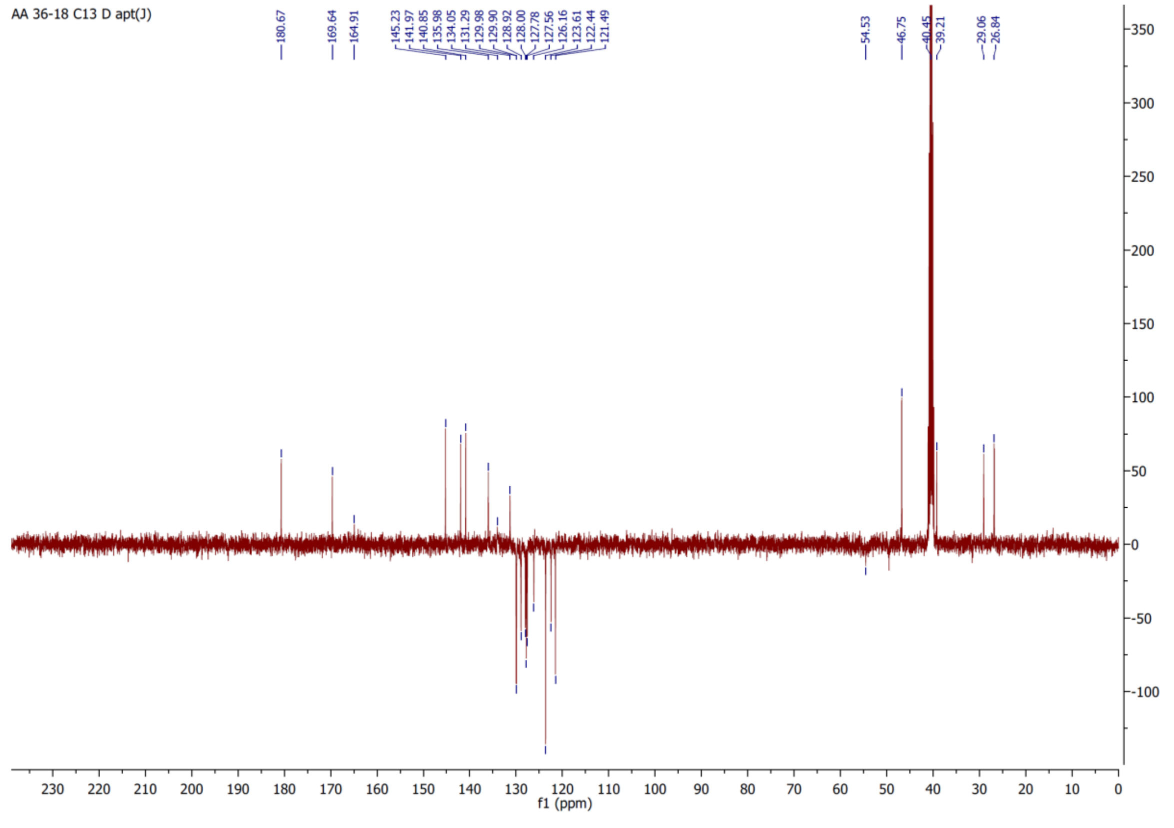

^{13}C NMR spectrum of compound **31d** (100 MHz, DMSO-d_6)

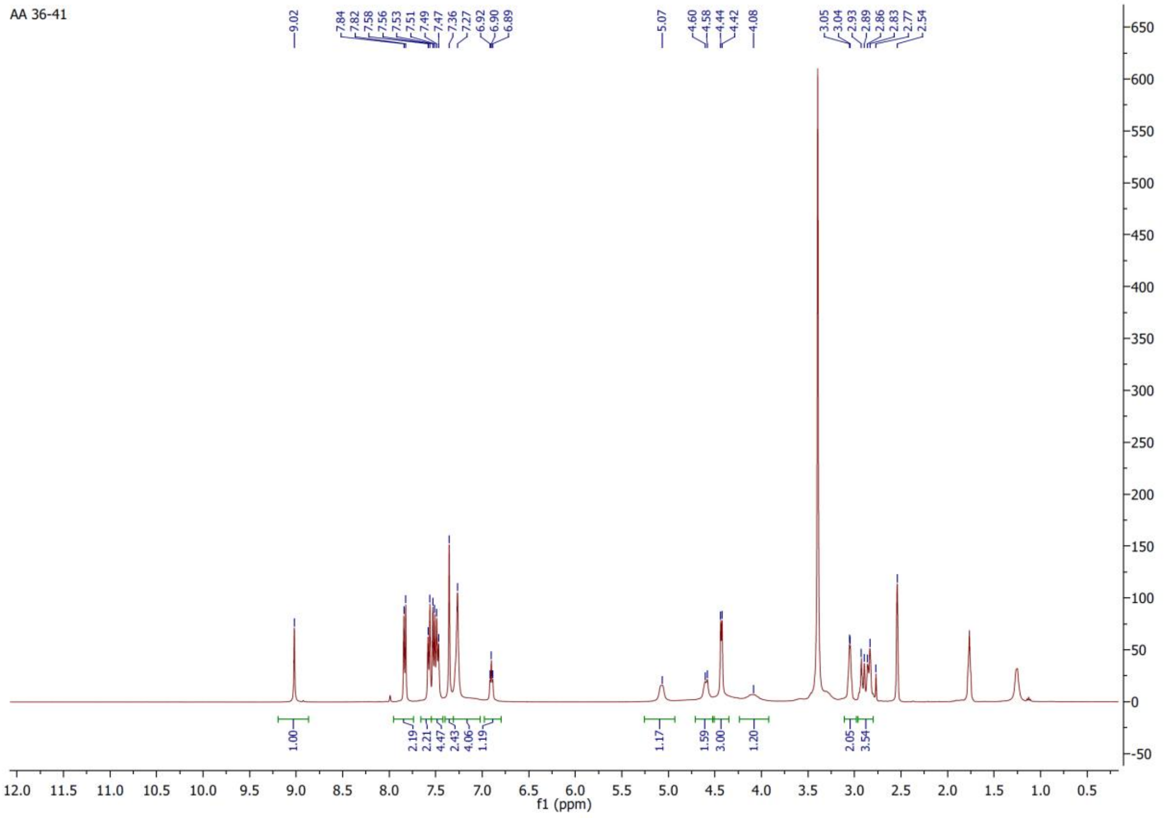

^{1}H NMR spectrum of compound **32** (400 MHz, DMSO-d_6)

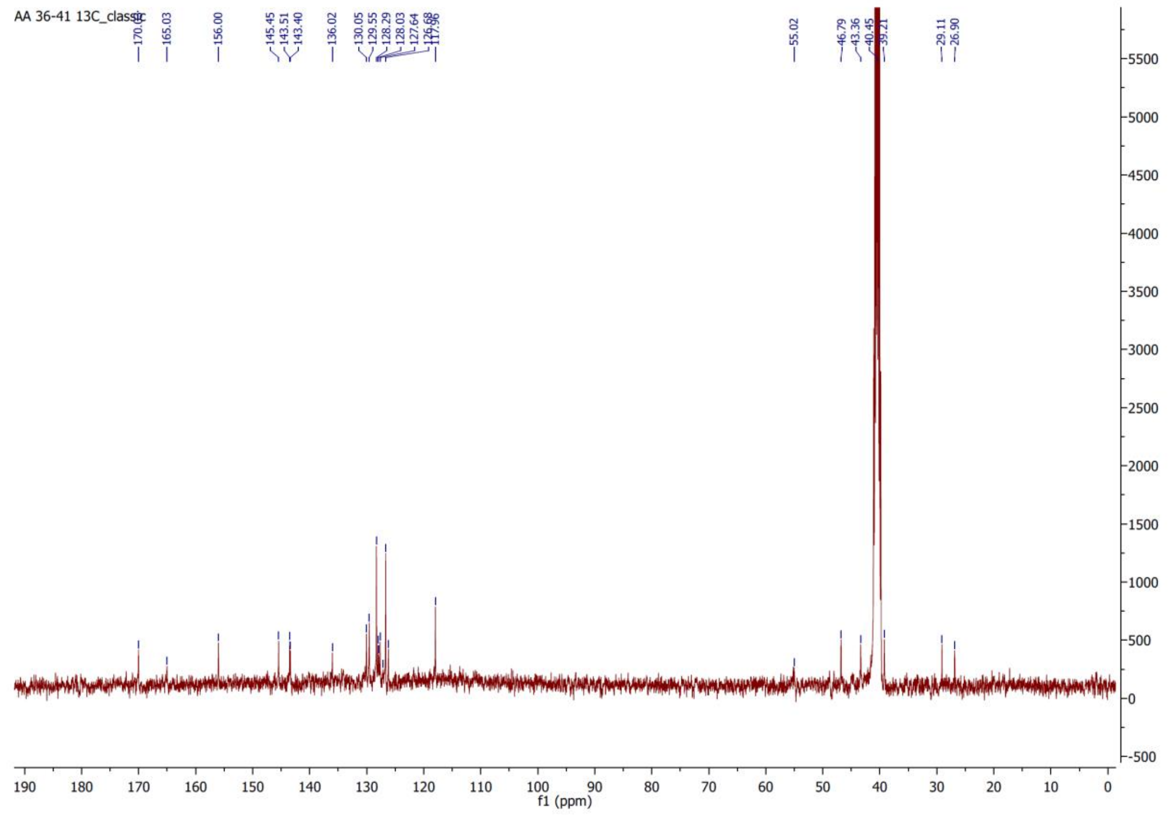

^{13}C NMR spectrum of compound **32** (100 MHz, DMSO-d_6)

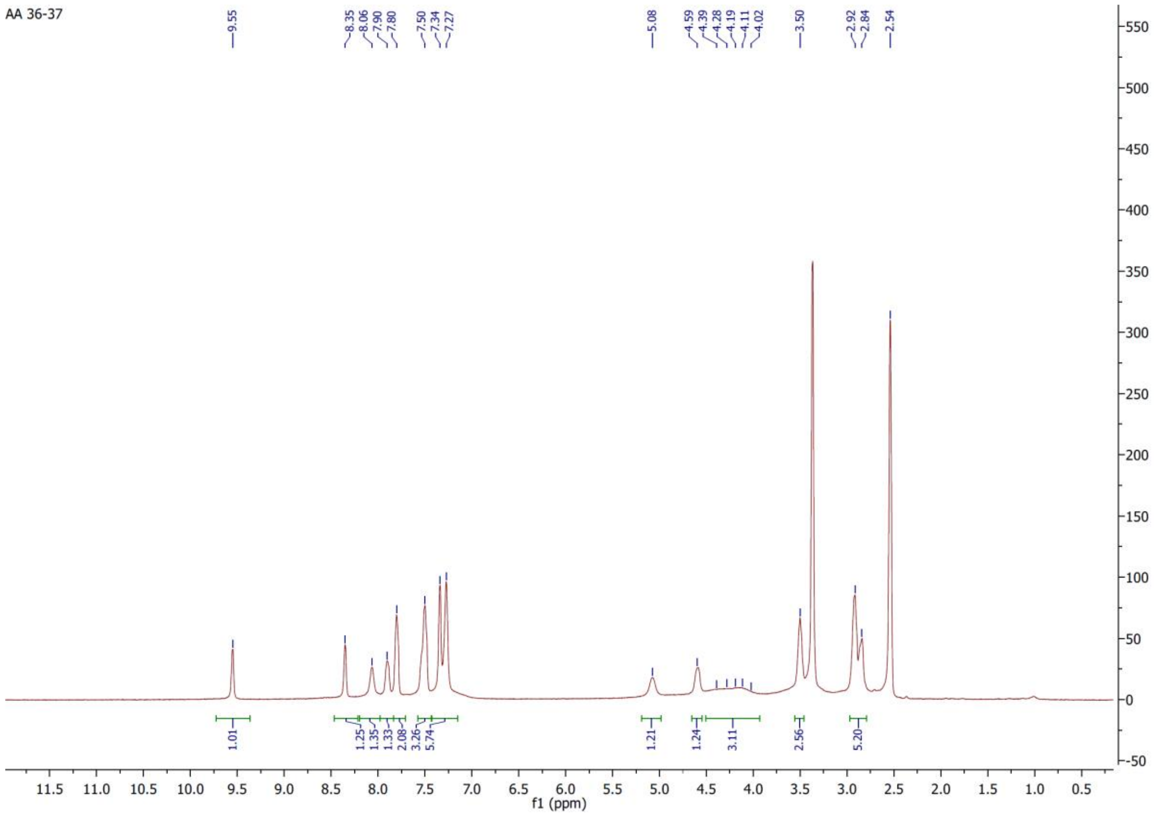

^{1}H NMR spectrum of compound **33** (400 MHz, DMSO-d_6)

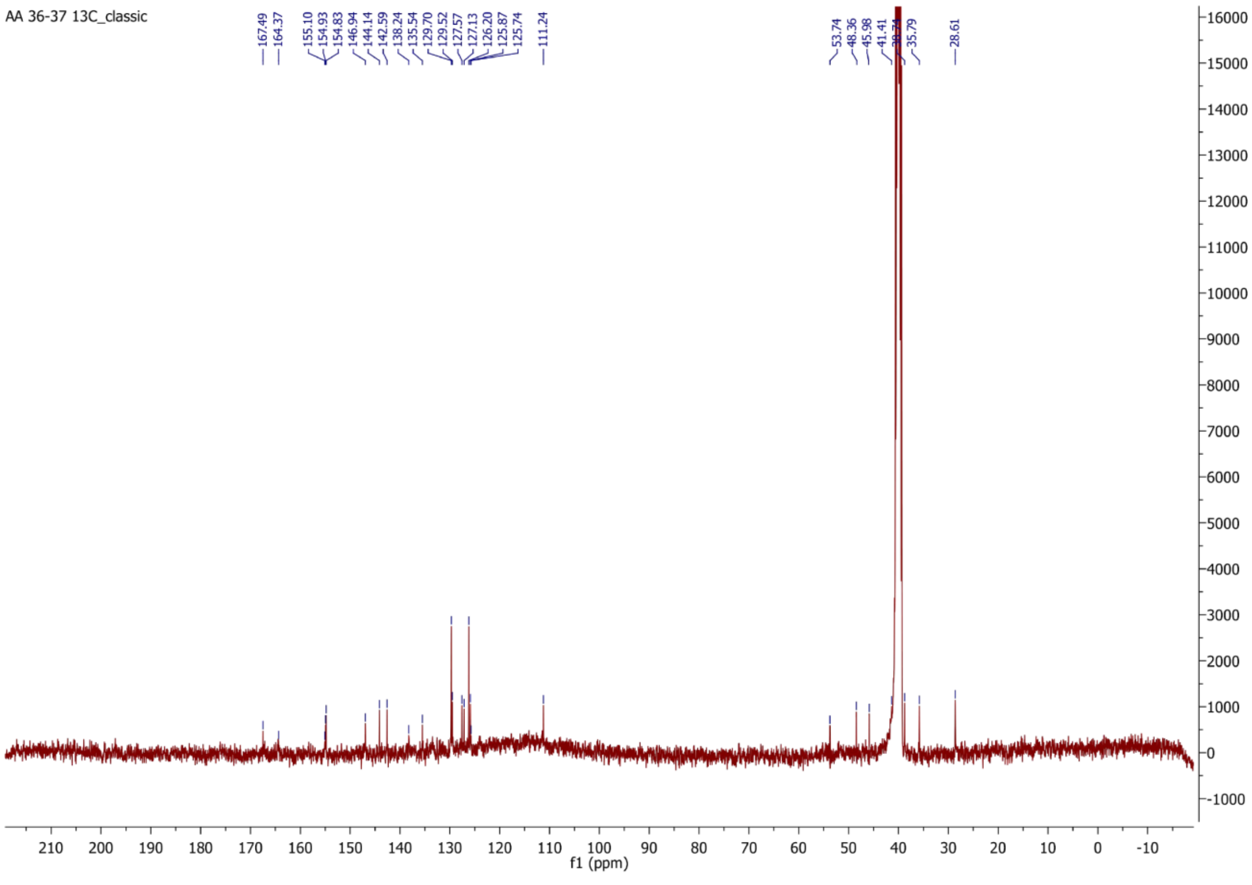

^{13}C NMR spectrum of compound **33** (100 MHz, DMSO-d_6)

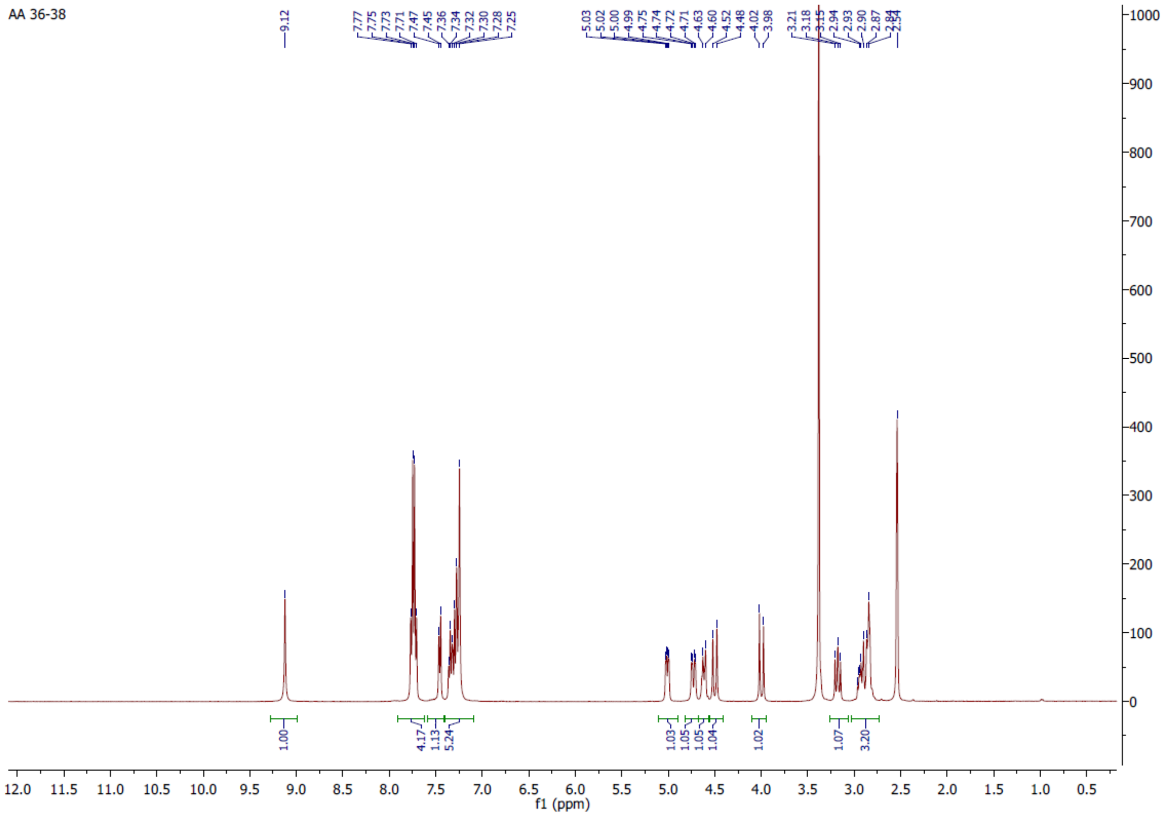

^{1}H NMR spectrum of compound **34a** (400 MHz, DMSO-d_6)

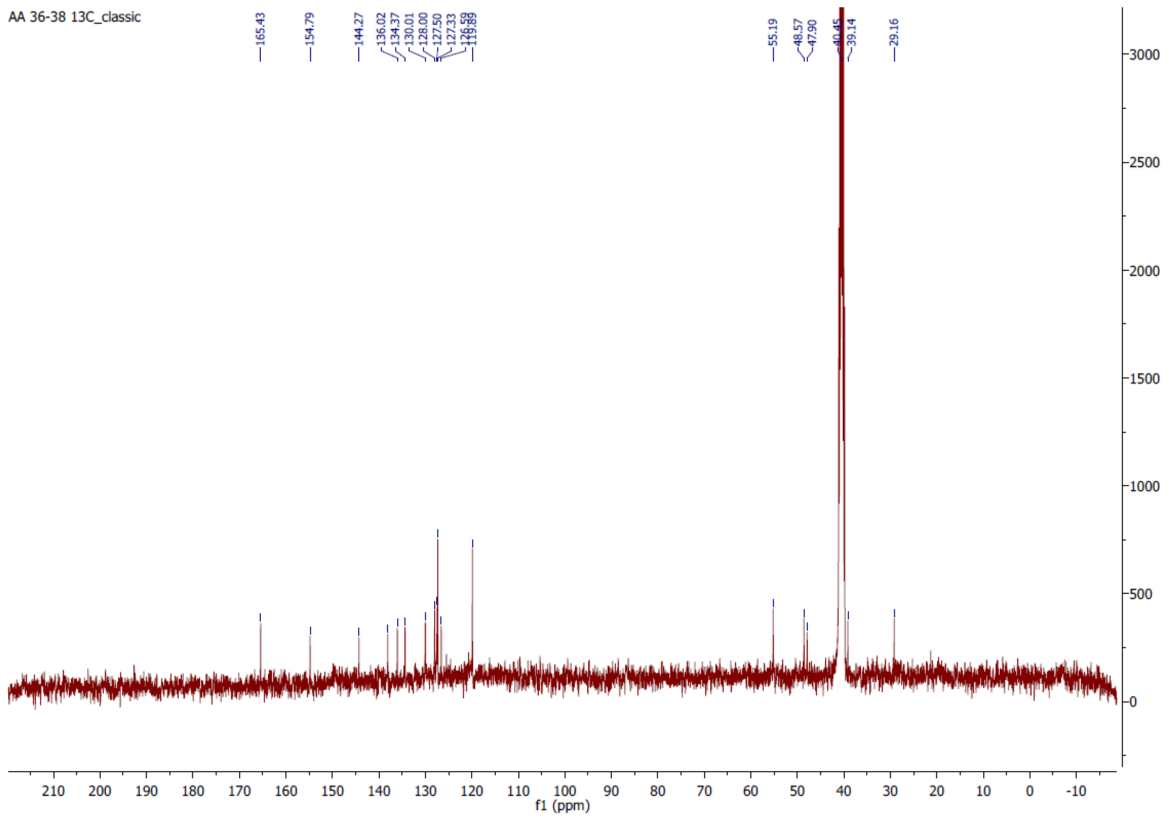

^{13}C NMR spectrum of compound **34a** (100 MHz, DMSO-d_6)

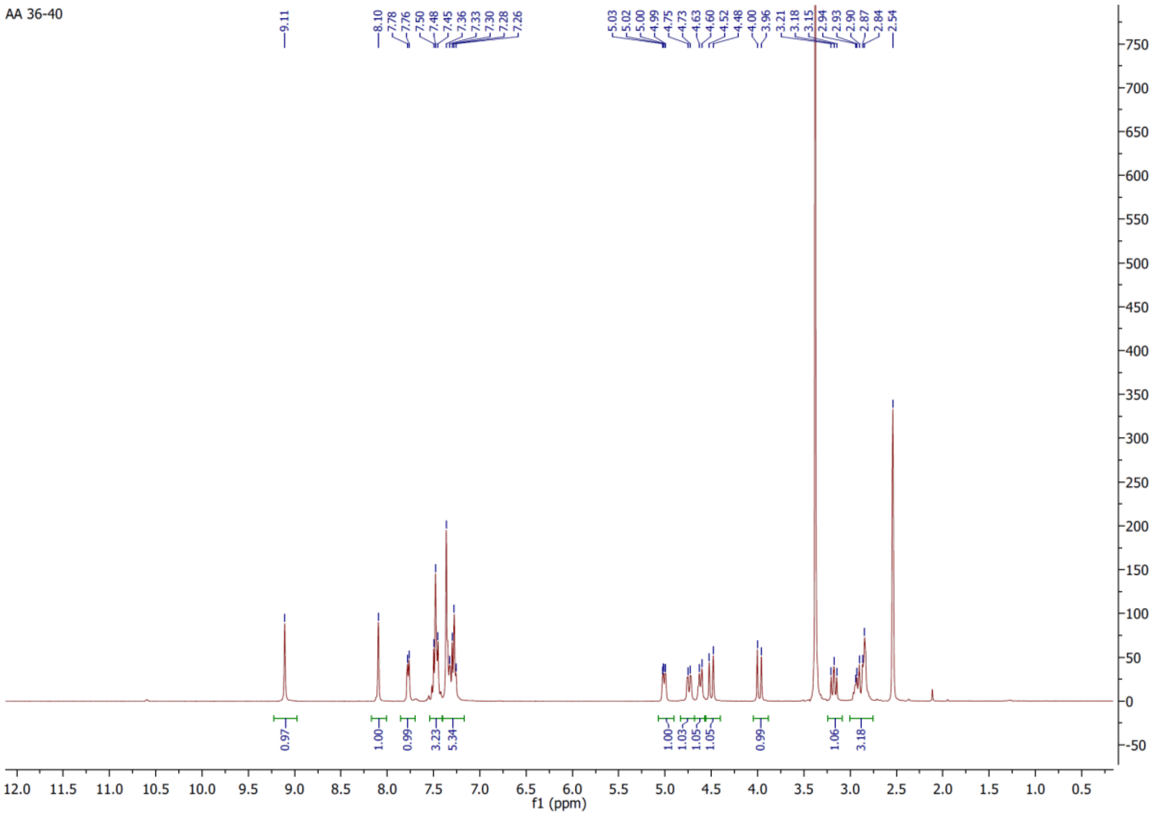

^{1}H NMR spectrum of compound **34b** (400 MHz, DMSO-d_6)

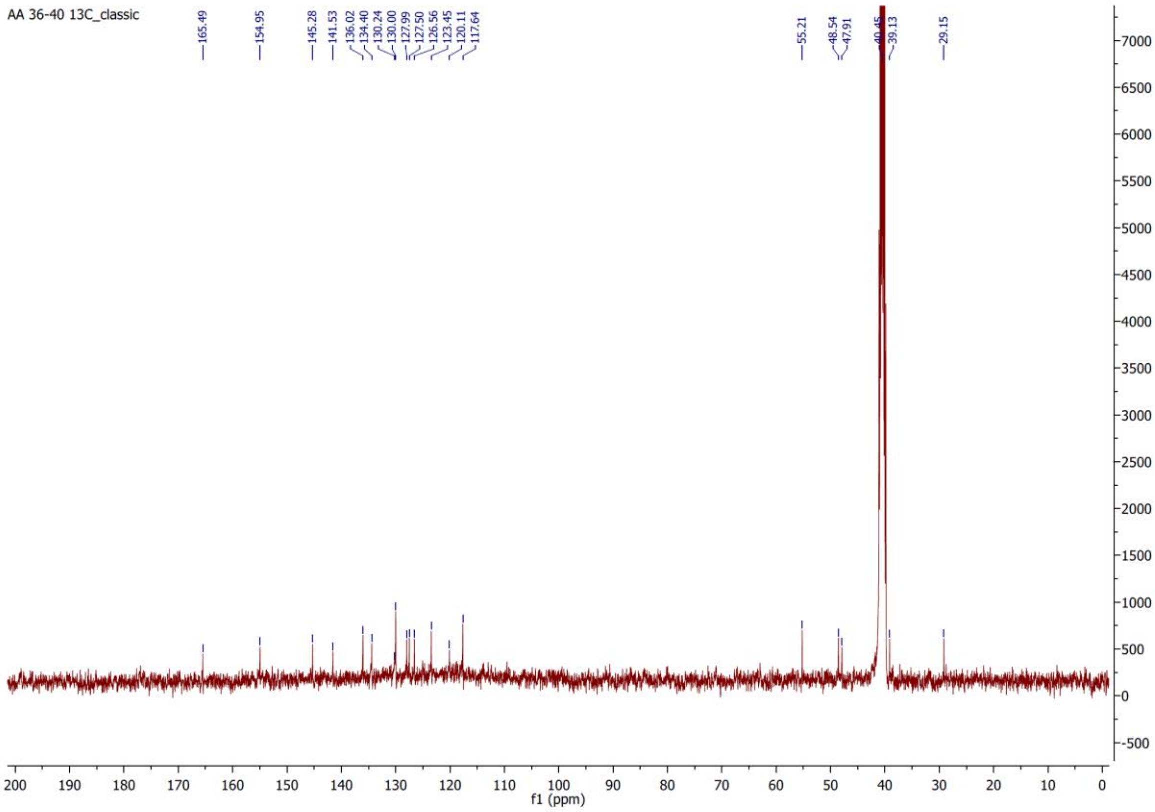

^{13}C NMR spectrum of compound **34b** (100 MHz, DMSO-d_6)

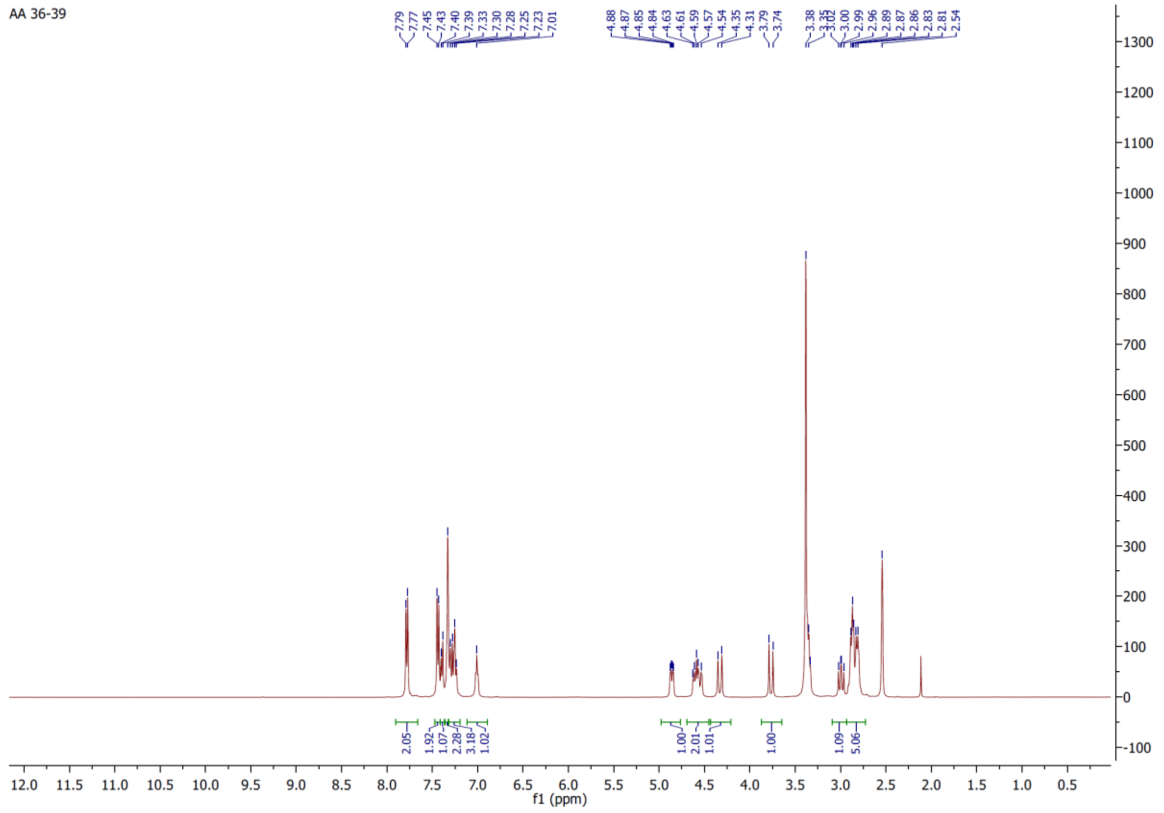

^{1}H NMR spectrum of compound **34c** (400 MHz, DMSO-d_6)

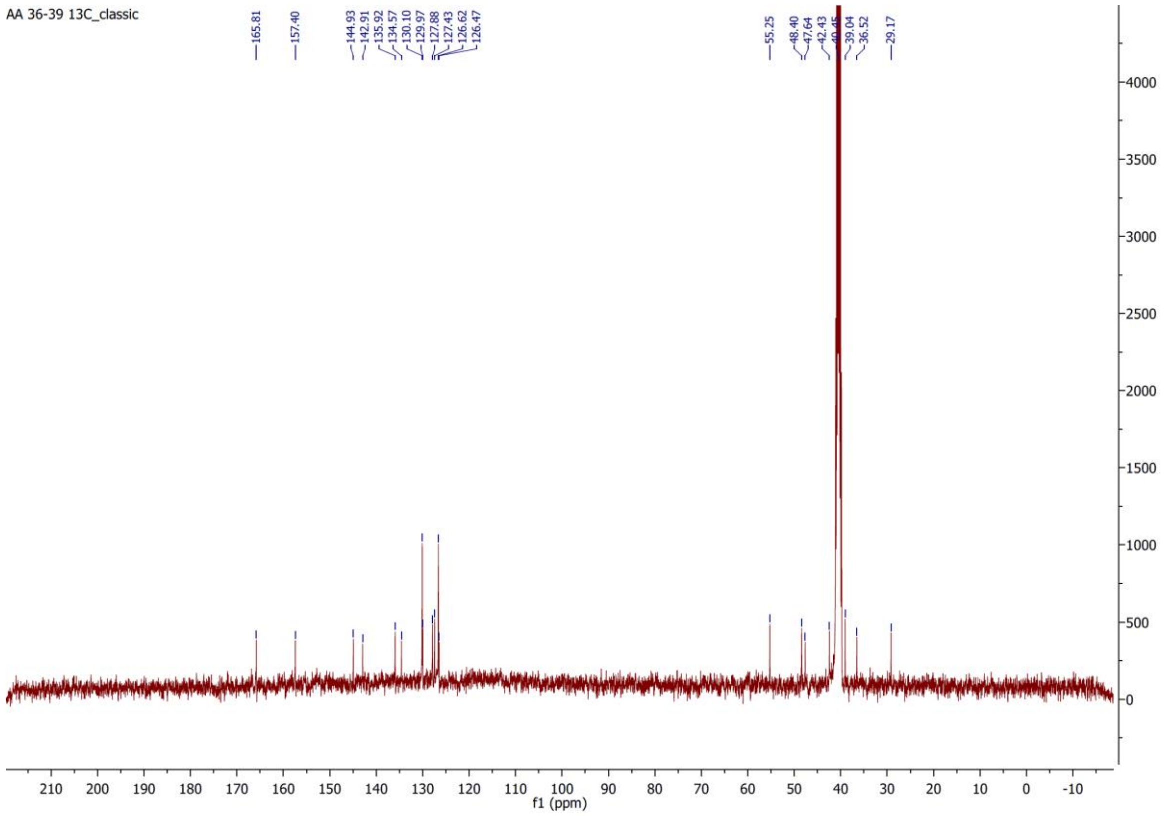

^{13}C NMR spectrum of compound **34c** (100 MHz, DMSO-d_6)

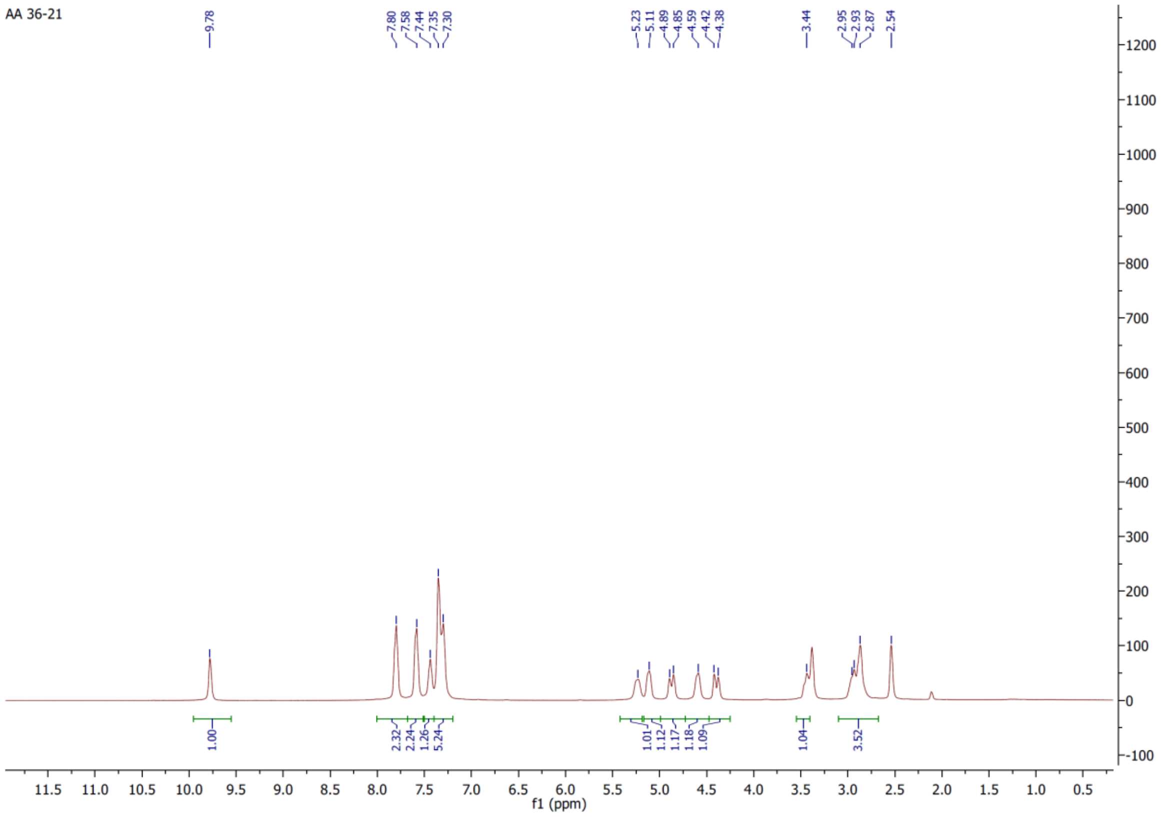

^{1}H NMR spectrum of compound **35a** (400 MHz, DMSO-d_6)

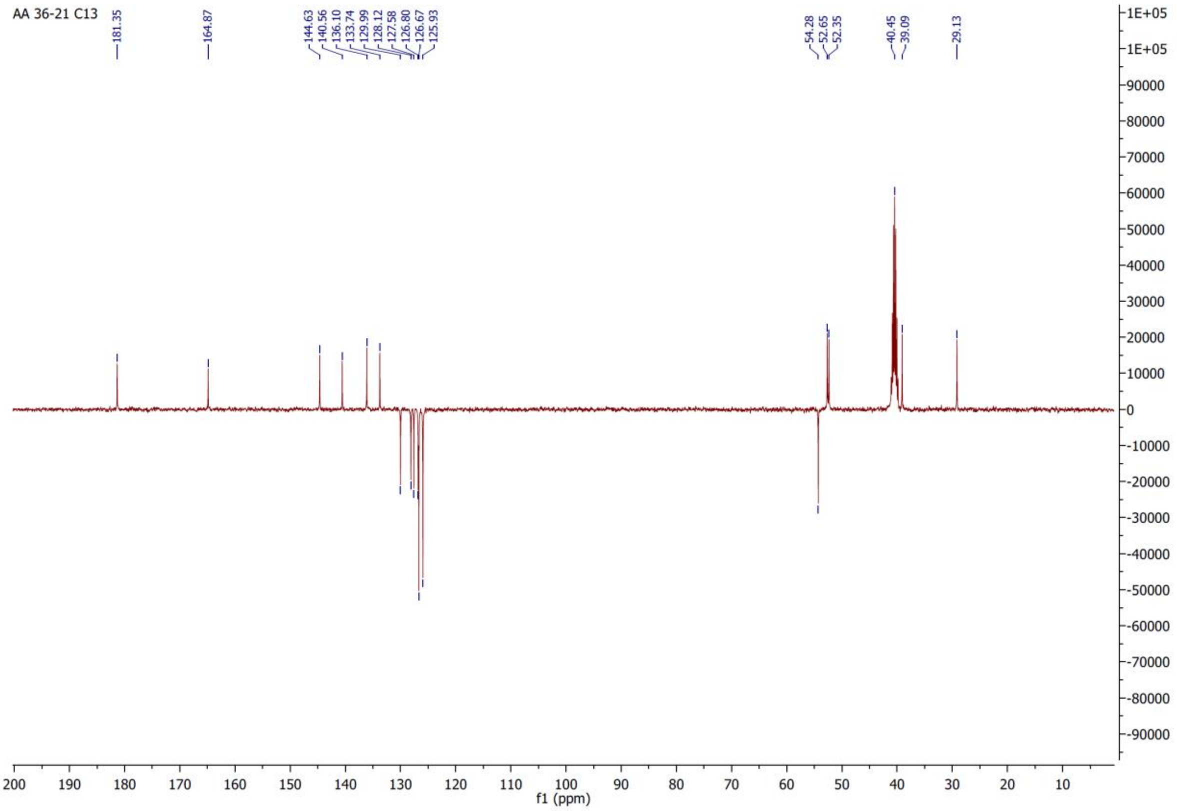

^{13}C NMR spectrum of compound **35a** (100 MHz, DMSO-d_6)

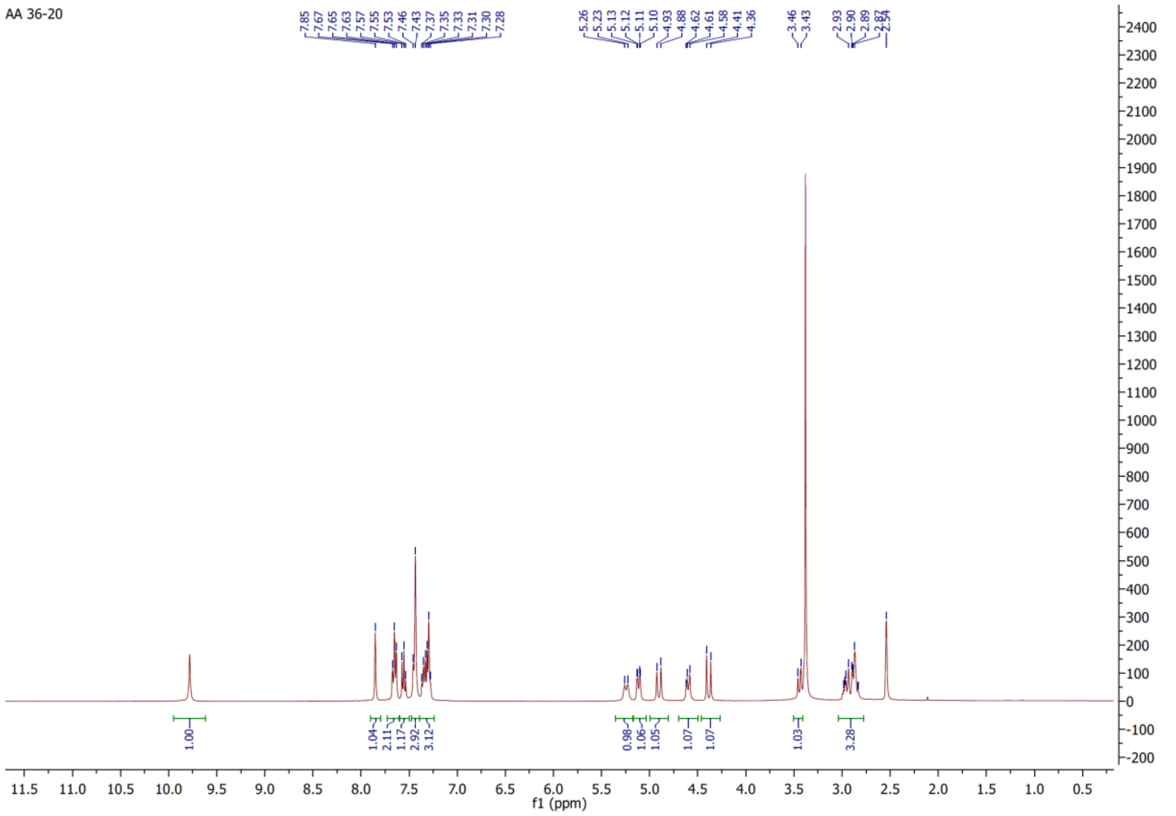

^{1}H NMR spectrum of compound **35b** (400 MHz, DMSO-d_6)

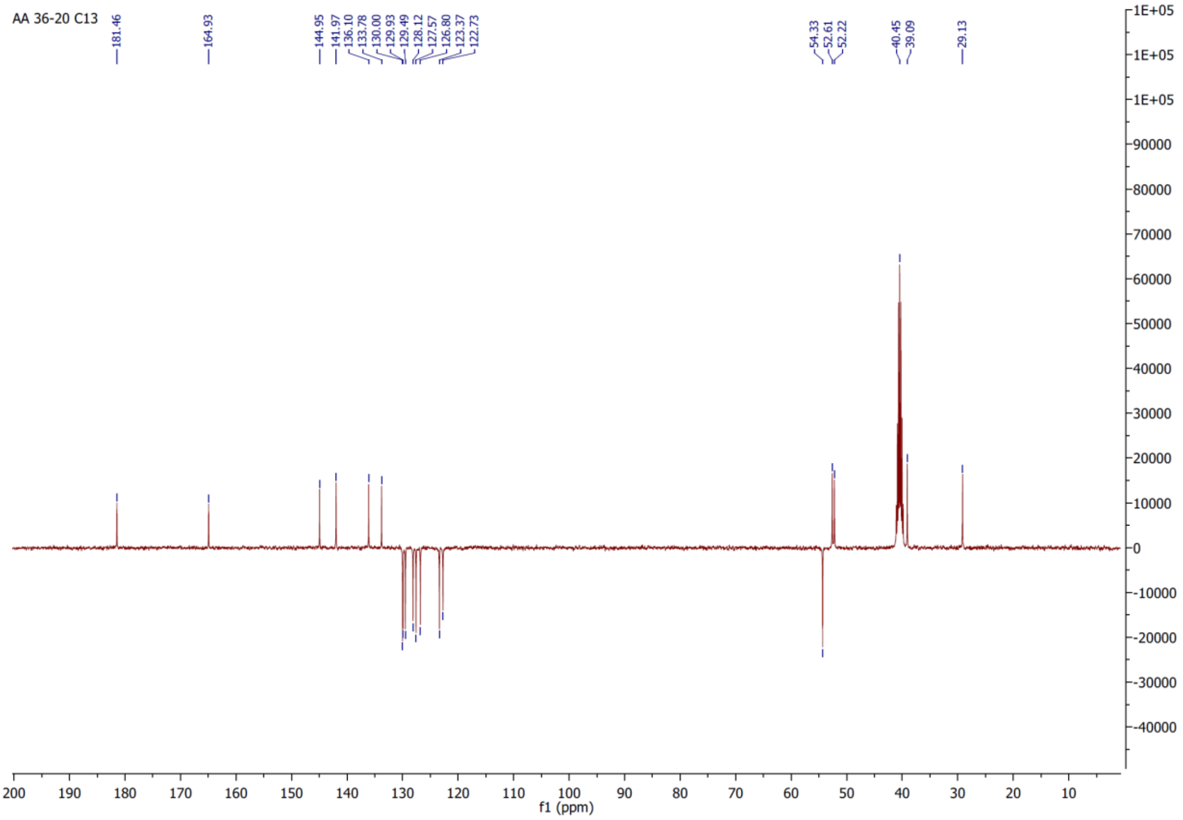

^{13}C NMR spectrum of compound **35b** (100 MHz, DMSO-d_6)

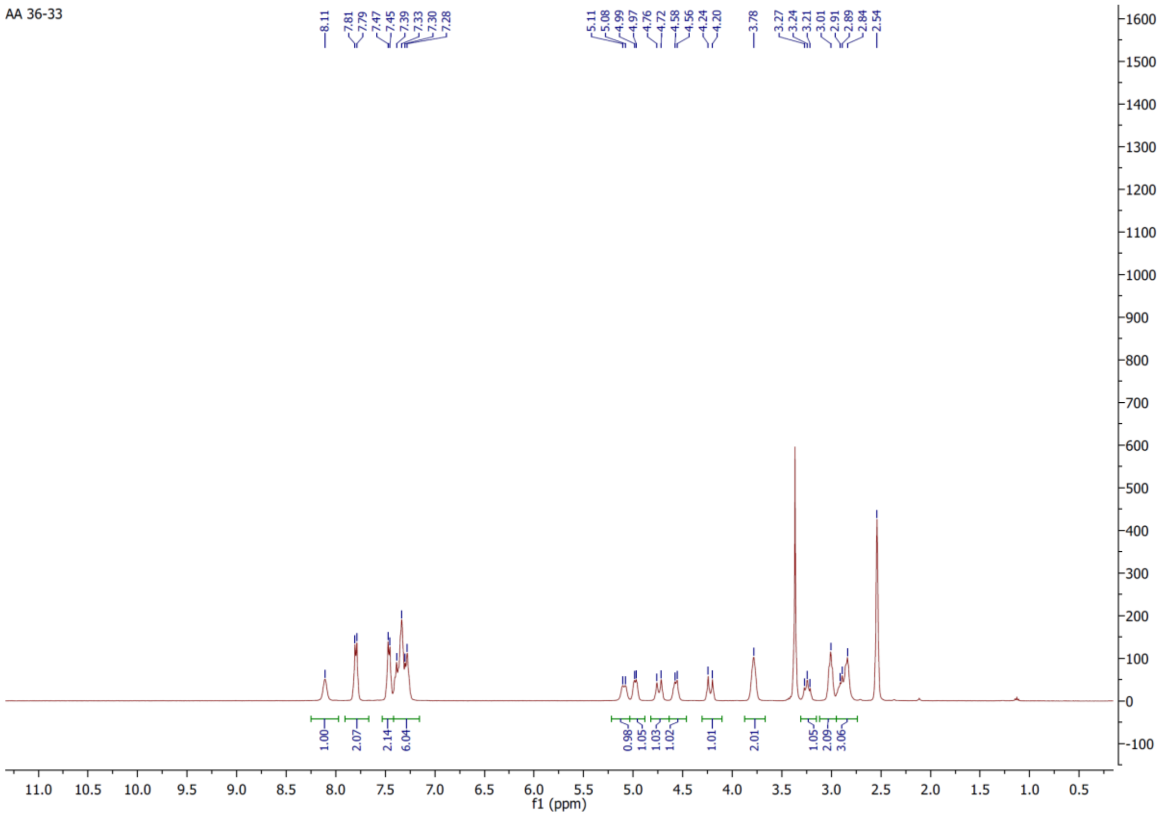

^{1}H NMR spectrum of compound **35c** (400 MHz, DMSO-d_6)

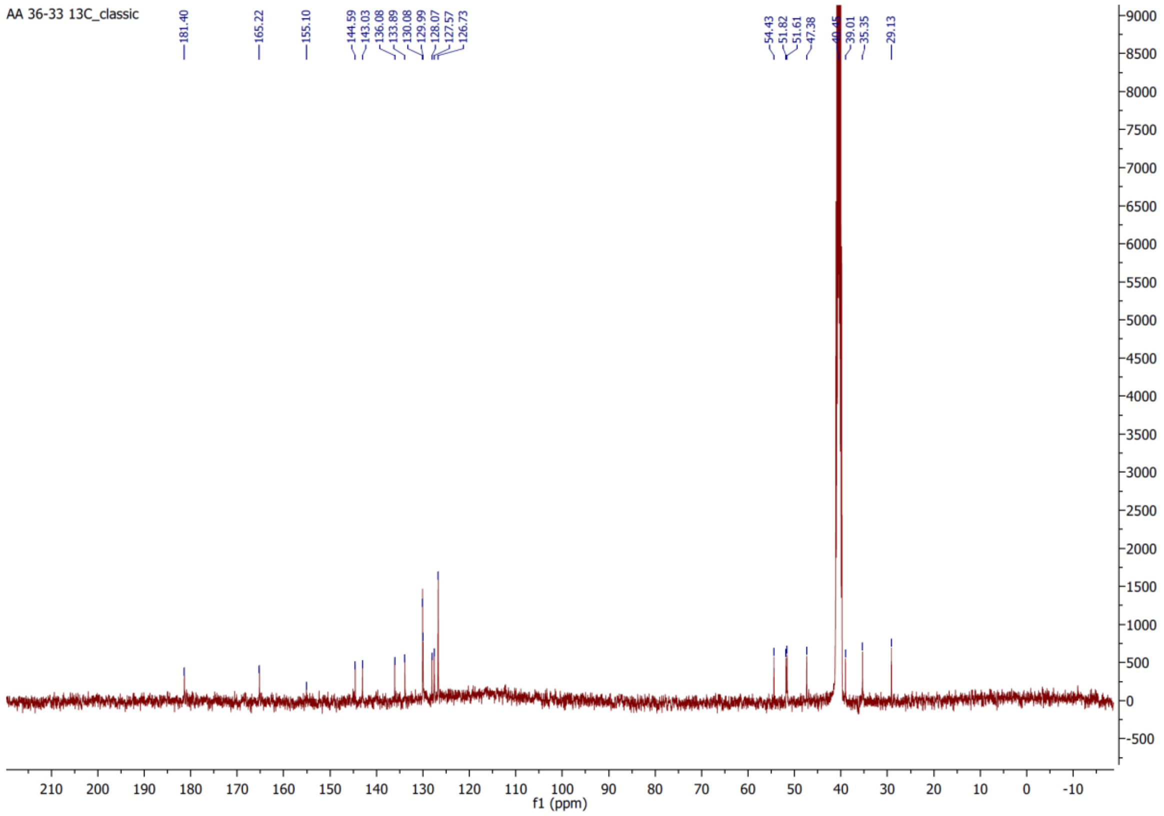

^{13}C NMR spectrum of compound **35c** (100 MHz, DMSO-d_6)

Summary of Data Collection and Atomic Model Refinement Statistics for hCAII

	**hCAII + 34a**	**hCAII + 34c**	**hCAII + 35c**
PDB ID	7YWT	7QZX	7R1X
Wavelength (Å)	0.9999	0.9718	1.00000
Space Group	P21	P21	P21
Unit cell (a, b, c, α, β, γ) (Å,°)	42.42, 41.48, 72.04, 90.000, 104.445, 90.000	42.47, 41.77, 72.10 90.000, 104.491, 90.000	42.49, 41.68, 72.08, 90.000, 104.513, 90.000
Limiting resolution (Å)	48.00-1.11(1.11-1.13)	41.15-1.24 (1.27-1.24)	41.17 -1.35 (1.35-1.38)
Unique reflections	95824 (6872)	62450 (2417)	53135 (3257)
Rmerge (%)	6.7 (95.7)	7.0 (70.4)	5.3 (63.1)
Rmeas (%)	6.8 (106.2)	7.1 (73.2)	5.4 (66.3)
Redundancy	6.14 (5.30)	6.17 (4.56)	5.83 (3.46)
Completeness overall (%)	98.8 (96.3)	88.8 (46.3)	97.8 (81.6)
<I/σ(I)>	16.40 (2.41)	13.84 (2.18)	18.60 (2.19)
CC (1/2)	99.9 (72.7)	99.8 (76.6)	99.9 (72.1)
**Refinement statistics**			
Resolution range (Å)	48.00-1.11	41.15-1.24	41.17 -1.35
Rfactor (%)	16.15	15.88	15.72
Rfree(%)	17.74	17.90	17.63
r.m.s.d. bonds(Å)	0.0167	0.0144	0.0138
r.m.s.d. angles (°)	2.0011	1.9072	1.8815
**Ramachandran statistics** (%)			
Most favored	97.3	96.9	97.7
additionally allowed	2.7	3.1	2.3
outlier regions	0.0	0.0	0.0
**Average B factor (Å^2)**			
All atoms	13.248	17.626	17.622
inhibitors	19.605	22.935	26.753
solvent	21.583	26.353	26.753

Summary of Data Collection and Atomic Model Refinement Statistics for SmCA

	**SmCA + 35c**
PDB ID	7YZH
Wavelength (Å)	0.971800
Space Group	$P3_221$
Unit cell (a, b, c, α, β, γ) (Å,°)	103.83, 103.83, 133.06, 90.00, 90.00, 120.00
Limiting resolution (Å)	48.0-1.79 (1.83-1.79)
Unique reflections	79049 (5786)
Rmerge (%)	8.6 (145.5)
Rmeas (%)	8.9 (149.7)
Redundancy	19.13 (17.97)
Completeness overall (%)	100.0 (100.0)
<I/σ(I)>	24.04 (2.32)
CC (1/2)	100 (81.0)
**Refinement statistics**	
Resolution range (Å)	48.0-1.79
Rfactor (%)	16.79
Rfree(%)	19.80
r.m.s.d. bonds(Å)	0.0129
r.m.s.d. angles (°)	1.8020
**Ramachandran statistics** (%)	
Most favored	96.9
additionally allowed	2.9
outlier regions	0.2
**Average B factor ($Å^2$)**	
All atoms	33.874
inhibitors	74.323
solvent	38.125

Figure S1:

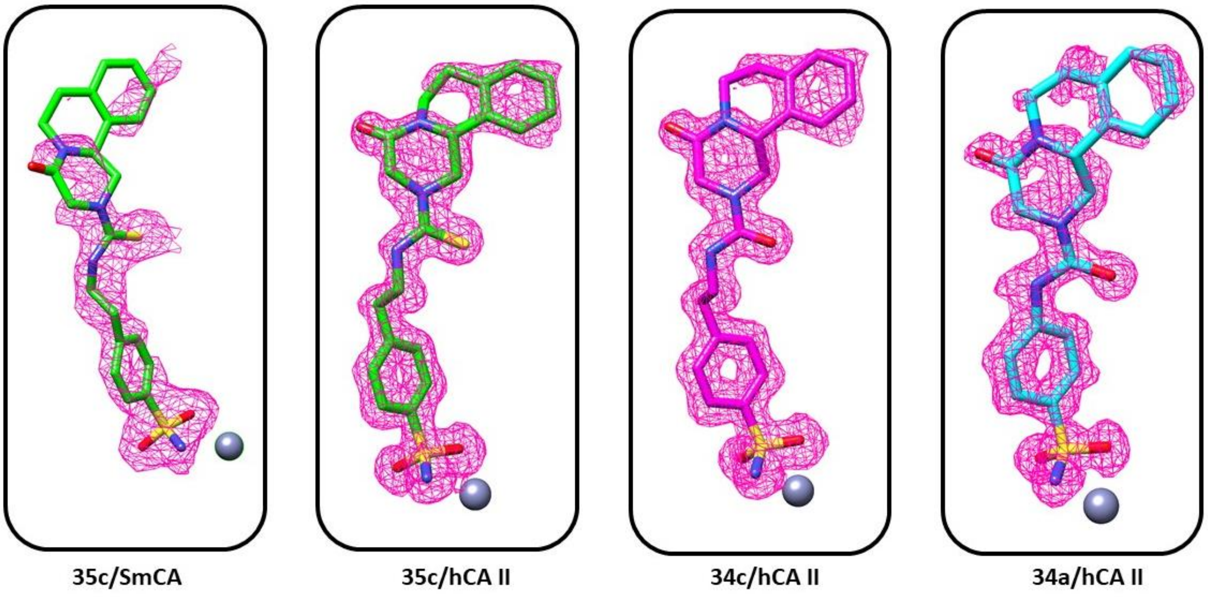

Electron density of inhibitors bound to zinc (grey) in SmCA and hCA II active site. $2F_o$-F_c maps and contoured to the 1.0 σ level.
